# The effect of enzyme replacement therapy on clinical outcomes in male patients with Fabry disease: A systematic literature review by a European panel of experts

**DOI:** 10.1016/j.ymgmr.2019.100454

**Published:** 2019-02-06

**Authors:** Dominique P. Germain, Perry M. Elliott, Bruno Falissard, Victor V. Fomin, Max J. Hilz, Ana Jovanovic, Ilkka Kantola, Aleš Linhart, Renzo Mignani, Mehdi Namdar, Albina Nowak, João-Paulo Oliveira, Maurizio Pieroni, Miguel Viana-Baptista, Christoph Wanner, Marco Spada

**Affiliations:** a French Referral Center for Fabry disease, Division of Medical Genetics and INSERM U1179, University of Versailles, Paris-Saclay University, Montigny, France; b University College London and Barts Heart Centre, London, United Kingdom; c INSERM U1018, University of Paris-Sud, University of Paris-Descartes, Paris, France; d Department of Internal Diseases No.1, I.M. Sechenov First Moscow State Medical University, Moscow, Russian Federation; e Department of Neurology, University of Erlangen-Nuremberg, Erlangen, Germany; f Mark Holland Metabolic Unit, Salford Royal NHS Foundation Trust, Salford, United Kingdom; g Division of Medicine, Turku University Hospital, University of Turku, Turku, Finland; h Second Department of Medicine – Department of Cardiovascular Medicine, 1st Faculty of Medicine, Charles University and General University Hospital, Prague, Czech Republic; i Department of Nephrology, Infermi Hospital, Rimini, Italy; j Service de Cardiologie, Hôpitaux Universitaires de Genève, Geneva, Switzerland; k Department of Internal Medicine, University Hospital of Zurich and University of Zurich, Zurich, Switzerland; l Department of Genetics, São João Hospital Centre and Faculty of Medicine and “Instituto de Investigação e Inovação em Saúde (iS3)”, University of Porto, Porto, Portugal; m Cardiovascular Department, San Donato Hospital, Arezzo, Italy; n Serviço de Neurologia, Hospital Egas Moniz, Centro Hospitalar de Lisboa Ocidental; CEDOC Faculdade de Ciências Médicas, Universidade Nova de Lisboa, Lisboa, Portugal; o Division of Nephrology, University Clinic, University of Würzburg, Würzburg, Germany; p Department of Paediatrics, University of Torino, Torino, Italy

**Keywords:** Fabry disease, agalsidase alfa, agalsidase beta, systematic literature review, enzyme replacement therapy, adult male patients, ANS, autonomic nervous system, ACEi, angiotensin-converting enzyme inhibitor, ARB, angiotensin receptor blocker, BPI, Brief Pain Inventory, CES-D, Center for Epidemiologic Studies Depression Scale, CNS, central nervous system, CR, case report, CT, clinical trial, ECG, electrocardiogram/electrocardiography, eGFR, estimated glomerular filtration rate, EOW, every other week, ERT, enzyme replacement therapy, GFR, glomerular filtration rate, GI, gastrointestinal, GL-3, globotriaosylceramide, IVST, intraventricular septum thickness, IENFD, intra-epidermal nerve fibre density, LPWT, left posterior wall thickness, LVEDD, left ventricular end-diastolic diameter, LVEF, left ventricular ejection fraction, LVH, left ventricular hypertrophy, LVM, left ventricular mass, LVMi, left ventricular mass index, LVWT, left ventricular wall thickness, lyso-GL-3, globotriaosylsphingosine, MG, mixed gender, MRI, magnetic resonance imaging, MWT, maximal wall thickness, NYHA, New York Heart Association, OS, observational study, PNS, peripheral nervous system, QoL, quality of life, RCT, randomized controlled trial, SF-36, 36-item Short Form Health Survey, TIA, transient ischaemic attack, WMH, white matter hyperintensities.

## Abstract

**Background:**

Enzyme replacement therapy (ERT) with recombinant human α-galactosidase has been available for the treatment of Fabry disease since 2001 in Europe and 2003 in the USA. Treatment outcomes with ERT are dependent on baseline patient characteristics, and published data are derived from heterogeneous study populations.

**Methods:**

We conducted a comprehensive systematic literature review of all original articles on ERT in the treatment of Fabry disease published up until January 2017. This article presents the findings in adult male patients.

**Results:**

Clinical evidence for the efficacy of ERT in adult male patients was available from 166 publications including 36 clinical trial publications. ERT significantly decreases globotriaosylceramide levels in plasma, urine, and in different kidney, heart, and skin cell types, slows the decline in estimated glomerular filtration rate, and reduces/stabilizes left ventricular mass and cardiac wall thickness. ERT also improves nervous system, gastrointestinal, pain, and quality of life outcomes.

**Conclusions:**

ERT is a disease-specific treatment for patients with Fabry disease that may provide clinical benefits on several outcomes and organ systems. Better outcomes may be observed when treatment is started at an early age prior to the development of organ damage such as chronic kidney disease or cardiac fibrosis. Consolidated evidence suggests a dose effect. Data described in male patients, together with female and paediatric data, informs clinical practice and therapeutic goals for individualized treatment.

## Highlights

• This article provides a systematic review of the available clinical evidence on ERT in adult male Fabry patients.
• The patient population heterogeneity highlights the need for standardized reporting and population-specific guidelines.
• ERT may slow down progression of cardiac and renal disease and has a positive effect on other outcomes.
• Treatment effects can be optimized when ERT is started as early as possible before the onset of major organ damage.
• Consolidated evidence suggests a dose effect of ERT.
• Therapeutic goals for the treatment of Fabry disease with ERT help advance disease management.

## 1 Introduction

Fabry disease (OMIM #301500) is an X-linked lysosomal storage disorder caused by pathogenic variants in the *GLA* gene (OMIM #300644; HGNC 4296) encoding the lysosomal enzyme α-galactosidase [1]. Subsequent accumulation of the glycosphingolipid globotriaosylceramide (GL-3) and its derivative globotriaosylsphingosine (lyso-GL-3) in cells, plasma, and urine causes progressive tissue damage in affected organs, resulting in multisystemic disease, life-threatening complications, and a reduced life expectancy in both males and females [2].

Fabry disease has a wide range of clinical presentations ranging from the early-onset ‘classic’ severe phenotype in patients with absent or severely decreased α-galactosidase activity, to later-onset ‘non-classic’ phenotypes often affecting a single organ system in patients with higher levels of residual α-galactosidase activity [1,3,4]. Patients with the classic phenotype, who are mostly males, generally experience signs and symptoms from early childhood onwards such as neuropathic pain, gastrointestinal (GI) disturbance, and hypohidrosis (all likely due to peripheral and autonomic nervous system [PNS, ANS] involvement), progressing to multi-organ failure involving the kidneys (albuminuria, proteinuria, decline in glomerular filtration rate [GFR], kidney failure), heart (left ventricular hypertrophy [LVH], heart failure, conduct abnormalities, and arrhythmias), auditory/vestibular system (hearing loss), and central nervous system (CNS) (stroke) in adulthood [1,[5], [6], [7], [8], [9], [10], [11], [12], [13], [14], [15]].

Enzyme replacement therapy (ERT) with recombinant α-galactosidase was approved in Europe in 2001. There are two preparations available: agalsidase alfa (Replagal®) administered at the licensed dose of 0.2 mg/kg; and agalsidase beta (Fabrazyme®) administered at the licensed dose of 1 mg/kg body weight. Both preparations are administered intravenously every other week (EOW) [16,17]. Agalsidase alfa and agalsidase beta are available in most European countries, and in Asia, Australia, and Canada. Agalsidase beta was approved by the US Food and Drug Administration in 2003.

Although ERT has been in clinical use since 2001, many questions remain regarding treatment initiation timing, optimal dose, and treatment goals [1,17]. This is important as ERT in Fabry disease is expensive and is a lifelong commitment for patients. Traditionally used methods for analysing pooled data such as meta-analysis and meta-syntheses are difficult to apply in rare disease settings [[18], [19], [20]] and a systematic literature analysis including real-life experiences may be the best tool with which to provide a comprehensive overview of published clinical evidence.

We conducted a comprehensive systematic literature review of all original articles on ERT in the treatment of Fabry disease published up until January 2017 [21]. This article presents an analysis of treatment outcomes in adult male patients.

## 2 Methods

The full methodology for the systematic literature searches that were performed has been published in this issue [21], together with papers summarizing the findings of the literature review in female [22] and paediatric patients [23], and a position statement on therapeutic goals in Fabry disease based on the conclusions of an expert consensus panel [24]. The original searches included articles published up to and including January 2017.

The outcomes that were selected for analysis included plasma and urine GL-3 and lyso-GL-3 levels, GL-3 histology, measures of renal and cardiac function and of cardiac morphology. Other outcomes included ANS, PNS, and CNS parameters, GI symptoms, pain, and quality of life (QoL). GL-3 levels were described as ‘normalized’ if they were higher than reference values at baseline and decreased to within reference values during treatment, and if they were described as being normalized in the publication; note that the reference values varied in each publication.

Results are described for the approved dose regimens agalsidase alfa 0.2 mg/kg EOW and agalsidase beta 1.0 mg/kg EOW. Specific note has been made of altered dose regimens due to the temporary shortage of agalsidase beta to examine the efficacy of reduced-dose ERT [198]. Publications describing studies in which data from patients treated with agalsidase alfa and agalsidase beta were combined or in which the ERT type was not specified are referred to in the analysis as ‘mixed-ERT’ publications.

## 3 Results

### 3.1 Adult male population and publication overview

The publications that reported ERT outcomes data specific for adult male patients and that were included in the systematic literature analysis are summarized in Supplementary Table 1a. Publications were available from clinical trials (CT), observational study (OS) publications, studies describing both treatments, a combination of treatments, or when treatment was not specified (i.e. mixed-ERT studies), studies including both genders but with at least 50% male patients (i.e. mixed-gender [MG] studies), and case reports (CR).

For agalsidase alfa there were 16 CT publications (including five publications derived from Grade 1a randomized controlled trials [RCTs], nine publications from Grade 1c single-arm CTs and two from Grade 1a/c studies; four were placebo-controlled studies), 15 OS publications (of which six covered Grade 2 prospective OS publications, and nine Grade 3 retrospective OS publications) and 13 CRs (three Grade 4 case series and 10 Grade 5 CRs). For agalsidase beta, there were five CT publications (all Grade 1c single-arm CTs), 11 OS publications (four Grade 2 prospective OS publications, and seven Grade 3 retrospective OS publications) and 17 CRs (four Grade 4 case series and 13 Grade 5 CRs). There were no CT publications, 18 OS publications (seven Grade 2 prospective OS publications, and 11 Grade 3 retrospective OS publications), and 19 CRs (five Grade 4 case series and 12 publications which were either Grade 5 CRs or reported baseline and follow-up data on a single patient) describing mixed ERT, a comparison between agalsidase alfa and agalsidase beta, or not specifying the type of ERT.

The publications that reported ERT outcomes data for MG publications are summarized in Supplementary Table 1b. For agalsidase alfa there were five CT publications (two from Grade 1a RCTs, two from Grade 1c single-arm CTs and one from a Grade 1a/c study [1a portion was placebo-controlled]), 11 OS publications (five from Grade 2 prospective OS publications, and six from Grade 3 retrospective OS publications), and no CRs. For agalsidase beta, there were ten CT publications (one placebo-controlled Grade 1a RCT, seven Grade 1c single-arm CTs [including four open-label extension studies of the RCT] and two Grade 1a/c studies [where the 1a portion was placebo-controlled]), 19 OS publications (16 Grade 2 prospective OS publications, and three Grade 3 retrospective OS publications) and no CRs. For mixed ERT there were two CT publications (one from a Grade 1a RCT and another from a Grade 1c single-arm CT), 14 OS publications (nine from Grade 2 prospective OS publications, and five from Grade 3 retrospective OS publications) and no CRs.

The main findings regarding clinical outcomes of treatment with agalsidase alfa and agalsidase beta in male patients with Fabry disease are summarized in Table 1.

**Table 1:** Summary of outcomes with agalsidase beta 1.0 mg/kg EOW or agalsidase alfa 0.2 mg/kg EOW from clinical and observational studies of adult male patients with Fabry disease

Outcome		Agalsidase alfa 0.2 mg/kg EOW	Agalsidase beta 1.0 mg/kg EOW
3.2. GL-3 and lyso-GL-3 accumulation			
3.2.1.	Plasma GL-3	↓ Significant (Clarke et al. 2007 [25]; Pastores et al. 2007 [35]; Schiffmann et al. 2006 [39]; Schiffmann et al. 2007 [52]; van Breemen et al. 2011 [102]) ↓ Significance unknown (Goker-Alpan et al. 2015 [26]; Goláň et al. 2015 [27]; Hughes et al. 2008 [29]; Schiffmann et al. 2001 [36]; Rombach et al. 2012 [101]) – No change (Goker-Alpan et al. 2015 [26 (switch study)])	↓ Significant (Eto et al. 2005 [70]; Goker-Alpan et al. 2016 [71 (switch study)]; Lubanda et al. 2009 [72]; Elliott et al. 2006 [76]; van Breemen et al. 2011 [102]) ↓ Non-significant (Bénichou et al. 2009 [75]; Mignani et al. 2004 [80]) ↓ Unknown significance (Eng et al. 2001 [69]; Rombach et al. 2012 [101])
3.2.2.	Plasma lyso-GL-3	↓ Significant (Goker-Alpan et al. 2015 [26 (switch study)]) ↓ Unknown significance (Rombach et al. 2012 [101]; van Breemen et al. 2011 [102]) ↑ Significant (Smid et al. 2011 [54 (switch study)])	↓ Significant (Goker-Alpan et al. 2016 [71 (switch study)]) ↓ Unknown significance (Rombach et al. 2012 [101]; van Breemen et al. 2011 [102])
3.2.3.	Urinary GL-3	↓ Significant (Goker-Alpan et al. 2015 [26 (12 months)]; Hughes et al. 2008 [29 (6 months)]; Schiffmann et al. 2001 [36]; Schiffmann et al. 2006 [38]; Schiffmann et al. 2007 [52]) ↓ Unknown significance (Pastores et al. 2007 [35 (1/12 pts)]; Whitfield et al. 2005 [55]; Rombach et al. 2012 [101]) ↑ Non-significant (Goker-Alpan et al. 2015 [26 (switch study)])	↓ Significant (Lubanda et al. 2009 [72]) ↓ Non-significant (Eto et al. 2005 [70]; Goker-Alpan et al. 2016 [71 (switch study)]) ↓ Unknown significance (Rombach et al. 2012 [101]) – No change (Mignani et al. 2004 [80])
3.2.4.	Urinary lyso-GL-3	No data from clinical or observational studies with approved dose of agalsidase alfa	No data from clinical or observational studies with approved dose of agalsidase beta

3.2.5. GL-3 accumulation: histology			
3.2.5.1.	Renal cells	↓ Non-significant (Schiffmann et al. 2001 [36])	↓Significant (Lubanda et al. 2009 [72]; Najafian et al. 2016 [73 (proportion of podocytes without GL-3 inclusions)]) ↓ Non-significant (Eng et al. 2001 [69]; Najafian et al. 2016 [73 (volume of GL-3 inclusions/podocyte and podocyte GL-3 score)])
3.2.5.2.	Cardiac cells	↓ Significance unknown (Hughes et al. 2008 [29])	↓ Significance unknown (Eng et al. 2001 [69])
3.2.5.3.	Other cell types	No data from clinical or observational studies with approved dose of agalsidase alfa	↓ Significance unknown (Eng et al. 2001 [69]; Lubanda et al. 2009 [72]; Bénichou et al. 2009 [75])

3.3. Renal outcomes			
3.3.1.	eGFR	↑ Significant (Hughes et al. 2008 [29]) Significantly slower decline (vs placebo, creatinine clearance (Schiffmann et al. 2001 [36]) ↓ Significant (Schiffmann et al. 2006 [39]; Feriozzi et al. 2009 [43]; Feriozzi et al. 2012 [44]; Hughes et al. 2011 [48]; Schiffmann et al. 2007 [52]) ↓ Non-significant (Schiffmann et al. 2006 [38]; Smid et al. 2011 [54 (switch study)]) ↓ Unknown significance (Beck et al. 2015 [41]) – No change (Kampmann et al. 2015 [49])	↓ Non-significant (Eto et al. 2005 [70]) ↓ Significance unknown, reduced decline (Warnock et al. 2012 [82]) ↓ Significance unknown (Kim et al. 2016 [79]) – No change (Lubanda et al. 2009 [72])
3.3.2.	Albuminuria/ Proteinuria	↑ Significant, CKD1 pts (Feriozzi et al. 2012 [44]; Kampmann et al. 2015 [49 (pts with baseline proteinuria)]) ↑ Unknown significance (Schiffmann et al. 2006 [39]) ↓ Non-significant (Bongiorno et al. 2003 [42]) – No change, stabilization (Schiffmann et al. 2001 [36]; Feriozzi et al. 2009 [43]; Feriozzi et al. 2012 [44 (CKD2/3)]; Hughes et al. 2011 [48]; Kampmann et al. 2015 [49 (pts without baseline proteinuria)]; Schiffmann et al. 2007 [52])	– No change (Lubanda et al. 2009 [72]; Kim et al. 2016 [79]) ↓ Non-significant (Najafian et al. 2016 [73])

3.4. Cardiac outcomes			
3.4.1.	LVM/LVMi	↓ Significant improvement (Hughes et al. 2008 [29]; Hughes et al. 2011 [48 (pts with baseline LVH]); Kampmann et al. 2015 [49 (pts with baseline LVH)]) ↓ Improvement of unknown significance (Tsuboi et al. 2012 [189]) ↑ Significant (Beck et al. 2015 [41 (slowed progression)]) ↑ Non-significant (Goláň et al. 2015 [27]) – No change (Kampmann et al. 2015 [49 (pts without baseline LVH)])	↓ Significant improvement (Germain et al. 2013 [7]; Motwani et al. 2012 [81]) — No change (Elliott et al. 2006 [76]; Kim et al. 2016 [79]; Mignani et al. 2004 [80])
3.4.2.	LVWT	↓ Significant (Kampmann et al. 2015 [49]) ↓ Improvement of unknown significance (Tsuboi et al. 2012 [189])	↓ Significant (Motwani et al. 2012 [81]) ↓ Non-significant (Mignani et al. 2004 [80 (2/3 patients)]) ↑ Non-significant (Mignani et al. 2004 [80 (1/3 patients)]) — No change (Elliott et al. 2006 [76])
3.4.3.	LVEF	– No change (Hughes et al. 2008 [29]; Kampmann et al. 2015 [49])	↑ Significant (Motwani et al. 2012 [81]) ↑ Non-significant improvement (Mignani et al. 2004 [80 (1/3 patients)])
3.4.4.	ECG measures	↓ Significant improvement in QRS duration (Schiffmann et al. 2001 [36]) ↓ Non-significant improvement in QRS duration (Hughes et al. 2008 [29]) – Conduction abnormality (Kampmann et al. 2015 [49 (1/21 pts)])	↑ Significant improvement in PQ interval, P-wave duration (Motwani et al. 2012 [81]) ↑ Significant improvement (Eng et al. 2001 [69]) ↓ Significant improvement of QTc interval (Motwani et al. 2012 [81]) – No change (Mignani et al. 2004 [80])
3.4.5.	Exercise testing	No data from clinical or observational studies with approved dose of agalsidase alfa	No data from clinical or observational studies with approved dose of agalsidase beta

3.5. Nervous system outcomes			
3.5.1.	Sweat function	↑ Significant improvement, short-term (Schiffmann et al. 2003 [37]) ↑ Non-significant improvement (Bongiorno et al. 2003 [42]) ↑ Significance unknown (Hughes et al. 2011 [48]) — No change (Gupta et al. 2008 [45]; Schiffmann et al. 2007 [52])	↑ Subjective improvement (Eng et al. 2001 [69]; Hilz et al. 2004 [77])
3.5.2.	PNS nerve sensitivity	↓ Significant improvement IENFD (Schiffmann et al. 2006 [38]) ↓ Significant improvement cold/warm threshold (Schiffmann et al. 2003 [37]) — No change in cold/warm sensation (Schiffmann et al. 2006 [38]) — No change in neurological examination score (Jardim et al. 2004 [30]; Jardim et al. 2006 [31])	↓ Significant improvement (Hilz et al. 2004 [77])
3.5.3.	Vestibular/auditory and other CNS outcomes	↑ Significant improvement hearing loss (Hajioff et al. 2003 [28]) ↑ Non-significant improvement peripheral vestibular function (Palla et al. 2003 [34]) — Stabilization of hearing (Sergi et al. 2010 [53]) — No change, neurological examination (Jardim et al. 2004 [30]) ↓ Significant improvement in cerebral blood flow (Moore et al. 2001 [32]) ↓ Improvement of unknown significance in cerebral blood flow (Moore et al. 2002 [33])	— No change, neurological examination (Hilz et al. 2004 [77])
3.5.4.	White matter hyperintensities	No data from clinical or observational studies with approved dose of agalsidase alfa	No data from clinical or observational studies with approved dose of agalsidase beta
3.6.	Pain outcomes	↓ Significant improvement (Schiffmann et al. 2001 [36]; Schiffmann et al. 2003 [37]; Hoffmann et al. 2007 [47]) ↓ Non-significant improvement in acroparesthesias (Jardim et al. 2006 [31]; Bongiorno et al. 2003 [42]) ↓ Unknown significance, improvement (Bongiorno et al. 2003 [42]) — No change (Hughes et al. 2011 [48]; Sergi et al. 2010 [53]; Whitfield et al. 2005 [55])	↓ Significant improvement (Eng et al. 2001 [69]; Hilz et al. 2004 [77]) ↓ Non-significant improvement (Eto et al. 2005 [70]; Mignani et al. 2004 [80])
3.7.	GI outcomes	↓ Unknown significance, improvement (Jardim et al. 2006 [31]; Hoffmann et al. 2007 [46]; Hughes et al. 2011 [48]) ↑ Unknown significance, diarrhea (Hughes et al. 2011 [48];	No data from clinical or observational studies with approved dose of agalsidase beta
3.8.	QoL	↑ Non-significant improvement (Hughes et al. 2011 [48]) — No change, energy levels (Ghali et al. 2012 [100]) — No change, SF-36 (Smid et al. 2011 [54])	↑ Significant improvement (Eto et al. 2005 [70]) ↑ Unknown significance (Eng et al. 2001 [69]; Watt et al. 2010 [83])

Results are summarized as increase (↑), decrease (↓) or no change from baseline to follow-up after ERT initiation. Significance refers to statistical significance. Results are not adjusted for differences in study designs, patient characteristics, or disease stage. Case reports, mixed-ERT publications, and publications with other dose regimens are not included.

CNS, central nervous system; ECG, electrocardiography; eGFR, estimated glomerular filtration rate; EOW, every other week; GI, gastrointestinal; GL-3, globotriaosylceramide; IENFD, intra-epidermal nerve fibre density; LVEF, left ventricular ejection fraction; LVM, left ventricular mass; LVMi, left ventricular mass index; LVWT, left ventricular wall thickness; lyso-GL-3, globotriaosylsphingosine; PNS, peripheral nervous system; pts, patients; QoL, quality of life; SF-36, 36-Item Short Form Health Survey.

### 3.2 GL-3 accumulation

#### 3.2.1 Plasma GL-3

##### 3.2.1.1 Agalsidase alfa 0.2 mg/kg EOW

Treatment with agalsidase alfa was associated with a reduction in plasma GL-3 in four RCT publications [25,27,29,36], of which the last two were placebo-controlled, and four single-arm CT publications [26,35,39,40] (mainly trials with ≤25 patients and follow-up periods of 2.5–54 months) and three OS publications (including 12–22 male patients followed for 12–48 months) [52,101,102]. The change from baseline in plasma GL-3 was statistically significant in one RCT [25], one placebo-controlled RCT with open-label extension [29], one placebo-controlled RCT [36] and its open-label extension [39], one publication from a single-arm CT [35] and two OS publications [52,102]. In both placebo-controlled RCTs, there was a significant decrease in plasma GL-3 in the agalsidase alfa arm compared with no changes in the placebo group [29,36]. There were two publications that reported the impact of agalsidase dose/regimen change [40,52]: in one single-arm CT publication [40], the change from agalsidase alfa 0.2 mg/kg EOW to 0.2 mg/kg weekly resulted in no change in plasma GL-3 compared with pre-switch levels, whereas in another OS publication (12 patients followed for 24–48 months), further reductions, though not statistically significant, in plasma GL-3 were observed when the dose was increased from 0.2 mg/kg EOW to 0.2 or 0.4 mg/kg weekly [52]. In one OS, the four patients with antibodies to agalsidase alfa showed a reduction in plasma GL-3 after 12 months [101] (Supplementary Table 2).

There were three RCT publications in MG populations [27,135,176], one of which reported a significant decrease from baseline in plasma GL-3 with agalsidase alfa 0.2 mg/kg EOW after 12 months [27,176]. One RCT publication reported non-significant reductions from baseline in plasma GL-3 with agalsidase alfa 0.2 mg/kg EOW and 0.2 mg/kg weekly regimens [27]; another RCT publication described no statistically significant differences in plasma GL-3 reductions between for 0.2 mg/kg EOW, 0.1 mg/kg weekly, or 0.2 mg/kg weekly schedules [135].

##### 3.2.1.2 Agalsidase beta 1.0 mg/kg EOW

For agalsidase beta, an overall reduction in plasma GL-3 was reported in four single-arm CT publications (involving 13–21 patients followed for 2.5 to >12 months) [[69], [70], [71], [72]], and five OS publications [75,76,80,101,102]. Agalsidase beta treatment resulted in normalization of plasma GL-3 levels in four publications (including three single-arm CTs [69,70,72] and an OS publication [75]) and statistically significant reductions of plasma GL-3 levels in five publications (three single-arm CTs [[70], [71], [72]] and two OS publications [76,102]). In one single-arm CT, plasma GL-3 levels were above the normal range at baseline, decreased significantly with the 1.0 mg/kg EOW dose to normal levels and remained stable when the dose was reduced to 0.3 mg/kg EOW [72]. In another single-arm CT, switching 15 patients from agalsidase alfa 0.2 mg/kg EOW (mean duration 3.7 years; range 1.6–14 years) to agalsidase beta 1.0 mg/kg EOW for 6 months resulted in a statistically significant further reduction in plasma GL-3 after switch [71]. Two OS publications showed that treatment with agalsidase beta 1.0 mg/kg EOW resulted in larger reductions in plasma GL-3 compared with agalsidase alfa 0.2 mg/kg EOW [100,101], of which one publication was able to demonstrate a statistically significant difference between the two treatment regimens [101]. The same publications also described that a reduction in plasma GL-3 levels can be achieved with a lower dose of agalsidase beta (0.2 mg/kg EOW) [101,102] (Supplementary Table 2). The findings of reductions in plasma GL-3 were supported by three CRs of patients treated with agalsidase beta [91,94,98].

For MG populations, there were nine publications that described the changes in plasma GL-3 with agalsidase beta [79,149,157,160,163,166,168,176,180]. These publications were derived from one RCT [176], one placebo-controlled RCT with open-label extension [149], a longer-term open-label extension study from the same RCT [157] and one study in which patients were treated through an RCT, its open-label extension and then followed further through a registry [163] (58 patients followed for up to 120 months), and five OS publications [79,160,166,168,180]. All these publications noted a general trend towards normalization of plasma GL-3 levels. In the placebo-controlled study, patients receiving agalsidase beta 1.0 mg/kg EOW experienced a decrease in plasma GL-3 while those receiving placebo experienced no change; the difference between groups was statistically significant [149]. One RCT publication reported a statistically significant reduction in plasma GL-3 with agalsidase beta 0.2 mg/kg EOW (in 36 patients followed for ≥12 months) [176]. One OS, in 19 patients followed for 60–126 months, described reduced plasma GL-3 levels after 3 months of agalsidase beta and normalization after 1–2 years [79].

##### 3.2.1.3 Mixed ERT

The findings of reductions in plasma GL-3 were supported by an OS which combined data for agalsidase alfa and agalsidase beta [101], and two mixed-ERT CRs [120,133].

There was one relatively large mixed-ERT MG OS publication (52 patients followed for ≥12 months) which reported a statistically significant decrease in plasma GL-3 for patients treated with agalsidase alfa 0.2 mg/kg EOW or agalsidase beta 0.2 mg/kg EOW, and a non-significant decrease for patients unresponsive to 0.2 mg/kg EOW of either treatment who switched to agalsidase beta 1.0 mg/kg EOW [180]. Decreasing plasma GL-3 levels were also reported in a mixed-ERT OS publication involving 34 renal dialysis or transplant patients followed for 45–48 months [186].

#### 3.2.2 Plasma lyso-GL-3

##### 3.2.2.1 Agalsidase alfa 0.2 mg/kg EOW

Plasma lyso-GL-3 levels decreased significantly with agalsidase alfa in one single-arm CT publication (with data reporting for 21 patients followed for 24 months) [26] and non-significantly in two OS publications (involving 22–29 patients followed for ≥12 months) [101,102]. In one OS publication (with 14 male patients treated with agalsidase alfa for a median of 11 months), a statistically significant increase in plasma lyso-GL-3 levels was reported in patients who switched from agalsidase beta 1.0 mg/kg EOW to agalsidase alfa 0.2 mg/kg EOW [54]. Lastly, plasma lyso-GL-3 was reduced in the two classic patients from an OS publication: one who received agalsidase alfa and one who switched from agalsidase beta 1.0 mg/kg EOW to agalsidase alfa 0.2 mg/kg EOW [110] (Supplementary Table 3).

##### 3.2.2.2 Agalsidase beta 1.0 mg/kg EOW

One single-arm CT publication reported a statistically significant decrease in plasma lyso-GL-3 after switching from agalsidase alfa 0.2 mg/kg EOW (mean 3.7 years, range 1.6–14 years) to agalsidase beta 1.0 mg/kg EOW (in 15 patients followed for 6 months) [71]. Two OS publications (in 22–29 male patients followed for ≥12 months) showed that the decrease in plasma lyso-GL-3 was more notable with agalsidase beta 1.0 mg/kg EOW compared with agalsidase alfa 0.2 mg/kg EOW [101,102]. Agalsidase beta at a reduced dose of 0.2 mg/kg EOW led to a decrease in plasma lyso-GL-3 in two OS publications [101,102], and a statistically significant increase in plasma lyso-GL-3 in another [54] (Supplementary Table 3). One CR also described a decrease in plasma lyso-GL-3 with agalsidase beta [94].

##### 3.2.2.3 Mixed ERT

Statistically significant reductions in plasma lyso-GL-3 with mixed-ERT were reported in one CT publication [182], four OS publications [51,101,102,109] and one CR [133].

#### 3.2.3 Urinary GL-3

##### 3.2.3.1 Agalsidase alfa 0.2 mg/kg EOW

One placebo-controlled RCT [36], one placebo-controlled RCT with open-label extension [29] and three single-arm CTs [26,35 and 39, where 39 is an extension study of 36] reported a decrease in urinary GL-3 levels with agalsidase alfa (including 12–21 male patients followed for 6–54 months). In one of the placebo-controlled RCTs, urinary GL-3 declined significantly compared with the placebo group [29], and in the other, urinary GL-3 levels decreased with agalsidase alfa but increased in the placebo group [36]. One single-arm CT publication (including 12 patients followed for ≤120 months) in which patients were switched from agalsidase alfa 0.2 mg/kg EOW to the same dose weekly reported no change in urinary GL-3 [40] and another small single-arm CT reported an increase in urinary GL-3 upon switching from agalsidase beta 1.0 mg/kg EOW to agalsidase alfa 0.2 mg/kg EOW [26]. There were two OS publications (including 6–12 patients followed for 12–48 months) [52,55], one of which noted further reductions in urinary GL-3 levels when the regimen was changed from EOW to a weekly schedule [52]. Urinary GL-3 levels increased in antibody-positive patients in one OS publication (including 29 patients followed for >12 months) [101] (Supplementary Table 4).

There were two RCT publications which reported urinary GL-3 levels in MG populations: one reported an overall decrease [176], and the other a trend towards decline, with agalsidase alfa 0.2 mg/kg weekly [135]. In an OS publication, a decrease in urinary GL-3 in antibody-negative patients, and an increase in urinary GL-3 in antibody-positive patients was reported following treatment with agalsidase alfa 0.2 mg/kg EOW (52 patients followed for ≥12 months) [180].

##### 3.2.3.2 Agalsidase beta 1.0 mg/kg EOW

For agalsidase beta, three single-arm CT publications (including 13–21 patients followed for 6–18 months) showed a decrease in urinary GL-3 levels [[70], [71], [72]]. During one single-arm trial, urinary GL-3 levels decreased significantly with agalsidase beta 1.0 mg/kg EOW and then increased significantly when the agalsidase beta dose was reduced [72]. In a separate single-arm CT, there was a non-significant decrease in urinary GL-3 levels after switching from agalsidase alfa 0.2 mg/kg EOW to agalsidase beta 1.0 mg/kg EOW [71]. In one OS publication, antibody-positive patients showed a decrease in urinary GL-3 levels with both agalsidase beta 1.0 mg/kg EOW and 0.2 mg/kg EOW, whereas, with agalsidase alfa 0.2 mg/kg EOW, an increase was seen [101]. In another OS publication (in three patients), urinary GL-3 remained normal throughout 18 months of follow-up [80]. In a CR, urinary GL-3 decreased after 12 months [99] (Supplementary Table 4).

In MG populations, one RCT publication reported a non-significant decrease in urinary GL-3 with agalsidase beta 0.2 mg/kg EOW [176]. In two OS publications, there was a statistically significant decrease in urinary GL-3 (11 patients followed for ≤27 months) [160]; and 52 patients followed for ≥12 months [180]). In one OS publication in 19 patients followed for 60–126 months, urinary GL-3 levels declined after 3 months and normalized within 3 years [79]. One OS publication reported a decrease in urinary GL-3 with agalsidase beta 1.0 mg/kg EOW in both antibody-negative and antibody-positive patients [180]; with the agalsidase beta 0.2 mg/kg dose, there was a decline in urinary GL-3 in antibody-negative patients and an increase in antibody-positive patients [180].

##### 3.2.3.3 Mixed ERT

One mixed-ERT study publication in males showed reductions in urinary GL-3 [101]. For MG publications, urinary GL-3 levels were undetectable both at baseline and at endpoint in one OS publication (in 34 renal dialysis and transplant patients followed for 45–48 months) [186]. A decrease in urinary GL-3 in antibody-negative patients, and an increase in urinary GL-3 in antibody-positive patients were reported in an OS involving 18 patients followed for 6–12 months [178].

#### 3.2.4 Urinary lyso-GL-3

##### 3.2.4.1 Agalsidase alfa/beta

There were no publications reporting on the change in urinary lyso-GL-3 following treatment with agalsidase alfa or agalsidase beta.

##### 3.2.4.2 Mixed ERT

Urinary lyso-GL-3 levels decreased during treatment with mixed-ERT in one male patient followed for 30 months in the only publication that reported this outcome [106].

#### 3.2.5 GL-3 accumulation: histology

##### 3.2.5.1 Renal cells

###### 3.2.5.1.1 Agalsidase alfa 0.2 mg/kg EOW

In one placebo-controlled RCT publication, GL-3 in kidney biopsy samples was reduced from baseline in both the agalsidase alfa and placebo groups [36] (Supplementary Table 5).

###### 3.2.5.1.2 Agalsidase beta 1.0 mg/kg EOW

Two single-arm CT publications reported change in GL-3 levels across various renal tissues [72,73]. One publication observed statistically significant increases in the proportions of patients experiencing GL-3 clearance in six of eight different renal cell types after 6 months of treatment. Clearance levels were generally maintained when patients received the 0.3 mg/kg EOW dose during 18 months of treatment in a single-arm CT [72]. A 73% decline in podocyte GL-3 content and a 63% reduction in podocyte volume after 11–12 months of agalsidase beta was reported in the other single-arm CT publication [73]. In the same publication, there was a reduction in GL-3 score (in three of six patients) and a statistically significant increase in the number of podocytes/biopsy without visible GL-3 inclusions with agalsidase beta treatment [73]. Also, one single-arm CT showed that the accumulation of GL-3 in kidney cells was reduced with different dosing regimens in four of five patients after five infusions (2.5 months) [69] (Supplementary Table 5). In one CR, agalsidase beta decreased renal GL-3 deposits (histological analysis based on specific staining for GL-3 in renal biopsy samples) [89]. In another CR, the composite podocyte score was reduced with agalsidase beta but increased after switching to agalsidase alfa [130].

Four MG publications reported GL-3 clearance in renal capillary and non-capillary endothelial cells with agalsidase beta: the placebo-controlled RCT [149], its secondary analysis [153], and its extension study [150], and the OS [160]. In the placebo-controlled RCT, the proportion of patients achieving GL-3 clearance from renal capillary endothelial cells was significantly higher with agalsidase beta than with placebo [149]. A secondary analysis of the same study reported that, compared with placebo, significantly more patients treated with agalsidase beta achieved GL-3 clearance from glomerular capillary endothelial cells, arterial/arteriolar endothelial cells, mesangial cells, and interstitial cells [153]. The difference in proportion of patients achieving clearance was statistically significant compared with baseline in the open-label extension study of the RCT (up to 54 months’ follow-up) [150] and one small OS (in 11 patients followed for ≤27 months) [160].

###### 3.2.5.1.3 Mixed ERT

One CR noted that the adult male patient who started ERT at 18 years of age showed clearance of GL-3 deposits from podocytes, yet the paediatric male patient who started ERT at 7 years of age showed better clearance [130].

##### 3.2.5.2 Cardiac cells

###### 3.2.5.2.1 Agalsidase alfa 0.2 mg/kg EOW

In one placebo-controlled RCT with open-label extension, during the 6-month RCT phase the six patients who were treated with agalsidase alfa showed a non-significant reduction in GL-3 accumulation in myocardial cells while the eight patients in the placebo group experienced an increase [29] (Supplementary Table 6). A MG OS publication reported GL-3 accumulation in cardiomyocytes with agalsidase alfa (17 patients followed for ≤40 months), but data for both pre- and post-ERT were not available [143].

###### 3.2.5.2.2 Agalsidase beta 1.0 mg/kg EOW

There was one single-arm CT publication in male patients which reported a non-significant decrease in the capillary endothelium GL-3 scores [69] (Supplementary Table 6).

In MG publications of agalsidase beta, a reduction in GL-3 accumulation in cardiac endothelial cells was reported in three publications: one was a placebo-controlled RCT with open-label extension [149], and two were longer-term open-label extensions of the same study (including 58 patients followed for up to 54 months) [150,155]. In the RCT, patients treated with agalsidase beta 1.0 mg/kg EOW experienced a decrease in cardiac GL-3 deposits, while those in the placebo group, experienced a small increase [149].

###### 3.2.5.2.3 Mixed ERT

There were no publications from either male or MG publications describing the effect of mixed-ERT regimens on cardiac GL-3 levels.

##### 3.2.5.3 Other cell types

###### 3.2.5.3.1 Agalsidase alfa 0.2 mg/kg EOW

There were no publications describing change from baseline following agalsidase alfa treatment in GL-3 levels in other cell types.

###### 3.2.5.3.2 Agalsidase beta 1.0 mg/kg EOW

One single-arm CT publication (in 15 patients followed for ≤ 2.5 months) reported a decrease in GL-3 levels in liver cells in each of the five dosing regimens [69]. In another, skin GL-3 was significantly reduced in 21 patients followed for 6 months [72]. One single-arm study found that 100% of patients had clearance of GL-3 in interstitial capillary endothelial cells with agalsidase beta 1.0 mg/kg EOW but 90% with the reduced dose of agalsidase beta 0.3 mg/kg [72]. In one OS publication, treatment achieved GL-3 clearance from skin (as measured by an increase in the number of patients with undetectable/trace levels of skin GL-3 following treatment) [75] (Supplementary Table 7). CRs described clearance of glycolipid storage deposits from sweat glands [97] and vascular endothelial cells [93].

In MG publications, one placebo-controlled RCT followed by open-label extension (in 58 patients) reported a statistically significant reduction in skin epithelial GL-3 scores compared with placebo after 5 months, and during the open-label extension (after 6–12 months follow-up), 96% of patients had total clearance of skin epithelial GL-3 [149]. These low levels were sustained in three further open-label extensions of the same study (after 30-36 months [154,157] and after 54 months [150]).

###### 3.2.5.3.3 Mixed ERT

There were no publications for mixed-ERT regimens describing other organ GL-3 levels.

### 3.3 Renal outcomes

#### 3.3.1 Estimated glomerular filtration rate (eGFR)

##### 3.3.1.1 Agalsidase alfa 0.2 mg/kg EOW

For agalsidase alfa, eGFR was reported as a measure of kidney function during ERT in six CT publications (including one placebo-controlled RCT with open-label extension [29], one placebo-controlled RCT [36] and its two extension studies [38,39], and two single-arm studies [35,40]) and seven OS publications [41,43,44,48,49,52,54]. In the placebo-controlled RCT, renal function declined significantly more in the placebo group than in the agalsidase alfa group [36]. In one placebo-controlled RCT with open-label extension, eGFR was significantly increased compared with baseline in the seven patients receiving agalsidase alfa, while the patients receiving placebo experienced no changes [29]. Another single-arm CT publication (in 12 patients) noted an increase in eGFR after 6 months but this was not statistically significant [35]. The decline in eGFR appeared to be slowed compared with placebo or untreated patients when the 14 agalsidase alfa-treated patients from the placebo-controlled RCT [36] were followed for 48–54 months [39], in a large OS (in 360 patients, of whom 115 male patients had eGFR data, followed for 60 months) [41], and in a small OS (in 12 patients followed for 24–48 months) [52]. Other publications showed decreases in eGFR, including one open-label extension of a placebo-controlled RCT (in 26 patients followed for 12–18 months) [38], three publications from the same registry (including 115–172 patients followed for 36–134 months) [43,44,48], and one OS (in 15 patients treated for a median of 11 months with agalsidase alfa [54]). Another single-arm CT publication reported a slowed decline in eGFR on switching from agalsidase alfa 0.2 mg/kg EOW to the same dose weekly (in 12 patients followed for <120 months) [40]. One OS publication reported stabilized eGFR in 21 male patients after 10 years [49]. Three CRs suggested that agalsidase alfa might slow the decrease in eGFR [60,62,67] (Table 2).

**Table 2:** Glomerular filtration rate outcomes with approved doses of agalsidase alfa and agalsidase beta in adult male patients

	Study, year [reference] (number of patients^a^) *Evidence grade*^b^	Male, n (%)^c^	Duration (months)	Units	Baseline (number of patients^d^)	End-point (number of patients^e^)	Overall result (p value/ 95% CI)
Alfa	Beck et al. 2015 [41] (N=677) *Grade 3*	360 (53)	60	mL/min/1.73 m^2^ (mean annualized eGFR slope ± SE)	eGFR at BL ≥60: NR (n=117) eGFR at BL <60: NR (n=18) Proteinuria at BL ≥1.0 g/24 h: Mean: 74.4 (n=16) 0.1–1.0 g/24 h: Mean: 101.8 (n=74) <0.1 g/24 h: Mean: 105 (n=15)	eGFR at BL ≥60: −1.68 ± 0.19 (n=117) eGFR at BL <60: −2.86 ± 0.53 (n=18) Proteinuria at BL ≥1.0 g/24 h: −4.76 ± 0.56 (n=16) 0.1–1.0 g/24 h: −1.62 ± 0.23 (n=74) <0.1 g/24 h: −1.32 ± 0.48 (n=15)	↓ (95% CI −2.05, −1.31) ↓ (95% CI −3.90, −1.83) ↓ (95% CI −5.85, −3.66) ↓ (95% CI −2.08, −1.17) ↓ (95% CI −2.26, −0.38)
	Feriozzi et al. 2009 [43] (N=165) *Grade 3*	115 (70)	36	mL/min/1.73 m^2^	94.5 [30.8] (n=115)	81.2 [32.5] (n=115)	↓ (p<0.01)
	Feriozzi et al. 2012 [44] (N=208) *Grade 3*	134 (64)	60–134	mL/min/1.73 m^2^	97.3 [26.5] (n=134)	79.7 [31.8] (n=134)	↓ (p<0.01)
	Hughes et al. 2008 [29] (N=7) *Grade 1a*	15 (100)	6	mL/min/1.73 m^2^	106.3 ± 13.9 (n=7)	Increase within normal range: 106–131 mL/min	↑ (p=0.007)
	Hughes et al. 2011 [48] (N=250) *Grade 3*	172 (69)	≥48	mL/min/1.73 m^2^ (median [10−90th percentile])	88.2 (41.1–137.0) (n=105)	80.3 (34.2–119.7) (n=105)	↓ (p<0.001)
	Kampmann et al. 2015 [49] (N=45) *Grade 3*	21 (47)	Median (range): 130 (115–150)	mL/min/1.73 m^2^	≥90 mL/min/1.73 m^2^: Values: NR <90 mL/min/1.73 m^2^: Values: NR	≥90 mL/min/1.73 m^2^: 10 years: NC <90 mL/min/1.73 m^2^: 10 years: NC	NC (NS) NC (NS)
	Pastores et al. 2007 [35] (N=22) *Grade 1c*	20 (91)	Range: 2.5–15	mL/min/1.73 m^2^	Transplant pts: 64.5 ± 6 (n=12)	Transplant pts: 71.6 ± 8.0 (n=12)	↑ (p=0.07)
	Schiffmann et al. 2001 [36] (N=14) *Grade 1a*	14 (100)	5.5	Creatinine clearance mL/min/1.73 m^2^	92.7 ± 6.2 (n=13)	94.8 ± 7.7 (n=13)	↑ (NR)
	Schiffmann et al. 2006 [38] (N=26) *Grade 1c*	26 (100)	18	mL/min/1.73 m^2^	97 (n=26)	90 (n=25)	↓ (p=0.38)
	Schiffmann et al. 2006 [39] (N=25) *Grade 1c*	25 (100)	48–54	mL/min/1.73 m^2^	88.4 [26.0] (n=24)	Month 48: 75.1 [32.7] (n=24)	↓ (p=0.039)
	Schiffmann et al. 2007 [52] (N=12) *Grade 2*	12 (100)	24–48	mL/min/ 1.73 m^2^	77.8 [30.4] (n=12)	53.7 [21.0] Mean change: −8.0 [2.8]/year	↓ (NR)
Beta	Eto et al. 2005 [70] (N=13) *Grade 1c*	13 (100)	4.6	Creatinine clearance mL/min	126.6 [41.8] (n=13)	115.3 [30.4] (n=13)	↓ (p=0.216)
	Kim et al. 2016 [79] (N=19) *Grade 2*	15 (79) *11 adults,* *4 paediatric pts*	60–126	mL/min/1.73 m^2^	107.3 [28.5] (n=11) During shortage: 78.3 [29.4] (n=7)	−3.8 [4.5]/year (n=11) Stratified by BL proteinuria <0.1g/day: −0.7 [3.8]/year (n=3) >0.1 g/day: −6.3 [4.2]/year (n=6) During shortage: 63.3 [37.2] (n=7)	↓ (NR) NC (NS) NC (NS) NC (p>0.05)
	Lubanda et al. 2009 [72] (N=21) *Grade 1c*	21 (100)	5.5	mL/min/1.73m^2^	Median: 92.5 (n=21)	Median: 93.1 (n=21)	NC (NS)
	Najafian et al. 2016 [73] (N=6) *Grade 1c*	6 (100)	11–12	mL/min/1.73m^2^	Range: 111–154 (n=6)	Range: 114–190 (n=5)	NC/↓/↑ (NR)
	Warnock et al. 2012 [82] (N=213) *Grade 3*	151 (71)	>24	mL/min/1.73 m^2^/year	Q1^f^: 99 [22.6] (n=37) Q2: 77 [32.9] (n=37) Q3: 88 [38.7] (n=38) Q4: 2 [34.3] (n=36)	Q1: −0.1 [1.20] (n=38) Q2: −2.1 [0.52] (n=38) Q3: −3.8 [0.61] (n=38) Q4: −6.7 [2.26] (n=37)	↓ (NR)

Data are means (SD) or means ± SE or medians (range), unless otherwise indicated.

Case series, case reports, mixed-ERT publications, paediatric-adult-mixed publications, and publications with other dose regimens are not included.

↓, decrease; ↑, increase; BL, baseline; CI, confidence interval; ERT, enzyme replacement therapy; eGFR, estimated glomerular filtration rate; h, hours; NC, no change; NR, not reported; NS, not significant; pt, patient; Q1, first quartile (based on progression of renal disease during follow-up); Q2, second quartile; Q3, third quartile; Q4, fourth quartile; SD, standard deviation; SE, standard error.

a Total number of patients included in the study who were treated with ERT;

b Study grades defined as follows: Grade 1a randomized controlled trial; Grade 1c single-arm clinical trial; Grade 1a/c randomized controlled trial with single-arm open-label extension; Grade 2 prospective observational study; Grade 3 retrospective observational study; Grade 4 case series; Grade 5 case report;

c Number of male patients who were treated with ERT;

d Number of male, ERT-treated patients with data for the outcome at baseline;

e Number of male, ERT-treated patients with data for the outcome at endpoint;

f eGFR slope Quartile 1: −1.2 to 15.3, Quartile 2: −2.8 to -1.3, Quartile 3: −4.8 to −2.9, Quartile 4: −15.5 to −4.9.

In MG populations, eGFR did not change significantly with agalsidase alfa. This was reported in 12 publications (five CT publications including three RCTs [27,135,176] and two single-arm studies [26,35], five OS publications [139,141,142,146,147] and two registry studies [137,144]). The five CT publications included three pure agalsidase alfa publications (one RCT [27] and two single-arm studies [35,136] in 8–44 patients followed for ≤24 months), and two mixed-ERT publications in which the agalsidase alfa arm data were reported separately (one single-arm CT publication in which 100 patients were followed for 24 months [26] and one RCT where 36 patients were followed for ≥12 months [176]). The five OS publications included a large report (234 patients) and one long-term follow-up (57 months) [139,141,142,146,147]. Of the registry studies, one followed 181 patients for 60 months and the other followed 314 patients for an average of 17 months [137,144].

The effect of switching from agalsidase beta 1.0 mg/kg EOW to agalsidase alfa 0.2 mg/kg EOW differed across four publications [26,177,179,181]. Two publications (one single-arm CT including 40 male switch patients [26] and one OS including 7 male switch patients [179]) described no (clinical) change in eGFR 24 months after switching. One OS showed that eGFR was reduced in 37 patients, of whom 20 were male, 12 months after switching [181], and was statistically significant decreased at 24 months after the switch [177].

##### 3.3.1.2 Agalsidase beta 1.0 mg/kg EOW

Publications from three single-arm CT publications [70,72,73] and two OS publications [79,82] reported on the effect of agalsidase beta on eGFR. One single-arm CT (in 13 patients followed for 5 months) reported a non-significant decrease in eGFR [70], whereas another showed no change in eGFR during agalsidase beta 1.0 mg/kg EOW or after a dose reduction to 0.3 mg/kg EOW (in 21 patients who received normal dosing for 6 months and reduced dosing for 18 months) [72]. In one single-arm CT publication that reported patient-level change in eGFR, eGFR decreased in two of six patients, no change was observed in another two of six, and an increase was seen in one of six patients (one patient was not evaluable at follow-up) [73]. One large OS publication reported that early agalsidase beta treatment initiation was associated with a reduced slope of eGFR decline (in 151 patients followed for 24 months) [82] and another OS (11 patients followed for 60–126 months) reported a significantly lower eGFR decline in patients with lower proteinuria before ERT [79]. In this study, temporary agalsidase beta dose reduction resulted in a statistically significant decrease in eGFR (compared with full-dose period, seven patients) [79] (Table 2). One CR reported a decline in eGFR during 16 years of agalsidase beta treatment [84].

In MG populations, the observation that agalsidase beta had a stabilizing effect on eGFR was consistently reported in 12 publications (one RCT with open-label extension [149] and two further open-label extensions in the same patients [150,157] and one single-arm CT publication [152] and eight OS publications [159,163,165,175,177,180,181,188]). Two publications reported different effects of agalsidase beta dose reduction on eGFR: one OS publication reported a statistically significant decrease (105 patients followed for 8–16 months) [181], whereas an RCT observed no effect (52 patients followed for 12 months or longer) [176].

##### 3.3.1.3 Mixed ERT

Eight mixed-ERT OS publications (including some large, long-term studies including ≤100 patients followed for ≤120 months) agreed that ERT might slow the decrease in eGFR [51,103,104,107,109,112,114,116]. One large, long-term OS publication reported that patients without proteinuria responded better to treatment than patients with overt proteinuria (109 patients followed for ≤116 months) [103]. Four CRs noted that eGFR remained stable or decreased during mixed-ERT [125,[130], [131], [132]].

In MG populations, one OS publication reported a statistically significant decline in eGFR following 80 months of mixed ERT (26 patients) [108], and another OS noted no statistically significant change in eGFR for patients treated with agalsidase alfa 0.2 mg/kg EOW or agalsidase beta 0.2 mg/kg EOW combined [180].

#### 3.3.2 Albuminuria/proteinuria

##### 3.3.2.1 Agalsidase alfa 0.2 mg/kg EOW

Agalsidase alfa was generally reported to have no effect on proteinuria. These findings were reported by two CT publications (first a randomized, placebo-controlled portion in which 14 patients were followed for 6 months [36], and then an open-label extension of 48–54 months in the same patients [39]) and six OS publications [[42], [43], [44],^48^,^49^,^52^], including three large registry studies involving 77–115 patients with proteinuria data followed for 36–89 months [43,44,48]. One OS publication reporting the impact of 10 years of agalsidase alfa treatment noted no changes in patients without baseline proteinuria but a statistically significant increase in proteinuria in those with pre-existing proteinuria at baseline [49]. The only statistically significant finding in the registry studies was an increase in proteinuria in patients with chronic kidney disease stage 1 at baseline [44]. When the agalsidase alfa regimen was changed from 0.2 mg/kg EOW to 0.2 mg/kg weekly, there was a decrease in proteinuria (OS publication [52]) (Supplementary Table 8).

Effects of agalsidase alfa on proteinuria were very variable across five different CRs: two CRs showed improved proteinuria [56,62], two described worsening [65,125], and one noted no change during treatment [64]. In two CRs, the change in proteinuria was dependent on agalsidase alfa dose: patients experienced an increase in proteinuria with agalsidase alfa 0.2 mg/kg EOW dose but a decrease in proteinuria when the dose was subsequently increased to 0.4 mg/kg EOW [63,67].

For MG populations, one RCT [176], one single-arm CT publication [136] and three OS publications [139,142,147] reported no statistically significant change in proteinuria with agalsidase alfa. Two OS publications reported the effect of switching from agalsidase beta 1.0 mg/kg EOW to agalsidase alfa 0.2 mg/kg EOW on proteinuria/albuminuria levels [179,181]: in one, there was a statistically significant increase in albuminuria following the switch [181], and in the other, there was no change in proteinuria [179].

##### 3.3.2.2 Agalsidase beta 1.0 mg/kg EOW

There were two single-arm CT publications [72,73] and two OS publications [79,80] which reported proteinuria/albuminuria outcomes following agalsidase beta. One single-arm CT publication reported no statistically significant changes with agalsidase beta 1.0 mg/kg EOW or following a dose reduction to 0.3 mg/kg EOW in 21 patients followed for ≤18 months [72]. A resolution of proteinuria was observed in all patients with follow-up data available in a single-arm CT publication of six patients followed for 12 months [73]. In an OS publication in 11 patients followed for 60–126 months, there was no statistically significant effect of agalsidase beta treatment on proteinuria, and no impact of baseline proteinuria on treatment outcomes [79]. Another OS publication reported that one of three patients had detectable proteinuria at baseline which resolved during treatment [80] (Supplementary Table 8). Five CRs with agalsidase beta showed an improvement in proteinuria [86,88,92,95,96], whereas one showed no change [87], and one a worsening [85].

One MG single-arm CT reported a decrease in proteinuria with agalsidase beta when used in conjunction with antiproteinuric therapy (angiotensin-converting enzyme inhibitor [ACEi] and/or angiotensin receptor blocker [ARB]) [152]. Two OS publications [164,175] showed a statistically significant reduction in proteinuria in 40 patients followed for ≥60 months and a resolution of proteinuria in some patients (in a small study of 11 patients followed for 29–58 months). One OS publication reported a statistically significant decrease in proteinuria with agalsidase beta in combination with paricalcitol [188]. One open-label extension of a placebo-controlled RCT [157] and four OS publications [159,160,165,181] reported no statistically significant change in proteinuria or albuminuria. In an RCT [176] and another OS publication [181], agalsidase beta therapy at a dose of 0.2 mg/kg EOW or with a dose reduction to 0.3–0.5 mg/kg EOW did not result in statistically significant changes in proteinuria and albuminuria.

##### 3.3.2.3 Mixed ERT

The effects of mixed ERT on proteinuria/albuminuria are supported by four mixed-ERT OS publications, including one study investigating proteinuria in 109 patients after 116 months [103] and three smaller studies reporting changes in albuminuria [51,107,109]. In one CR, the patient had slightly elevated albuminuria levels at baseline, which decreased after 1 year of agalsidase beta 1.0 mg/kg EOW and remained stable for the next 10 years (with different ERT regimens) [130]. Proteinuria was also described in another two CRs [121,125].

In MG populations, there was one mixed-ERT OS publication including renal dialysis and transplant patients that reported a non-significant increase in proteinuria in transplanted patients [186].

### 3.4 Cardiac outcomes

#### 3.4.1 Left ventricular mass (LVM)/left ventricular mass index (LVMi)

##### 3.4.1.1 Agalsidase alfa 0.2 mg/kg EOW

Two RCT publications [27 and the placebo-controlled 29] and four OS publications [41,48,49,189], reported measures of LVM with agalsidase alfa therapy. In a small placebo-controlled RCT with open-label extension in six patients using magnetic resonance imaging (MRI) assessment of left ventricular mass index (LVMi) (followed for 30 months), agalsidase alfa showed a significant reduction in LVM compared with placebo [29]. In one RCT that included 26 patients followed for 12 months, a non-significant increase in LVM was seen with agalsidase alfa 0.2 mg/kg EOW, whereas no change was seen with agalsidase alfa 0.2 mg/kg weekly and a non-significant decrease with agalsidase alfa 0.4 mg/kg weekly [27]. In one registry study publication (in 172 patients followed for ≥48 months, 45 of whom had had LVM data available), LVM decreased significantly in the 22 patients with baseline LVH [48], and in another OS publication (in 360 patients followed for 60 months, 71 of whom had LVMi data available), the progression of LVH was slowed compared with untreated patients, but still increased significantly [41]. After 10 years of agalsidase alfa there was no change in LVMi in patients without baseline LVH, but a statistically significant decrease in LVMi in patients with baseline LVH (OS in 21 patients) [49]. In the final small OS (four patients), switching from agalsidase beta 1.0 mg/kg EOW to agalsidase alfa 0.2 mg/kg EOW led to a small decrease in LVM [189] (Supplementary Table 9).

There were four CRs which reported an increase in LVH/LVMi with agalsidase alfa after 12 months [61], 6 years [64], 9 years [57], and 12 years [60]. Another CR described stabilization of LVMi, indicated by absence of progression to LVH, in one patient treated with agalsidase alfa [59] and in another who switched regimens (from agalsidase alfa to agalsidase beta and back to agalsidase alfa) [84]. Normalization of hypertrophy after 6 years of treatment [68], and fluctuations in LVM depending on dose, with a lower dose causing an increase in LVM and a higher dose a decrease [63], were also found in CRs.

Two RCTs [27,176] and one single-arm CT publications [26] in MG populations showed that LVM parameters remained stable. LVM was reported to decrease in two registry publications: one in 314 patients followed for 17 months [137] and the other in 181 patients followed for 60 months [144]. In one OS publication, the impact of agalsidase alfa on LVMi was variable [143], while another reported no change in LVMi when patients were switched from agalsidase beta 1.0 mg/kg to agalsidase alfa 0.2 mg/kg EOW [179].

##### 3.4.1.2 Agalsidase beta 1.0 mg/kg EOW

For agalsidase beta, five OS publications reported LVH outcomes [7,76,[79], [80], [81]]. LVMi decreased significantly in two large registry study publications [7,81]; LVM decreased in 115 patients followed for 59 months which was a statistically significant decrease in patients who started agalsidase beta treatment at age <30 years; LVM increased, however, in patients who started ERT at age ≥50 years [7]. LVM did not change in the two smaller study publications (five to eleven patients followed for 10–126 months) [76,79] (Supplementary Table 9). In four CRs LVMi declined or stabilized with agalsidase beta [84,87,90,91].

LVM was reported in one MG RCT [176], one MG single-arm CT publication [151] and 14 MG OS publications [[158], [159], [160], [161],[164], [165], [166], [167],^169^,^170^,^173^,^174^,^179^,^180^], half of which reported a statistically significant reduction in LVM with agalsidase beta. One of these noted a statistically significant decrease from baseline in LVM with ERT in patients without fibrosis, but no such effect in patients with mild or severe fibrosis [174].

##### 3.4.1.3 Mixed ERT

Findings from mixed-ERT studies were reported across nine OS publications [101,104,107,109,110,112,113,114,116] including a large study in 100 patients followed for 59–64 months [114]. One OS publication showed that patients with an abnormal baseline electrocardiogram (ECG) had a statistically significant increase in LVMi, while patients with normal ECG at baseline did not experience any statistically significant change in LVMi (neither progression nor regression) with mixed-ERT treatment (6 years’ follow-up) [113]. One CR noted a deterioration in LVMi [125] but three other CRs showed a decline or stabilization in LVMi [122,124,127].

LVMi with mixed ERT was also described in one MG RCT [176], another single-arm CT publication [26], and eight OS publications [51,103,179,180,182,183,185,186]. In one of these OS publications (25 patients), there was a statistically significant decrease in LVM and LVMi after 12 months [182]. In another mixed-ERT OS publication including 40 patients, LVM decreased in the first few years after treatment initiation and increased thereafter (but neither change was statistically significant) [183].

#### 3.4.2 Left ventricular wall thickness (LVWT)

##### 3.4.2.1 Agalsidase alfa 0.2 mg/kg EOW

One OS including 21 males with mild hypertrophy, reported a statistically significant decrease in LVWT after 10 years of treatment with agalsidase alfa [49], while two CRs noted an increase in LVWT [57,64]. One OS reported a small decrease in LVWT after switching from agalsidase beta 1.0 mg/kg to agalsidase alfa 0.2 mg/kg EOW [189] (Supplementary Table 10).

In one large registry publication of a MG population of 314 patients, there was decreased LVWT after 12 and 24 months of agalsidase alfa [137]. In two OS publications, wall thickness measures did not change on switching from agalsidase beta 1.0 mg/kg to agalsidase alfa 0.2 mg/kg EOW (LVWT [179]; septal and posterior wall thickness [181]).

##### 3.4.2.2 Agalsidase beta 1.0 mg/kg EOW

Wall thickness outcomes with agalsidase beta were assessed in male patients in three OS publications. One study showed a statistically significant decrease in maximal wall thickness (MWT) and left ventricular end-diastolic diameter (LVEDD) in 44 patients followed for a median of 36 months [81]. Another OS in 10 patients followed ≤12 months reported no change in LVWT parameters (MWT, LVEDD, and left ventricular end systolic diameter) [76], whereas a third OS in three patients demonstrated that wall thickness decreased in two patients and increased in one patient after 18 months [80]. LVWT stabilized or decreased in two CRs [87,91] (Supplementary Table 10).

For MG populations, agalsidase beta reduced LVWT parameters in six publications (one single-arm CT [156] and seven OS publications [159,164,167,170,173,174,179]). Agalsidase beta did not change LVWT in four OS publications [163,166,171,181], including three large studies of 56–105 patients followed for ≤58 months [163,171,181]. One of these noted that LVWT was stable in those who started therapy at age <40 years, but was significantly higher if patients received their first dose of ERT when aged ≥40 years [163]. In one OS, agalsidase beta dose reduction did not have a statistically significant effect on posterior LVWT (median of 12 months’ follow-up) [181].

##### 3.4.2.3 Mixed ERT

Two mixed-ERT OS publications [109,113] and two CRs [122,126] indicated that LVWT parameters stabilized or decreased in male patients.

In MG populations, there was one OS that demonstrated a statistically significant decrease in intraventricular septum thickness [IVST] (25 patients treated for 12 months) [182], but three longer-term OS publications (37 to 64 months) reported no change in LVWT [184,185,187]. One mixed-ERT OS publication involving a MG population of 34 patients followed for 45–48 months reported an increased posterior LVWT in chronic haemodialysis patients and a decreased posterior LVWT in renal transplant patients [186].

#### 3.4.3 Left ventricular ejection fraction (LVEF)

##### 3.4.3.1 Agalsidase alfa 0.2 mg/kg EOW

One placebo-controlled RCT publication with open-label extension reported no change in LVEF following 6 months of agalsidase alfa (seven patients) [29]. An OS including 21 males reported no statistically significant change in LVEF after 10 years of treatment with agalsidase alfa [49] (Supplementary Table 11). One CR showed that the LVEF remained normal after 5 years of treatment with agalsidase alfa [57].

Two OS publications of MG populations reported no change in LVEF when patients were switched from agalsidase beta 1.0 mg/kg to agalsidase alfa 0.2 mg/kg EOW (105 patients followed for 8–16 months [181] and ten patients followed for 20 months [179]).

##### 3.4.3.2 Agalsidase beta 1.0 mg/kg EOW

For agalsidase beta, one registry OS publication reported a statistically significant improvement in ejection fraction in 44 patients followed for a median of 36 months [81]. One OS [80] and two CRs [86,88] also described an improvement, but other CRs described stabilization [85] or worsening in LVEF [90] (Supplementary Table 11).

In MG publications, there was no change in LVEF with agalsidase beta in two single-arm CT publications [151,156] and nine OS publications [164,[166], [167], [168],^170^,^171^,^173^,^175^,^179^] that included two large studies (40 patients followed for ≥60 months [175]; and 56 patients followed for ≤58 months [171]). One MG OS publication did report a small but statistically significant increase in ejection fraction [161]. Dose reduction (to 0.3–0.5 mg/kg EOW) was reported not to lead to a change in LVEF in the large OS publication (in 105 patients followed for 8–16 months) [181].

##### 3.4.3.3 Mixed ERT

One mixed-ERT OS reported no change in ejection fraction in 26 male patients [113], whereas one CR showed an increase [122].

In MG populations, the effect on LVEF was described in three OS publications [177,183,185]. Only one of these studies (the large, long-term study of 40 patients followed for 20–71 months) reported a decrease in ejection fraction in patients during the early years of ERT, with no change in ejection fraction later in treatment [183].

#### 3.4.4 Electrocardiogram (ECG) measures

##### 3.4.4.1 Agalsidase alfa 0.2 mg/kg EOW

One placebo-controlled RCT publication in 14 patients followed for 6 months [36] and another placebo-controlled RCT (6 months) with open-label extension (24 months) in seven patients [29] reported a decrease in QRS duration with agalsidase alfa. In a retrospective analysis including 21 males, one patient developed conduction abnormalities during ERT [49] (Supplementary Table 12). One CR described occurrence of persistent atrial fibrillation [59], another noted an increase of QRS voltages with deeply negative T waves after 9 years of treatment [57] and a third showed ST-segment depression and negative T waves [64]. There were no publications describing changes in ECG measures with agalsidase alfa from MG publications.

##### 3.4.4.2 Agalsidase beta 1.0 mg/kg EOW

For agalsidase beta, one single-arm CT publication reported no change in ECG measures in three patients treated with agalsidase beta 1.0 mg/kg EOW and 12 patients treated with different agalsidase beta dosing regimens, all followed for 2.5 months (reported as a safety parameter in this study) [69]. One large registry OS publication (in 44 patients followed for a median of 36 months) reported a statistically significant increase in P wave duration and PQ interval and a statistically significant decrease in heart rate-corrected QT interval [81]. Another OS of three patients followed for 18 months reported that ECG measures did not change [80] (Supplementary Table 12). In a CR, ECG signs of cardiac hypertrophy were reduced [85].

For MG populations, there were no changes in ECG measures, such as PR and QRS intervals, following agalsidase beta in one relatively large placebo-controlled RCT with an open-label extension (in 58 patients followed for 26 months) [149]), and two OS reports [160,168].

##### 3.4.4.3 Mixed ERT

For mixed ERT, two OS publications reported that ECG measures did not change [113,116].

One large mixed-ERT OS publication (in 40 patients followed for 20–71 months) did not demonstrate statistically significant changes in PQ time or QRS duration in patients in the first years of ERT. However, patients analysed after some years of ERT had increased QRS duration and a trend towards increased PQ time [183].

#### 3.4.5 Exercise testing

##### 3.4.5.1 Agalsidase alfa 0.2 mg/kg EOW

For agalsidase alfa, there were no publications reporting data from clinical or OS publications for male patients. One CR of a male patient demonstrated worsened cardiac symptoms, as indicated by worsening of New York Heart Association (NYHA) functional classification to class 3, after 5 years of treatment with agalsidase alfa [57].

In an MG population, one RCT publication on 44 patients followed for 12 months showed no change in exercise testing with agalsidase alfa 0.2 mg/kg EOW, 0.2 mg/kg weekly, and 0.4 mg/kg weekly [27].

##### 3.4.5.2 Agalsidase beta 1.0 mg/kg EOW

For agalsidase beta there were no publications reporting data for male patients. In MG populations, one single-arm CT publication [156] and one OS publication [159] reported no statistically significant changes in exercise testing. In contrast, two OS publications demonstrated improvements in the bicycle stress test [168,174] and one OS publication demonstrated an improvement in NYHA class [170].

##### 3.4.5.3 Agalsidase beta 1.0 mg/kg EOW

One mixed-ERT OS publication of an MG population reported no change in exercise testing [184].

### 3.5 Nervous system outcomes

#### 3.5.1 Sweat function

##### 3.5.1.1 Agalsidase alfa 0.2 mg/kg EOW

One publication describing 30–36 months' follow-up of 26 patients treated during a placebo-controlled RCT reported immediate effects of agalsidase alfa on the ANS [37]. In the short term (24–72 hrs) after a single agalsidase alfa (0.2 mg/kg) infusion, there was a statistically significant temporary improvement in sweat function, which returned to pre-infusion treatment levels after 7 days [37]. One single-arm CT [31] and two OS publications [42,48] (in 4–97 patients followed for 12–48 months) reported improvements in sweat function and two OS publications (in 11–22 patients followed for 24–36 months) reported no change in sweat function [45,52]. Improvements in sweat function were reported in one CR of a male patient for agalsidase alfa [67] (Supplementary Table 13).

In MG publications, one RCT publication reported a non-significant increase in sweat volume with 0.1 mg/kg weekly and 0.2 mg/kg weekly compared with 0.2 mg/kg EOW agalsidase alfa [135]. In one registry OS publication (in 40 patients followed for 28–150 months), there was no change in sweat function in patients who switched from agalsidase beta to agalsidase alfa [100].

##### 3.5.1.2 Agalsidase beta 1.0 mg/kg EOW

For agalsidase beta, one single-arm CT publication (in 15 patients, of whom three received the approved dose, followed for 2.5 months) [69], and one OS publication (in 22 patients followed for ≤23 months) [77] reported subjective improvements in sweat function (Supplementary Table 13). Improvements in sweat function with agalsidase beta were also reported in three CRs in male patients [85,88,97].

In MG publications, improvements in hypohidrosis were reported in two OS publications: one study in 40 patients followed for ≥60 months [175] and another study in 11 patients followed for 29–58 months [164]. In one OS publication (in 40 patients followed for 28–150 months), there was no change in sweat function between patients switching from regular-dose to reduced-dose agalsidase beta (0.5 mg/kg EOW) [100].

##### 3.5.1.3 Mixed dose

Two CRs in male patients reported improvements in sweat function with mixed ERT [127,129].

#### 3.5.2 PNS nerve sensitivity

##### 3.5.2.1 Agalsidase alfa 0.2 mg/kg EOW

PNS nerve sensitivity with agalsidase alfa was reported in one single-arm CT publications [40] and two open-label extensions of an RCT [37,38]. After 12–18 months follow-up there was a statistically significant reduction in intra-epidermal nerve fibre density (IENFD), and no change in thermal thresholds in 26 patients [38]. When the same 26 patients were followed for 30–36 months, there was a statistically significant reduction in cold and warmth detection thresholds [37]. In a single-arm CT, switching from EOW dosing to weekly dosing of agalsidase alfa 0.2 mg/kg resulted in a worsening of warmth perception, although cold perception did not change (12 patients followed for ≤120 months) [40]. Two single-arm CT publications in the same patients reported no change in neurological examination scores after 12 months [30] and 24 months [31] (Supplementary Table 14). One CR described improvements in heat intolerance with agalsidase alfa [129].

For MG populations, there were two OS publications that reported no change in PNS nerve sensitivity following the switch from agalsidase beta 1.0 mg/kg EOW to agalsidase alfa 0.2 mg/kg EOW [100,181]: one study measured heat or cold tolerance in 40 patients after 28–150 months [100].

##### 3.5.2.2 Agalsidase beta 1.0 mg/kg EOW

With agalsidase beta, nerve sensitivity was reported to significantly improve in one OS (of 22 patients followed for 18–23 months) [77] (Supplementary Table 14). One CR described improvements in sensory ulnar and sural nerve conduction with agalsidase beta [97].

In MG publications, there was no change in heat/cold tolerance or PNS nerve sensitivity when the agalsidase beta dose was reduced [100,181].

##### 3.5.2.3 Mixed ERT

In a mixed-ERT OS publication, patients experienced an improvement in IENFD (18 patients followed for ≤103 months) [117].

#### 3.5.3 Vestibular/auditory and other CNS outcomes

##### 3.5.3.1 Agalsidase alfa 0.2 mg/kg EOW

Improvements in vestibular/auditory symptoms reported with agalsidase alfa were described in two CT publications: one was a placebo-controlled RCT with an open-label extension in seven patients followed for 24–30 months [28], and the other was a single-arm CT in five male patients followed for 12 months [34]. During the RCT portion, patients receiving placebo experienced a greater decline in hearing compared with the treated group, but the difference between groups was not statistically significant [28]. During the open-label portion, the placebo patients crossed over to agalsidase alfa and all patients experienced an improvement in hearing [28]. One OS publication reported stabilization of hearing in 11 patients followed for 25–73 months [53] (Supplementary Table 15). Neurological examination findings did not change with agalsidase alfa in one single-arm CT publication (in seven patients followed for 12 months) [30]. Two publications from the same placebo-controlled RCT in 14 patients followed for 6 months reported changes in cerebral blood flow with agalsidase alfa and placebo [32,33]. In both publications, cerebral blood flow decreased with agalsidase alfa and increased in the placebo group (Supplementary Table 15).

In MG publications, a significant improvement in hearing with agalsidase alfa was reported in one publication describing an RCT and its open-label extension [134] and in one OS publication [145], but no change in auditory function was reported in another publication [138]. One single-arm CT with agalsidase alfa reported no change in neurological examination scores [136].

##### 3.5.3.2 Agalsidase beta 1.0 mg/kg EOW

Hearing loss improved with agalsidase beta in one CR [85] but worsened in another [91]. One OS found that neurological examination scores remained stable in patients who had normal scores at baseline and were followed for 18–23 months while receiving agalsidase beta [77] (Supplementary Table 15).

In a MG population, one OS showed no change in auditory function [160].

##### 3.5.3.3 Mixed ERT

One mixed-ERT OS publication reported a decrease in hearing loss [115], another described an increased risk for tinnitus in those who were ERT antibody-positive [107], and a third reported no significant worsening in hearing acuity [105].

#### 3.5.4 White matter hyperintensities (WMH)

##### 3.5.4.1 Agalsidase alfa 0.2 mg/kg EOW

A CR (41-year-old male) with CNS lesions at baseline (vascular dolichoectasia with punctuated WMH in frontal lobe and basal ganglia) described no change in CNS lesions after 52 months of agalsidase alfa (two different doses: 0.2 mg/kg and 0.4 mg/kg) [63]. In a MG population, one single-arm CT publication (in eight patients followed for 24 months) also reported no change in WMH with agalsidase alfa [136].

##### 3.5.4.2 Agalsidase beta 1.0 mg/kg EOW

In one CR of a male patient receiving agalsidase beta, treatment resulted in disappearance of most WMH [99], but another showed an increase in WMH (with full and lower dose) [84]. One MG OS publication (in 25 patients followed for a median of 27 months) showed that, in younger patients (age <50 years), WMH remained stable in 44% of patients treated with agalsidase beta compared with 31% of patients receiving placebo [162]. Another MG OS reported no change in existing ischaemic brain lesions and no new brain lesions (in six patients followed for ≥120 months) [165].

##### 3.5.4.3 Mixed ERT

One OS publication (in 27 patients followed for 66 months) [112] and one case study [84] described the development of WMH with mixed ERT. One OS investigated WMH and other brain pathology using positron emission tomography and MRI over 9 years in patients who received mixed ERT (agalsidase beta 1.0 mg/kg EOW who were switched to agalsidase alfa 0.2 mg/kg EOW during the temporary shortage). Of 32 patients, only six received a scan before and after start of treatment and only four patients showed progression of pathology during treatment [123].

#### 3.5.5 Stroke/transient ischaemic attack (TIA)

Long-term and large-scale trials would be required to show any influence of ERT on stroke and/or TIA. However, these are difficult to perform in Fabry patients. Currently, the only publications available report on the number of patients with stroke/TIA, but none of them compares the number of strokes and/or TIAs per patient per year before and after ERT (agalsidase alfa: [30,39,50,59,136,137,144,147]; agalsidase beta: [148,150,157,159,163,175]; mixed ERT: [26,108,111,112,114,118,120,123,127,128,177,181,186]).

### 3.6 Pain outcomes

##### 3.6.1.1 Agalsidase alfa 0.2 mg/kg EOW

With agalsidase alfa, improvements in subjectively assessed acroparaesthesiae were reported in one single-arm CT publication (in seven patients followed for 24 months) [31]. In a placebo-controlled RCT, 14 patients treated with agalsidase alfa experienced a decrease in pain and pain-related QoL while data in the 12 patients receiving placebo remained mostly unchanged [36]. Significantly improved pain scores were reported in 14 patients followed for 6 months [36]. The open-label extension study where the same patients were followed for a further 30 months reported no further decline in pain scores [37]. A large registry OS publication (in 345 patients, of whom 51 had Brief Pain Inventory [BPI] data available) followed for ≤36 months) showed improvements in average pain and present pain assessed with the BPI, but no changes in worst pain and last pain [47]. Improvements in neuropathic pain/acroparaesthesiae were seen in one OS publication with agalsidase alfa (in four patients followed for 12 months) [42]. In contrast, three other OS publications did not report statistically significant changes in pain scores (in 11 patients followed for 25–73 months [53], in 172 patients followed for 48 months in a registry study [48], and in five patients followed for ≤12 months [55]) (Supplementary Table 16). Most of the CRs of agalsidase alfa also described improvements in neuropathic pain/acroparaesthesiae or pain scores [58,[60], [61], [62],^64^].

One MG RCT publication reported an improvement in ‘pain-at-its worst’ scores with agalsidase alfa 0.1 mg/kg/week and 0.2 mg/kg/week compared with pre-treatment levels, but no differences in mean pain score [135]. Two large registry OS publications reported reductions in BPI pain scores during ERT (in 181 patients followed for 60 months [144], and in 314 patients followed for a mean of 17 months [137]). One RCT publication reported no change in pain with agalsidase alfa [176], which was also seen in an OS publication in which patients switched from agalsidase beta 1.0 mg/kg EOW to agalsidase alfa 0.2 mg/kg EOW [177]. One registry OS publication (in 40 patients followed for 28–150 months) reported no change in pain in patients who switched from agalsidase beta 1.0 mg/kg EOW to agalsidase alfa 0.2 mg/kg EOW [100]. One large OS publication (in 105 patients followed for 8–16 months) reported statistically significant increases in pain attacks when switching from agalsidase beta 1.0 mg/kg EOW to agalsidase alfa 0.2 mg/kg EOW [181].

##### 3.6.1.2 Agalsidase beta 1.0 mg/kg EOW

With agalsidase beta, one single-arm CT publication reported a statistically significant improvement in pain scores (in 15 patients across all five dosing regimens followed for 2.5 months) [69], while another single-arm CT publication reported a near-significant reduction in pain intensity (in 13 patients followed for 5 months) [70]. One OS also found that treatment with agalsidase beta for 18 or 23 months significantly reduced the mean total symptom score for neuropathic pain in a group of 22 Fabry disease patients [77]. Pain was also improved in an OS of three patients after 18 months [80] (Supplementary Table 16). Many CRs with agalsidase beta also described reduced neuropathic pain/acroparaesthesiae or improved pain scores [84,85,89,97].

In MG publications with agalsidase beta, improvements in pain scores were reported from a placebo-controlled RCT with open-label extension in 58 patients [149], who were then followed through open-label extension studies for a further 30 months [157] and 54 months [150], and one RCT publication using a reduced dose of 0.2 mg/kg EOW [176]. During the placebo-controlled portion of the RCT, patients in both groups experienced a decline in pain scores due to a possible placebo effect, so the difference between groups was not statistically significant [149]. In one single-arm CT, patients were able to discontinue their use of analgesics [151]. Three OS publications reported decreased pain with agalsidase beta: reduction in neuropathic pain (40 patients followed for ≥60 months) [175], resolution of neuropathic pain [164] and reduction in acroparaesthesiae [168]. No change in pain symptoms was reported for agalsidase beta 1.0 mg/kg EOW, but lowering the dose to 0.3–0.5 mg/kg EOW resulted in an increase in pain crises and pain attacks in the relatively large OS in 105 patients followed for 8–16 months [181]. Another registry OS publication (in 40 patients followed for 28–150 months) reported no change in pain in patients who switched from regular- to reduced-dose agalsidase beta [100].

##### 3.6.1.3 Mixed ERT

One mixed-ERT OS publication observed a greater improvement in pain scores in ERT antibody-negative patients compared with antibody-positive patients [107]. Most of the mixed-ERT CRs described improved pain [119,124,125,127,129]. In one CR, two patients showed an increase in pain scores after switching from agalsidase beta 1.0 mg/kg EOW to agalsidase alfa 0.2 mg/kg EOW, which was reversed when agalsidase beta 1.0 mg/kg EOW was reintroduced [125]. Furthermore, patients receiving agalsidase beta 1.0 mg/kg EOW were able to stop anti-neuropathic treatment or reduce medication, whereas those receiving agalsidase alfa 0.2 mg/kg EOW continued using carbamazepine during the study [125]. An 18-year-old male had acral pain at baseline, which was reduced with agalsidase beta (1.0 mg/kg EOW) but markedly increased when the patient was switched to agalsidase alfa 0.2 mg/kg EOW [130].

One mixed-ERT MG OS publication reported a reduction in acroparaesthesiae [184]. In one OS, there was a statistically significant association between time on mixed ERT and pain interference score, but no significant association with pain severity [103].

### 3.7 Gastrointestinal (GI) outcomes

#### 3.7.1 Agalsidase alfa 0.2 mg/kg EOW

Improvement in GI outcomes (i.e. abdominal pain, constipation, nausea, and vomiting) with agalsidase alfa was reported in one single-arm CT (five patients followed for 24 months) [31] and two OS publications (in 33 male patients followed for 24 months [46] and in up to 77 male patients followed for on average 48 months in a registry study [48]) (Supplementary Table 17). Most (4 of 5) CRs with agalsidase alfa also observed reduced GI outcomes [59,62,67,190] but one CR described worsening abdominal pain, bloating, postprandial vomiting, and sporadic diarrhoea after 4 years of treatment [65].

One CT publication in an MG population reported a reduction in the number of patients with abdominal pain and/or diarrhoea during treatment with agalsidase alfa [140]. Switching from agalsidase beta 1.0 mg/kg EOW to agalsidase alfa 0.2 mg/kg EOW resulted in an increase in GI pain in two relatively large, mixed-ERT OS publications (in 105 patients followed for 8–16 months [181], and in 89 patients followed for 24 months [177]).

#### 3.7.2 Agalsidase beta 1.0 mg/kg EOW

With agalsidase beta, improvement of GI outcomes (i.e. abdominal pain, diarrhoea, and discontinuation of GI medications) was reported in three CRs [74,85,97].

In MG publications, agalsidase beta resulted in an improvement in GI outcomes in one single-arm CT publication [151] and resolution of abdominal pain in one OS publication [164]. In one relatively large OS, there was no change in GI pain and diarrhoea with regular-dose agalsidase beta, but dose reduction led to increased GI pain (in 89 patients followed for 24 months) [177]. A relatively large, mixed-ERT OS publication reported no changes in GI outcomes (general or pain/diarrhoea) with regular- or reduced-dose agalsidase beta (in 105 patients followed for 8–16 months) [181].

#### 3.7.3 Mixed ERT

Positive effects of ERT on GI outcomes were confirmed in a mixed-ERT OS publication in which diarrhoea was reduced in antibody-negative patients (in 24 patients followed for ≤131 months) [107], and in one CR [127]. Another CR noted that GI discomfort was reduced with agalsidase beta 1.0 mg/kg EOW but increased when the patient was switched to agalsidase alfa 0.2 mg/kg EOW [130].

One mixed-ERT OS publication reported no changes in GI outcomes (general or pain/diarrhoea) after switching from regular- to reduced-dose agalsidase beta or regular-dose agalsidase alfa (in a registry study of 40 patients followed for 28–150 months) [100].

### 3.8 Quality of life (QoL)

#### 3.8.1 Agalsidase alfa 0.2 mg/kg EOW

One large registry OS publication (in 172 patients, of whom 30–37 had QoL data, followed for on average 48 months) reported non-significant improvements in health scores from the 5-dimension EuroQol questionnaire and EuroQol visual analogue scale with agalsidase alfa [48]. Another relatively large, mixed-ERT registry OS publication reported no change in self-reported energy levels (using the 36-Item Short Form Health Survey [SF-36]) with agalsidase alfa (in 32 patients treated for 12 months with agalsidase alfa) [100]. One mixed-ERT OS publication reported no statistically significant difference in SF-36 scores after a switch from agalsidase beta 1.0 mg/kg EOW to agalsidase alfa 0.2 mg/kg EOW [54] (Supplementary Table 18). A CR described an increase in motivation after 12 months with agalsidase alfa [66].

In MG publications, findings varied. In two RCTs, QoL measures did not change with agalsidase alfa 0.2 mg/kg EOW, 0.1 mg/kg weekly, or 0.2 mg/kg weekly [135], or with agalsidase alfa 0.2 mg/kg EOW, 0.2 mg/kg weekly, or 0.4 mg/kg weekly [27], but did increase (measured using the EuroQoL index) with agalsidase alfa 0.2 mg/kg EOW in a large registry OS publication (in 181 patients followed for 60 months) [144]. There was no change in Center for Epidemiologic Studies Depression Scale (CES-D) score upon switching from agalsidase beta 1.0 mg/kg EOW to agalsidase alfa 0.2 mg/kg EOW in one relatively large, mixed-ERT OS publication (in 105 patients followed for 8–16 months) [181].

#### 3.8.2 Agalsidase beta 1.0 mg/kg EOW

For agalsidase beta, two single-arm CT publications reported improvements in general and mental health using the SF-36 (statistically significant in 13 patients followed for 5 months [70]), and improvements in bodily pain, general health, and vitality in the SF-36 (in 15 patients, three of whom received 1.0 mg/kg EOW, followed for 2.5 months [69]) (Supplementary Table 18). One registry OS publication (in 71 patients followed for an average of 81 months) reported a statistically significant improvement in mental and physical health (using the SF-36) after 12 months of agalsidase beta [83]. One mixed-ERT registry OS publication reported a decrease in self-reported energy levels (using the SF-36) when the agalsidase beta dose was reduced to 0.3–0.5 mg/kg EOW (in 32 patients followed for 18–74 months) [100] (Supplementary Table 18). One CR demonstrated the resolution of a diagnosis of depressive disorder after 12 months of agalsidase beta [97], while another CR showed improvements in functional independence (using the Functional Independence Measure) in six patients with agalsidase beta [98].

For MG publications with agalsidase beta 1.0 mg/kg EOW, one placebo-controlled RCT with open-label extension [149] in 58 patients, two open-label extension studies (where the same patients were followed for a further 30–36 months [157] and up to 54 months [150]), and one OS publication [165] described improvements in QoL (using the SF-36). A different OS publication (nine patients followed for 24 months) reported no change [168].

#### 3.8.3 Mixed ERT

One mixed-ERT OS publication reported no statistically significant difference in CES-D scores [117].

In MG populations, a relatively large, mixed-ERT OS publication in 105 patients followed for 8–16 months reported no change in QoL [181]. Another mixed-ERT registry OS publication (in 32 male patients followed for 18–74 months) reported no change in QoL between patients switching from regular- to reduced-dose agalsidase beta and patients switching from regular-dose agalsidase beta to regular-dose agalsidase alfa [100].

### 3.9 Clinical events

Large, long-term trials are needed to show an effect of ERT on incidence rates of clinical events, such as renal, cardiac, and cerebrovascular events, and death. The majority of papers describing clinical events before or during ERT only report the number of events occurring during the study without a statistical analysis of the data. There were no agalsidase alfa publications describing an ERT effect on clinical events. One placebo-controlled RCT [148], one publication in which patients were treated during an RCT, its open-label extension and then followed through a registry [163], and two large OS publications [78,172] reported an effect of agalsidase beta 1.0 mg/kg EOW on the timing and incidence of severe clinical events. One large CT with 52 patients (including two severely affected females) showed that during 10 years of treatment 81% of patients did not experience any severe clinical event and 94% of patients were still alive at the end of the study [163]. One OS publication described a decrease in incidence rate of severe clinical events after 6 months of treatment, which was sustained during the remaining 4.5 years of the study [172]. Two registry OS publications showed that the risk of a clinical event was significantly lower when treatment was started at a younger age (<40 years) [78,172], and one placebo-controlled RCT demonstrated a significant 81% risk reduction for life-threatening events compared with placebo when treatment was started before renal deterioration [148].

### 3.10 Male patients with later-onset phenotypes

Patients with later-onset phenotypes of Fabry disease often present with later-onset symptoms that may be confined to a single-organ system. Although several publications may describe outcomes for a mix of patients, including patients with classic disease, later-onset disease or variants of unknown significance, we identified outcome data specific for adult male patients with later-onset phenotypes of Fabry disease in five mixed-ERT MG OS publications. These studies included three small studies of eight to 35 patients followed for 3–90 months [105,109,115], a relatively large study in 109 patients followed for ≤116 months [103], and one study describing the results from three patients who switched from agalsidase beta 1.0 mg/kg EOW to agalsidase alfa 0.2 mg/kg EOW, and 13 patients who received agalsidase alfa 0.2 mg/kg EOW [110]. Findings from these studies are outlined below.

For 18 patients (13 males and five females) with a predominantly cardiac phenotype (IVS4+919G>A) who received agalsidase alfa or beta for 6–39 months, echocardiography revealed the reduction or stabilization of LVMi, IVST, and left posterior wall thickness (LPWT) [109]. However, since this mutation affects mRNA splicing, it may not be representative for the late-onset cardiac variant in patients with missense later-onset mutations in the coding *GLA* regions.

In another study of 16 patients with a predominantly cardiac phenotype (IVS4+919G>A) who received agalsidase alfa or beta for at least 12 months, LVMi decreased or remained stable [110]. In a relatively large study including 37 male later-onset type patients followed for ≤116 months, ERT (with agalsidase alfa or beta) was reported to reduce LVMi and improve eGFR [103].

No publications containing data for male patients with later-onset phenotypes who received ERT were identified in this analysis for urinary (lyso-) GL-3, kidney or cardiac GL-3, or GL-3 levels in other organs, ECG measures or exercise testing, other ANS, PNS, or CNS outcomes, GI symptoms, or QoL.

## 4 Discussion

This systematic literature review includes all types of publication with original data, any type of ERT regimen, and all Fabry disease-related outcomes in the population of male Fabry patients over the past 16 years. Spanning 166 publications including 36 CTs, it provides a comprehensive overview of outcomes achieved in male patients with Fabry disease when treated with disease-specific therapy.

Analyzing this volume of evidence was a huge undertaking, adding to the current literature in Fabry disease. First, it provides an overview of all the evidence published exclusively in male patients. Previous systematic literature reviews [197] have pooled data in male and female patients, which may not be appropriate considering the recognized differences in clinical manifestations and disease progression. Secondly, data from the current review in males and two other publications, in females [22] and paediatric patients [23], informed the development of new therapeutic goals for Fabry disease [24]. This therapeutic goals publication outlines what patients should expect to achieve in each organ system, depending on their individual disease status at baseline [24]. The therapeutic goals publication was based on the evidence published in males (current review, total 166 publications including 36 RCTs), in females (total 67 publications including 6 RCTs) and paediatric patients (total 16 publications, including 6 RCTs). As the reader can note, most of the evidence is derived from the current review, in male patients. Finally, the current review provides a comprehensive summary of the effects of ERT on all organ systems. This helps inform clinical decision-making and the management of male Fabry patients.

ERT was the first disease-specific therapy available that changed the natural history of Fabry disease. Data published in adult male patients with Fabry disease demonstrates that the effect of ERT on plasma GL-3 levels, eGFR, and cardiac outcomes is strongest and substantiated by a wide range of publications, showing consistent, dose-dependent reductions in GL-3 accumulation, a reduced decline in eGFR, and improvements in cardiac outcomes (see relevant sections within the current review). Furthermore, ERT may improve nervous system, GI, pain, and QoL outcomes. In the context of a multisystemic condition, the impact of ERT on all affected organs is important and relevant. Some studies show no change in outcomes over time with ERT. However, considering the fact that Fabry disease is a lifetime progressive debilitating disease associated with a risk of premature mortality, no change in outcomes is an indication of disease stabilization. This indicates that a clinical benefit in preventing disease progression and avoiding deterioration of patients’ well-being is seen when no difference in outcomes after ERT compared with baseline is observed.

Patients who started ERT at an earlier age achieved better outcomes [7,82,130,163,174], and, therefore, effective early treatment is needed to prevent or mitigate disease progression [196]. Regarding cardiac function, patients who started agalsidase beta treatment at age <30 years experienced a statistically significant decline in LVM but those who started ERT at age ≥50 years reported an increase in LVM [7], and patients <40 years of age at treatment initiation had stable LPWT and IVST over a period of 10 years, whereas patients ≥40 years of age at treatment initiation significantly progressed over time [163]. Likewise, the importance of administering ERT early was demonstrated in patients without fibrosis at baseline. They experienced a statistically significant decline from baseline in LVM and a statistically significant improvement in exercise capacity with ERT, while patients in whom treatment was started when mild or severe fibrosis was already present experienced no effect of ERT [174]. Regarding renal function, initiation of treatment before the development of significant glomerulosclerosis and proteinuria might prevent future renal disease [79,163], which correlates with the reduced slope of eGFR decline that is seen in patients who started ERT early compared with a higher slope of eGFR decline in patients who started ERT later [82]. Furthermore, two recent publications derived from the Fabry Registry demonstrated a statistically significantly lower risk of clinical events in patients who started ERT <40 years of age compared with those who were older at start of ERT [78,172], and one placebo-controlled RCT showed that ERT initiation before the start of severe organ damage gives a lower risk of the occurrence of clinical events [148]. On a smaller scale, a case series noted that the GL-3 clearance from podocytes was more extensive in the patient who started ERT at 7 years of age compared with the patient who started at 18 years [130].

Consolidated evidence suggests a dose effect. Studies which have included both agalsidase alfa 0.2 mg/kg EOW and agalsidase beta 1.0 mg/kg EOW show more notable reductions of plasma GL-3, plasma lyso-GL-3, and urinary GL-3 with agalsidase beta 1.0 mg/kg EOW [101,102]. A dose increase of agalsidase alfa beyond the approved dose has a positive effect on several outcomes including plasma GL-3 [27,52], urinary GL-3 [52], eGFR [40], proteinuria [52,62,67], and sweat function [135]. In contrast, reducing the agalsidase beta dose or switching from agalsidase beta 1.0 mg/kg EOW to agalsidase alfa 0.2 mg/kg EOW, negatively impacts some outcomes. The literature shows that agalsidase beta dose reduction causes worsening of plasma (lyso-) GL-3 plasma [54,72], urinary GL-3 [72], renal GL-3 [72], pain [181], GI symptoms [177], and self-reported energy levels [100]. Furthermore, the switch from agalsidase beta (1.0 mg/kg EOW) to agalsidase alfa (0.2 mg/kg EOW) also had a negative impact on plasma lyso-GL-3 [54], urinary GL-3 [26], renal GL-3 [130], albuminuria [181], pain [125,130], and GI symptoms [130,177,181]. A dose effect is further exemplified by a normalization of plasma GL-3 levels with agalsidase beta 1.0 mg/kg EOW in several studies (Supplementary Table 2) [69,70,72,75,79,102]. The largest head-to-head comparison between agalsidase alfa and agalsidase beta demonstrated that 19.4% of patients on agalsidase alfa and 13.3% of patients on agalsidase beta progressed to a composite clinical endpoint (renal, cardiovascular, or cerebrovascular events, or death) during the 59-month study duration, although differences were not significant due to limited power [114].

The design and limitations of the systematic literature analysis, which was used to identify and extract the data reported in this publication, are described in detail in a separate manuscript [21]. Data reported in patients with later-onset Fabry disease largely reflect the outcomes seen in classic male patients. However, many publications included in our analysis may have described outcomes for a mix of patients with classic disease, later-onset disease or variants of unknown significance, or did not specify whether patients had classic or later-onset disease. Therefore, the inclusion of patients with later-onset phenotypes in clinical studies looking at outcomes related to organs that are not affected in such patients could be a source of error. Our review was limited to ERT and did not include all the emerging treatment approaches for Fabry disease such as migalastat [[191], [192], [193]] and PRX-102 [194]. Furthermore, our analysis did not stratify the renal or cardiac outcomes according to concomitant medications, which may be relevant considering the effect of medications used to treat renal disease (ACEi and/or ARB) on the progression of Fabry nephropathy [195]. Furthermore, the use of antiproteinuric therapy is only reported for about half of the publications that report on albuminuria or proteinuria, which is important for putting results in perspective.

ERT, available since 2001, was the first disease-specific treatment for Fabry patients, providing a clinical benefit to thousands of patients to date with this rare disease. Fabry disease is a multisystemic disease and ERT has an effect on most outcomes and organ systems, resulting in slowing or halting of cardiac and renal disease progression. Evidence, mainly derived from male studies, shows that patients may benefit from treatment that starts early, before major organ damage has developed. Consolidated evidence suggests a dose effect. Together, the data described in male, female, and paediatric patient populations inform clinical practice in order to facilitate individualized treatment, aiming for optimized patient care and improvement in QoL.

## Appendix A Supplementary data

Supplementary material Image 1
