# Supplementary material for: The effect of enzyme replacement therapy on clinical outcomes in male patients with Fabry disease: A systematic literature review by a European panel of experts
Source: Mol Genet Metab Rep. 2019 Feb 6;19:100454. doi: 10.1016/j.ymgmr.2019.100454 (PMC6365982; doi:10.1016/j.ymgmr.2019.100454)
Supplement: Supplementary file 1 — Supplementary material [file mmc1.docx]

Contents

[Supplementary Table 1a Publications describing clinical outcomes with ERT in studies of adult male patients with Fabry disease 2](#_Toc517181572)

[Supplementary Table 1b Publications describing clinical outcomes with ERT in mixed-gender studies (populations including ≥50% male patients with Fabry disease) 13](#_Toc517181573)

[Supplementary Table 2 Plasma GL-3 outcomes with approved doses of agalsidase alfa and agalsidase beta in adult male patients 22](#_Toc517181574)

[Supplementary Table 3 Plasma lyso-GL-3 outcomes with approved doses of agalsidase alfa and agalsidase beta in adult male patients 25](#_Toc517181575)

[Supplementary Table 4 Urinary GL-3 outcomes with approved doses of agalsidase alfa and agalsidase beta in adult male patients 27](#_Toc517181576)

[Supplementary Table 5 Kidney GL-3 accumulation outcomes with approved doses of agalsidase alfa and agalsidase beta in adult male patients 29](#_Toc517181577)

[Supplementary Table 6 Cardiac GL-3 accumulation outcomes with approved dose of agalsidase alfa in adult male patients 31](#_Toc517181578)

[Supplementary Table 7 GL-3 accumulation outcomes in other organs with approved dose of agalsidase beta in adult male patients 32](#_Toc517181579)

[Supplementary Table 8 Proteinuria outcomes with approved doses of agalsidase alfa and agalsidase beta in adult male patients 33](#_Toc517181580)

[Supplementary Table 9 Left ventricular hypertrophy outcomes with approved doses of agalsidase alfa and agalsidase beta in adult male patients 35](#_Toc517181581)

[Supplementary Table 10 Wall thickness outcomes with approved doses of agalsidase alfa and agalsidase beta in adult male patients 38](#_Toc517181582)

[Supplementary Table 11 Ejection fraction outcomes with approved doses of agalsidase alfa and agalsidase beta in adult male patients 39](#_Toc517181583)

[Supplementary Table 12 Electrocardiography outcomes with approved doses of agalsidase alfa and agalsidase beta in adult male patients 40](#_Toc517181584)

[Supplementary Table 13 Sweat function outcomes with approved doses of agalsidase alfa and agalsidase beta in adult male patients 42](#_Toc517181585)

[Supplementary Table 14 Nerve sensitivity outcomes with approved doses of agalsidase alfa and agalsidase beta in adult male patients 44](#_Toc517181586)

[Supplementary Table 15 Vestibular/auditory and other central nervous system outcomes with approved doses of agalsidase alfa and agalsidase beta in adult male patients 46](#_Toc517181587)

[Supplementary Table 16 Pain outcomes with approved doses of agalsidase alfa and agalsidase beta in adult male patients 48](#_Toc517181588)

[Supplementary Table 17 Gastrointestinal outcomes with approved dose of agalsidase alfa in adult male patients 51](#_Toc517181589)

[Supplementary Table 18 Quality of life outcomes with approved doses of agalsidase alfa and agalsidase beta in adult male patients 52](#_Toc517181590)

# Supplementary Table 1a Publications describing clinical outcomes with ERT in studies of adult male patients with Fabry disease

| **Treatment** | **Study, year**  **[reference]** | **Evidence Grade** | **Dose** | **Duration^a^ (months)** | **ERT-treated male patients (n)** | **Clinical outcomes reported** |
| --- | --- | --- | --- | --- | --- | --- |
| **Agalsidase alfa** | Clarke et al. 2007  [25] | Clinical: Grade 1a randomized controlled trial | 0.1 mg/kg weekly 0.2 mg/kg EOW 0.2 mg/kg weekly 0.4 mg/kg EOW 0.4 mg/kg weekly | 2.5 | 18 | Plasma GL-3 |
|  | Goker-Alpan et al. 2015  [26] | Clinical: Grade 1c single-arm clinical trial | 0.2 mg/kg EOW | 24 | 15 (naïve) 40 (switch) | Plasma GL-3,  plasma lyso-GL-3,  urinary GL-3 |
|  | Goláň L. et al. 2015  [27] | Clinical: Grade 1a randomized controlled trial | 0.2 mg/kg EOW 0.2 mg/kg weekly 0.4 mg/kg weekly | 12 | 26 | Plasma GL-3, LVM |
|  | Hajioff et al. 2003  [28] | Clinical: Grade 1a/c | 0.2 mg/kg EOW | 24–30 | 7 | Hearing loss |
|  | Hughes et al. 2008  [29] | Clinical: Grade 1a/c | 0.2 mg/kg EOW | 30 | 7 | Plasma GL-3, urinary GL-3, cardiac GL-3, eGFR, LVM, EF, ECG |
|  | Jardim et al. 2004  [30] | Clinical: Grade 1c single-arm clinical trial | 0.2 mg/kg EOW | 12 | 7 | Neurological examination/CNS, TIA/stroke |
|  | Jardim et al. 2006  [31] | Clinical: Grade 1c single-arm clinical trial | 0.2 mg/kg EOW | 24 | 7 | Acroparaesthesia, neurological examination, GI outcomes |
|  | Moore et al. 2001  [32] | Clinical: Grade 1a randomized controlled trial | 0.2 mg/kg EOW | 6 | 14 | Cerebral blood flow |
|  | Moore et al. 2002  [33] | Clinical: Grade 1a randomized controlled trial | ERT NS | 6 | 14 | Cerebral blood flow |
|  | Palla et al. 2003  [34] | Clinical: Grade 1c single-arm clinical trial | 0.2 mg/kg EOW | 12 | 5 | Vestibular function |
|  | Pastores et al. 2007  [35] | Clinical: Grade 1c single-arm clinical trial | 0.2 mg/kg EOW | 6 | 22 | Plasma GL-3, urinary GL-3, eGFR |
|  | Schiffmann et al. 2001  [36] | Clinical: Grade 1a randomized controlled trial | 0.2 mg/kg EOW | 5.5 | 14 | Plasma GL-3, urinary GL-3, kidney GL-3, eGFR, proteinuria, ECG, pain |
|  | Schiffmann et al. 2003  [37] | Clinical: Grade 1c single-arm clinical trial | 0.2 mg/kg EOW | 30–36 | 26 | Sweat function, PNS nerve sensitivity, pain |
|  | Schiffmann et al. 2006  [38] | Clinical: Grade 1c single-arm clinical trial | 0.2 mg/kg EOW | 12–18 | 26 | eGFR, PNS nerve sensitivity |
|  | Schiffmann et al. 2006  [39] | Clinical: Grade 1c single-arm clinical trial | 0.2 mg/kg EOW | 48–54 | 14 | Plasma GL-3, urinary GL-3, eGFR, proteinuria, TIA/stroke |
|  | Schiffmann et al. 2015  [40] | Clinical: Grade 1c single-arm clinical trial | 0.2 mg/kg weekly | <120 | 12 | Plasma GL-3, urinary GL-3, eGFR, PNS nerve sensitivity |
|  | Beck et al. 2015  [41] | Observational: Grade 3 retrospective observational study | 0.2 mg/kg EOW | 60 | 360 | eGFR, LVM |
|  | Bongiorno et al. 2003  [42] | Observational: Grade 2 prospective observational study | 0.2 mg/kg EOW | 12 | 4 | Proteinuria, sweat function, pain |
|  | Feriozzi et al. 2009  [43] | Observational: Grade 3 retrospective observational study | 0.2 mg/kg EOW | 36 | 115 | eGFR, proteinuria |
|  | Feriozzi et al. 2012  [44] | Observational: Grade 3 retrospective observational study | 0.2 mg/kg EOW | 88.8 | 134 | eGFR, proteinuria |
|  | Gupta et al. 2008  [45] | Observational: Grade 2 prospective observational study | 0.2 mg/kg EOW | 36 | 22 | Sweat function |
|  | Hoffmann et al. 2007  [46] | Observational: Grade 3 retrospective observational study | 0.2 mg/kg EOW | 24 | 345 | GI outcomes |
|  | Hoffmann et al. 2007  [47] | Observational: Grade 3 retrospective observational study | 0.2 mg/kg EOW | ≤36 | 345 | Pain |
|  | Hughes et al. 2011  [48] | Observational: Grade 3 retrospective observational study | 0.2 mg/kg EOW | ≥48 | 172 | eGFR, proteinuria, LVM, sweating, pain, GI outcomes, QoL |
|  | Kampmann et al. 2015 [49] | Observational: Grade 3 retrospective observational study | 0.2 mg/kg EOW | 130 (median) | 24 | eGFR, proteinuria, LVM, LVWT, EF, ECG |
|  | Kaneski et al. 2006  [50] | Observational: Grade 2 prospective observational study | 0.2 mg/kg EOW | ≤55 | 73 | TIA/stroke |
|  | Lin et al. 2014 [51] | Observational: Grade 3 retrospective observational study | 0.2 mg/kg EOW | Beta:  0.7–88.6 Alfa:  12–112 | 9 | Plasma lyso-GL-3, eGFR, albuminuria, LVM |
|  | Schiffmann et al. 2007  [52] | Observational: Grade 2 prospective observational study | 0.2 mg/kg EOW 0.2 mg/kg weekly | 24–48 | 12 | Plasma GL-3, urinary GL-3, eGFR, proteinuria,  sweat function |
|  | Sergi et al. 2010  [53] | Observational: Grade 2 prospective observational study | 0.2 mg/kg EOW | 51.5  (25–73) | 11 | Hearing, pain |
|  | Smid et al. 2011 [54] | Observational: Grade 3 retrospective observational study | 0.2 mg/kg EOW | 64.8  (3–123) | NR | Plasma lyso-GL-3, eGFR, QoL |
|  | Tsuboi et al. 2012  [189] | Observational: Grade 2 prospective observational study | Alfa: 0.2 mg/kg EOW  (pre-switch:  beta 1.0 mg/kg EOW) | 12 | 4 | LVM, LVWT |
|  | Whitfield et al. 2005  [55] | Observational: Grade 2 prospective observational study | 0.2 mg/kg EOW | 12 | 6 | Urinary GL-3, pain |
|  | Altarescu et al. 2011  [56] | Case: Grade 5 case report | 0.2 mg/kg EOW | 8 | 1 | Proteinuria |
|  | Feriozzi et al. 2007 [190] | Case: Grade 5 case report | 0.2 mg/kg EOW | 3 | 1 | GI outcomes |
|  | Frustaci et al. 2016  [57] | Case: Grade 5 case report | 0.2 mg/kg EOW | 108 | 1 | LVM, LVWT, EF, ECG, cardiac function (NYHA) |
|  | Ghali et al. 2012  [58] | Case: Grade 4 case series | Agalsidase alfa dose NS | NR | 3 | Pain |
|  | Gomez et al. 2012  [59] | Case: Grade 4 case series | 0.2 mg/kg EOW | 6–72 | 4 | LVM, ECG, TIA/stroke,  GI outcomes |
|  | Kampmann et al. 2013  [60] | Case: Grade 5 case report | ERT NS | 144 | 1 | eGFR, LVM, pain |
|  | Karras et al. 2008  [61] | Case: Grade 5 case report | 0.2 mg/kg EOW | 42 | 1 | LVM, pain |
|  | Nicholls et al. 2012  [62] | Case: Grade 5 case report | 0.2 mg/kg EOW | 60 | 1 | eGFR, proteinuria, pain,  GI outcomes |
|  | Paliouras et al. 2015  [63] | Case: Grade 5 case report | 0.2 mg/kg EOW  0.4 mg/kg EOW | 52 | 1 | Proteinuria, LVM, WMH |
|  | Pieroni et al. 2013  [64] | Case: Grade 5 case report | 0.2 mg/kg EOW | 72 | 1 | Proteinuria, LVM, LVWT, ECG, pain |
|  | Politei et al. 2016  [65] | Case: Grade 4 case series | 0.2 mg/kg EOW | 48 | 1 | Proteinuria, GI outcomes |
|  | Tomizawa et al. 2015  [66] | Case: Grade 5 case report | 0.2 mg/kg EOW | 12 | 1 | Motivation (QoL) |
|  | Torra et al. 2008  [67] | Case: Grade 5 case report | 0.2 mg/kg EOW 0.4 mg/kg EOW | 72 | 1 | eGFR, proteinuria, sweat function, GI outcomes |
|  | Yano et al. 2016  [68] | Case: Grade 5 case report | 0.2 mg/kg EOW | 72 | 1 | LVH |
| **Agalsidase beta** | Eng et al. 2001  [69] | Clinical: Grade 1c single-arm clinical trial | 0.3 mg/kg EOW 1.0 mg/kg EOW 3.0 mg/kg EOW 1.0 mg/kg every 48 h 3.0 mg/kg every 48 h | 2.5 | 15 | Plasma GL-3, renal GL-3, cardiac GL-3, liver GL-3, ECG, sweat function, pain, QoL |
|  | Eto et al. 2005  [70] | Clinical: Grade 1c single-arm clinical trial | 1.0 mg/kg EOW | 5 | 13 | Plasma GL-3, urinary GL-3, eGFR, pain, QoL |
|  | Goker-Alpan et al. 2016  [71] | Clinical: Grade 1c single-arm clinical trial | 1.0 mg/kg EOW | 6 | 15 | Plasma GL-3,  plasma lyso-GL-3,  urinary GL-3 |
|  | Lubanda et al. 2009  [72] | Clinical: Grade 1c single-arm clinical trial | 1.0 mg/kg EOW 0.3 mg/kg EOW | Normal dose: 6 Low dose: 18 | 21 | Plasma GL-3, urinary GL-3, renal GL-3, dermal GL-3, eGFR, proteinuria |
|  | Najafian et al. 2016  [73] | Clinical: Grade 1c single-arm clinical trial | 1.0 mg/kg EOW | 11–12 | 6 | Renal GL-3, eGFR, proteinuria |
|  | Bénichou et al. 2009  [75] | Observational: Grade 3 retrospective observational study | 1.0 mg/kg EOW | 1.9–60.7 | 122 | Plasma GL-3, dermal GL-3 |
|  | Elliott et al. 2006  [76] | Observational: Grade 2 prospective observational study | 1.0 mg/kg EOW 2.0 mg/kg EOW | 10.1 (2.3) | 10 | Plasma GL-3, LVM, LVWT |
|  | Germain et al. 2013  [7] | Observational: Grade 3 retrospective observational study | 0.9–1.1 mg/kg EOW | 58 (22–114) | 115 | LVM |
|  | Hilz et al. 2004  [77] | Observational: Grade 2 prospective observational study | 0.9–1.0 mg/kg EOW | 18 or 23 | 22 | Sweat function, PNS nerve sensitivity, neurological findings, pain |
|  | Hopkin et al. 2016  [78] | Observational: Grade 3 retrospective observational study | 0.9–1.1 mg/kg EOW | 52 | 969 | Clinical events |
|  | Kim et al. 2016 [79] | Observational: Grade 2 prospective observational study | 1.0 mg/kg EOW | 60–126 | 11 | eGFR, proteinuria, LVMi |
|  | Mignani et al. 2004 [80] | Observational: Grade 2 prospective observational study | 0.9–1.0 mg/kg EOW | 18 | 3 | Plasma GL-3, urinary GL-3, proteinuria, LVM, LVWT, EF ECG, pain |
|  | Motwani et al. 2012  [81] | Observational: Grade 3 retrospective observational study | 1.0 mg/kg EOW | 36 (median) | 44 | LVM, LVWT, EF, ECG |
|  | Smid et al. 2011 [54] | Observational: Grade 3 retrospective observational study | 0.2 mg/kg EOW | 64.8  (3–123) | NR | Plasma lyso-GL-3 |
|  | Warnock et al. 2012  [82] | Observational: Grade 3 retrospective observational study | 1.0 mg/kg EOW | 24 | 151 | eGFR |
|  | Watt et al. 2010  [83] | Observational: Grade 3 retrospective observational study | 1.0 mg/kg EOW | 81 ± 31 | 71 | QoL |
|  | Arends et al. 2016  [84] | Case: Grade 4 case series | 1.0 mg/kg EOW and “reduced dose” | 192 | 1 | eGFR, LVM, WMH, pain |
|  | Banikazemi et al. 2005  [74] | Observational: Grade 4 Case series | 1.0 mg/kg EOW | 6─7 | 4 | GI outcomes |
|  | Bazan-Socha et al. 2007  [85] | Case: Grade 4 case series | 1.0 mg/kg EOW | 54 | 2 | Proteinuria, EF, ECG,  sweat function, hearing, pain, GI outcomes |
|  | Choi et al. 2009  [86] | Case: Grade 5 case report | 1.0 mg/kg EOW | 19 | 1 | Proteinuria, EF |
|  | De Schoenmakere et al. 2003  [87] | Case: Grade 5 case report | 1.0 mg/kg EOW | 19 | 1 | Proteinuria, LVM, LVWT |
|  | Fauchais et al. 2010  [88] | Case: Grade 5 case report | 1.0 mg/kg EOW | 12 | 1 | Proteinuria, EF, sweat function |
|  | Hirashio et al. 2009  [89] | Case: Grade 5 case report | 1.0 mg/kg EOW | 30 | 1 | Renal GL-3, pain |
|  | Imbriaco et al. 2010  [90] | Case: Grade 5 case report | 1.0 mg/kg EOW | 72 | 1 | LVM, EF |
|  | Moura et al. 2013  [91] | Case: Grade 5 case report | 0.8–1.6 mg/kg EOW | 30 | 1 | Plasma GL-3, LVM, LVWT, hearing |
|  | Mukdsi et al. 2012  [92] | Case: Grade 5 case report | 90 mg EOW | 36 | 1 | Proteinuria |
|  | Schiffmann et al. 2006  [93] | Case: Grade 5 case report | 1.0 mg/kg EOW | 30 | 1 | Vascular endothelial GL-3 |
|  | Togawa et al. 2010  [94] | Case: Grade 5 case report^b^ | 1.0 mg/kg EOW | 48 | 1 | Plasma GL-3,  plasma lyso-GL-3 |
|  | Trimarchi et al. 2013  [95] | Case: Grade 5 case report | 1.0 mg/kg EOW | 1 | 1 | Proteinuria |
|  | Trimarchi et al. 2014  [96] | Case: Grade 5 case report | Agalsidase beta dose NS | 24 | 1 | Proteinuria |
|  | Tsambaos et al. 2004  [97] | Case: Grade 5 case report | 1.0 mg/kg EOW | 12 | 1 | Sweat gland GL-3, sweat function, PNS nerve sensitivity, pain, GI outcomes, QoL |
|  | Tsuboi et al. 2007  [98] | Case: Grade 4 case series | 1.0 mg/kg EOW | NR | 5 | Plasma GL-3, QoL |
|  | Yamadera et al. 2009  [99] | Case: Grade 5 case report | 1.0 mg/kg EOW | 12 | 1 | Urinary GL-3, WMH |
| **Agalsidase alfa and agalsidase beta comparison** | Ghali et al. 2012  [100] | Observational: Grade 3 retrospective observational study | Alfa: NS Beta: 0.3 mg/kg EOW Beta: 1.0 mg/kg EOW | Regular dose: 73.5 Low-dose: 18 | 32 | Sweat function, PNS nerve sensitivity, pain, GI outcomes, QoL |
|  | Rombach et al. 2012  [101] | Observational: Grade 2 prospective observational study | Alfa: 0.2 mg/kg EOW Beta: 0.2–1.0 mg/kg EOW | >12 | 29 | Plasma GL-3,  plasma lyso-GL-3,  urinary GL-3, LVM |
|  | van Breemen et al. 2011 [102] | Observational: Grade 3 retrospective observational study | Alfa: 0.2 mg/kg EOW Beta: 0.2 mg/kg EOW Beta: 1.0 mg/kg EOW | 12 | 22 | Plasma GL-3,  plasma lyso-GL-3 |
| **Treatment not specified or combination of agalsidase alfa and agalsidase beta  (ERT mixed)** | Anderson et al. 2014  [103] | Observational: Grade 3 retrospective observational study | Mixed  Alfa: 0.2 mg/kg EOW Beta: 1.0 mg/kg EOW | ≤116 | 109 | eGFR, proteinuria, LVM |
|  | Fujii et al. 2012  [104] | Observational: Grade 2 prospective observational study | Mixed  Alfa: 0.2 mg/kg EOW Beta: 1.0 mg/kg EOW | 12 | 2 | eGFR, LVM |
|  | Komori et al. 2013  [105] | Observational: Grade 2 prospective observational study | ERT NS | 46.6 (8–90) | 8 | Hearing |
|  | Lavoie et al. 2013  [106] | Observational: Grade 3 retrospective observational study | Mixed  Alfa: 0.2 mg/kg EOW Beta: 1.0 mg/kg EOW | 30 | 49 | Urinary lyso-GL-3 |
|  | Lenders et al. 2016  [107] | Observational: Grade 3 retrospective observational study | Mixed  Alfa: 0.2 mg/kg EOW Beta: 1.0 mg/kg EOW | ERTi−:  58.7 ± 43.1 ERTi+:  86.2 ± 45.4 | 24 | eGFR, proteinuria, LVM, hearing, pain, GI outcomes |
|  | Lenders et al. 2016  [108] | Observational: Grade 3 retrospective observational study | Alfa: NS  Beta: NS | 81 ± 24 | 26 | eGFR, TIA/stroke |
|  | Lin et al. 2013  [109] | Observational: Grade 3 retrospective observational study | Mixed  Alfa: 0.2 mg/kg EOW Beta: 1.0 mg/kg EOW | 17 ± 8.8  (6–39) | 19 | Plasma lyso-GL-3, eGFR, albuminuria, LVM, LVWT |
|  | Liu et al. 2014  [110] | Observational: Grade 3 retrospective observational study | Mixed  Alfa: 0.2 mg/kg EOW Beta: 1.0 mg/kg EOW | 13–46 | 18 | Plasma lyso-GL-3, LVM |
|  | Nicholls et al. 2012  [111] | Observational: Grade 2 prospective observational study | NS | ≤108 | 33 | TIA/stroke |
|  | Rombach et al. 2013  [112] | Observational: Grade 2 prospective observational study | Mixed  Alfa: 0.2 mg/kg EOW Beta: 0.2–1.0 mg/kg EOW | 66 | 27 | eGFR, LVM, WMH, TIA/stroke |
|  | Schmied et al. 2016  [113] | Observational: Grade 3 retrospective observational study | ERT NS | 79.2 | 25 | LVM, LVWT, EF, ECG |
|  | Sirrs et al. 2014  [114] | Observational: Grade 2 prospective observational study | Mixed  Alfa: 0.2 mg/kg EOW Beta: 1.0 mg/kg EOW | Prior ERT: 64 No prior ERT: 59 | 100 | eGFR, LVM, TIA/stroke |
|  | Suntjens et al. 2015  [115] | Observational: Grade 3 retrospective observational study | Mixed  Alfa: 0.2 mg/kg EOW Beta: 0.2–1.0 mg/kg EOW | Classic: 72 Later-onset: 36 | 35 | Hearing |
|  | Talbot et al. 2015  [116] | Observational: Grade 3 retrospective observational study | ERT NS | 120 | 25 | eGFR, LVM, ECG |
|  | Üçeyler et al. 2011  [117] | Observational: Grade 2 prospective observational study | Mixed  Alfa: 0.2 mg/kg EOW Beta: 1.0 mg/kg EOW | 0.1–103 | 18 | PNS nerve sensitivity, QoL (depression score) |
|  | Arends et al. 2016  [84] | Case: Grade 5 case report^c^ | Mixed Alfa: 0.2 mg/kg EOW  (1 year) Beta: 1.0 mg/kg EOW (4 years) | 132 | 1 | eGFR, LVM, WMH, pain |
|  | Di Lazzaro et al. 2013  [118] | Case: Grade 5 case report | ERT NS | 72 | 1 | TIA/stroke |
|  | Iemolo et al. 2014  [119] | Case: Grade 5 case report^c^ | ERT NS | NR | 1 | Pain |
|  | Kikumoto et al. 2010  [120] | Case: Grade 4 case series | ERT NS | 72 or 84 | 2 | Plasma GL-3, TIA/stroke |
|  | Kim et al. 2015  [121] | Case: Grade 5 case report | ERT NS | 12 | 1 | Proteinuria |
|  | Komamura et al. 2004  [122] | Case: Grade 5 case report | 0.2 mg/kg EOW | 6 | 1 | LVM, LVWT, EF |
|  | Korsholm et al. 2015  [123] | Case: Grade 4 case series | Switch  Alfa: 0.2 mg/kg EOW Beta: 1.0 mg/kg EOW | ≤108 | 12 | WMH, TIA/stroke |
|  | Maixnerová et al. 2013  [124] | Case: Grade 5 case report^c^ | ERT NS | 120 | 1 | LVM, pain |
|  | Politei et al. 2016  [125] | Case: Grade 4 case series | Alfa: 0.2 mg/kg EOW Beta: 1.0 mg/kg EOW | 34–144 | 8 | eGFR, proteinuria, LVM, pain |
|  | Qian et al. 2015  [126] | Case: Grade 5 case report | Alfa: 0.2 mg/kg EOW Beta: 0.2–1.0 mg/kg EOW | >156 | 1 | LVWT |
|  | Rigoldi et al. 2014  [127] | Case: Grade 4 case series | Alfa: 0.2 mg/kg EOW Beta: 1.0 mg/kg EOW | 60–120 | 11 | LVM, sweat function, TIA/stroke, pain, GI outcomes |
|  | Saarinen et al. 2015  [128] | Case: Grade 5 case report | ERT NS | NR | 1 | TIA/stroke |
|  | Shen et al. 2007  [129] | Case: Grade 5 case report | Alfa: 10.5 mg EOW Beta: 70 mg EOW | Alfa: 14 Beta: 29 | 1 | Sweat function, heat intolerance, pain |
|  | Skrunes et al. 2017  [130] | Case: Grade 4 case series | ERT mixed  (different regimens) | 96–120 | 3 | Renal GL-3, eGFR, proteinuria, pain, GI outcomes |
|  | Suzuki et al. 2011  [131] | Case: Grade 5 case report | Alfa: 0.2 mg/kg EOW Beta: 1.0 mg/kg EOW | 120 | 1 | eGFR |
|  | Tesmoingt et al. 2009  [132] | Case: Grade 5 case report | Alfa: 0.2 mg/kg EOW Beta: 0.5–1.0 mg/kg EOW | Alfa: 32 Beta: 5 | 1 | eGFR |
|  | Tsuboi et al. 2014  [133] | Case: Grade 5 case report^c^ | Alfa: 0.2 mg/kg EOW Beta: 0.7–1.0 mg/kg EOW | Beta 1.0 mg/kg, EOW: 27 Beta 0.7 mg/kg, EOW: 10 Alfa 0.2 mg/kg, EOW: 28 | 1 | Plasma GL-3,  plasma lyso-GL-3 |
| ^a^ Duration as reported in the publication. ^b^ This was a Grade 2 prospective observational study, but data available at baseline and follow-up for only one patient, so classified as a Grade 5 case report. ^c^ These were Grade 4 case series, but data available at baseline and follow-up for only one patient, so classified as Grade 5 case report. CNS, central nervous system; ECG, electrocardiography; EF, ejection fraction; eGFR, estimated glomerular filtration rate; EOW, every other week; ERT, enzyme replacement therapy; ERTi−, enzyme replacement inhibition negative; ERTi+, enzyme replacement inhibition positive; GI, gastrointestinal; GL-3, globotriaosylceramide; LVM, left ventricular mass; LVMi, left ventricular mass index; LVWT, left ventricular wall thickness; lyso-GL-3, globotriaosylsphingosine; NR, not reported; NS, not specified; NYHA, New York Heart Association; PNS, peripheral nervous system; QoL, quality of life; TIA, transient ischaemic attack; WMH, white matter lesions. | | | | | | |
|  | | | | | | |
|  | | | | | | |

# Supplementary Table 1b Publications describing clinical outcomes with ERT in mixed-gender studies (populations including ≥50% male patients with Fabry disease)

| **Treatment** | **Study, year [reference]** | **Evidence Grade** | **Dose** | **Duration^a^ (months)** | **Total N^b^  (ERT-treated)** | **Percentage male** | **Clinical outcomes reported** |
| --- | --- | --- | --- | --- | --- | --- | --- |
| **Agalsidase alfa** | Goláň et al. 2015 [27] | Clinical: Grade 1a randomized controlled trial | 0.2 mg/kg EOW 0.2 mg/kg weekly 0.4 mg/kg weekly | 12 | 44 | 59 | Plasma GL-3, eGFR, LVM, exercise testing, QoL |
|  | Hajioff et al. 2003 [134] | Clinical: Grade 1a/c | 0.2 mg/kg EOW | 6─42 | 25 | 92 | Hearing |
|  | Hughes et al. 2013 [135] | Clinical: Grade 1a randomized controlled trial | 0.2 mg/kg EOW 0.1 mg/kg weekly 0.2 mg/kg weekly | 3 | 19 | 68 | Plasma GL-3, urinary GL-3, sweat function, pain, QoL |
|  | Jardim et al. 2006 [136] | Clinical: Grade 1c single-arm clinical trial | 0.2 mg/kg EOW | 24 | 8 | 87 | eGFR, proteinuria, neurological examination, WMH, TIA/stroke |
|  | Pastores et al. 2007 [35] | Clinical: Grade 1c single-arm clinical trial | 0.2 mg/kg EOW | 10.5 (median) | 22 | 91 | eGFR |
|  | Beck et al. 2004 [137] | Observational: Grade 3 retrospective observational study | 0.2 mg/kg EOW | 17 (mean) | 314 | 52 | eGFR, LVM, LVWT, TIA/stroke, pain |
|  | Conti et al. 2003 [138] | Observational: Grade 2 prospective observational study | 0.2 mg/kg EOW | NR | 14 | 71 | Hearing |
|  | Cybulla et al. 2009 [139] | Observational: Grade 3 retrospective observational study | 0.2 mg/kg EOW | 42.4 (31.0─56.8) | 20 | 90 | eGFR, proteinuria |
|  | Dehout et al. 2004 [140] | Observational: Grade 2 prospective observational study | 0.2 mg/kg EOW | 6 or 12 | 11 | 82 | GI outcomes |
|  | Dehout et al. 2003 [141] | Observational: Grade 3 retrospective observational study | 0.2 mg/kg EOW | 12 | 234 | NR | eGFR |
|  | Feriozzi et al. 2007  [142] | Observational: Grade 2 prospective observational study | 0.2 mg/kg EOW | 36 or 48 | 34 | 71 | eGFR, proteinuria |
|  | Hsu et al. 2014 [143] | Observational: Grade 3 retrospective observational study | 0.2 mg/kg EOW | 8─40 | 17 | 77 | Cardiac GL-3, LVM |
|  | Mehta et al. 2009 [144] | Observational: Grade 3 retrospective observational study | 0.2 mg/kg EOW | 60 | 181 | 70 | eGFR, LVM, TIA/stroke, pain, QoL |
|  | Palla et al. 2007  [145] | Observational: Grade 2 prospective observational study | 0.2 mg/kg EOW | ≤60 | 24 | 63 | Hearing |
|  | Schwarting et al. 2006 [146] | Observational: Grade 3 retrospective observational study | 0.2 mg/kg EOW | 1─40 | 201 | 63 | eGFR |
|  | Thofehrn et al. 2009 [147] | Observational: Grade 2 prospective observational study | 0.2 mg/kg EOW | 12─36 | 9 | 78 | eGFR, proteinuria, TIA/stroke |
| **Agalsidase beta** | Banikazemi et al. 2007 [148] | Clinical: Grade 1a randomized controlled trial | 1.0 mg/kg EOW | 0─35 | 51 | 88 | TIA/stroke, clinical events |
|  | Eng et al. 2001 [149] | Clinical: Grade 1a/c | 1.0 mg/kg EOW | 6–11 | 58 | 97 | Plasma GL-3, renal GL-3, cardiac GL-3, dermal GL-3, eGFR, ECG, pain, QoL |
|  | Germain et al. 2007 [150] | Clinical: Grade 1c single-arm clinical trial | 1.0 mg/kg EOW | 54 | 58 | 97 | Renal GL-3, cardiac GL-3, dermal GL-3, eGFR, TIA/stroke, pain, QoL |
|  | Pisani et al. 2005 [151] | Clinical: Grade 1c single-arm clinical trial | 1.0 mg/kg EOW | 24 | 9 | 89 | LVM, EF, pain, GI outcomes |
|  | Tahir et al. 2007 [152] | Clinical: Grade 1c single-arm clinical trial | 1.0 mg/kg EOW | 6─43 | 11 | 73 | eGFR, proteinuria |
|  | Thurberg et al. 2002 [153] | Clinical: Grade 1a/c | 1.0 mg/kg EOW | 6─11 | 58 | 97 | Renal GL-3 |
|  | Thurberg et al. 2004 [154] | Clinical: Grade 1c single-arm clinical trial | 1.0 mg/kg EOW | 30─36 | 58 | 97 | Dermal GL-3 |
|  | Thurberg et al. 2009 [155] | Clinical: Grade 1c single-arm clinical trial | 1.0 mg/kg EOW | 53 or 59 | 58 | 97 | Cardiac GL-3 |
|  | Weidemann et al. 2003 [156] | Clinical: Grade 1c single-arm clinical trial | 1.0 mg/kg EOW | 12 | 16 | 87 | LVWT, EF, exercise testing |
|  | Wilcox et al. 2004 [157] | Clinical: Grade 1c single-arm clinical trial | 1.0 mg/kg EOW | 30 | 58 | 97 | Plasma GL-3, dermal GL-3, eGFR, proteinuria, TIA/stroke, pain, QoL |
|  | Beer et al. 2006 [158] | Observational: Grade 2 prospective observational study | 1.0 mg/kg EOW | 12 | 17 | 57 | LVM |
|  | Breunig et al. 2006 [159] | Observational: Grade 2 prospective observational study | 1.0 mg/kg EOW | 12─37 | 26 | 77 | eGFR, proteinuria, LVM, LVWT,  exercise testing, TIA/stroke |
|  | Choi et al. 2008 [160] | Observational: Grade 2 prospective observational study | 1.0 mg/kg EOW | 4─27 | 11 | 73 | Plasma GL-3, urinary GL-3, renal GL-3, proteinuria, LVM, ECG, hearing |
|  | Collin et al. 2012 [161] | Observational: Grade 2 prospective observational study | 1.0 mg/kg EOW | 54 | 30 | 94 | LVM, EF |
|  | Fellgiebel et al. 2014 [162] | Observational: Grade 3 retrospective observational study | 1.0 mg/kg EOW | 27 (median) | 25 | 93 | WMH |
|  | Germain et al. 2015 [163] | Observational: Grade 3 retrospective observational study | 1.0 mg/kg EOW | 120 (median) | 52 | 96 | Plasma GL-3, eGFR, LVWT, TIA/stroke, clinical events |
|  | Imbriaco et al. 2009 [164] | Observational: Grade 2 prospective observational study | 1.0 mg/kg EOW | 29─58 | 11 | 73 | Proteinuria, LVM, LVWT, EF, sweat function, GI outcomes, pain |
|  | Juan et al. 2014 [165] | Observational: Grade 2 prospective observational study | 1.0 mg/kg EOW | ≥120 | 6 | 67 | eGFR, proteinuria, albuminuria, LVM, CNS neurological examinations, QoL |
|  | Kalliokoski et al. 2006 [166] | Observational: Grade 2 prospective observational study | 1.0 mg/kg EOW | NR | 10 | 70 | Plasma GL-3, LVM, LVWT, EF |
|  | Kim et al. 2016 [79] | Observational: Grade 2 prospective observational study | 1.0 mg/kg EOW | 60–126 | 19 | 79 | Plasma GL-3, urinary GL-3 |
|  | Koeppe et al. 2012 [167] | Observational: Grade 2 prospective observational study | 1.0 mg/kg EOW | 13 | 25 | 84 | LVM, LVWT, EF |
|  | Koskenvuo et al. 2008 [168] | Observational: Grade 2 prospective observational study | 1.0 mg/kg EOW | 24 | 9 | 56 | Plasma GL-3, EF, ECG, exercise testing, pain, QoL |
|  | Machann et al. 2011 [169] | Observational: Grade 2 prospective observational study | 1.0 mg/kg EOW | 14 | 23 | 57 | LVM |
|  | Messalli et al. 2012 [170] | Observational: Grade 2 prospective observational study | 1.0 mg/kg EOW | 48 | 16 | 63 | LVM, LVWT, EF, cardiac function (NYHA) |
|  | Niemann et al. 2011 [171] | Observational: Grade 2 prospective observational study | 1.0 mg/kg EOW | 35 ± 23 | 56 | 57 | LVWT, EF |
|  | Ortiz et al. 2016  [172] | Observational: Grade 3 retrospective observational study | 1.0 mg/kg EOW | 60 | 1,044 | 61 | Clinical events |
|  | Spinelli et al. 2004 [173] | Observational: Grade 2 prospective observational study | 1.0 mg/kg EOW | 12 | 9 | 78 | LVM, LVWT, EF |
|  | Weidemann et al. 2009 [174] | Observational: Grade 2 prospective observational study | 1.0 mg/kg EOW | 36 | 32 | 82 | LVM, LVWT, EF, exercise testing |
|  | Weidemann et al. 2013 [175] | Observational: Grade 2 prospective observational study | 1.0 mg/kg EOW | ≥60 | 40 | 78 | eGFR, proteinuria, EF, sweat function, TIA/stroke, pain |
| **Agalsidase alfa and agalsidase beta comparison** | Vedder et al. 2007 [176] | Clinical: Grade 1a randomized controlled trial | Alfa: 0.2 mg/kg EOW Beta: 0.2 mg/kg EOW | ≥12 | 34 | 53 | Plasma GL-3, urinary GL-3, eGFR, proteinuria, LVM, pain |
|  | Ghali et al. 2012 [100] | Observational: Grade 3 retrospective observational study | Alfa: NR Beta: 0.5 mg/kg EOW Beta: 1.0 mg/kg EOW | 28─150 | 40 | 82 | Sweat function, PNS nerve sensitivity, pain, GI outcomes, QoL |
|  | Lenders et al. 2016 [177] | Observational: Grade 3 retrospective observational study | Alfa: 0.2 mg/kg EOW Beta: 0.3–0.5 mg/kg EOW Beta: 1.0 mg/kg EOW | 24 | 89 | 63 | eGFR, EF, TIA/stroke, pain, GI outcomes |
|  | Linthorst et al. 2004 [178] | Observational: Grade 2 prospective observational study | Alfa: 0.2 mg/kg EOW Beta: 0.2 mg/kg EOW Beta: 1.0 mg/kg EOW | 6─12 | 18 | 89 | Urinary GL-3 |
|  | Pisani et al. 2013 [179] | Observational: Grade 2 prospective observational study | Alfa: 0.2 mg/kg EOW Beta: 1.0 mg/kg EOW | Beta: ≥48 pre-switch Alfa: 20 | 10 | 70 | eGFR, proteinuria, LVM, LVWT, EF |
|  | Vedder et al. 2008 [180] | Observational: Grade 2 prospective observational study | Alfa: 0.2 mg/kg EOW Beta: 0.2 mg/kg EOW Beta: 1.0 mg/kg EOW | ≥12 | 52 | 54 | Plasma GL-3, urinary GL-3, eGFR, LVM |
|  | Weidemann et al. 2014 [181] | Observational: Grade 2 prospective observational study | Alfa: 0.2 mg/kg EOW Beta: 0.3-0.5 mg/kg EOW Beta: 1.0 mg/kg EOW | 8─16 | 105 | 59 | eGFR, albuminuria, LVWT, EF, PNS nerve sensitivity, TIA/stroke, pain, GI outcomes, QoL |
| **Treatment not specified/ combination of agalsidase alfa and agalsidase beta  (ERT mixed)** | Goker-Alpan et al. 2015 [26] | Clinical: Grade 1c single-arm clinical trial | Alfa: 0.2 mg/kg EOW  (pre-switch: alfa or beta) | 24 | 100 | 54 | eGFR, LVM, TIA/stroke |
|  | Anderson et al. 2014 [103] | Observational: Grade 3 retrospective observational study | Mixed  Alfa: 0.2 mg/kg EOW Beta: 1.0 mg/kg EOW | ≤116 | 109 | 52 | LVM, pain, GFR, proteinuria |
|  | Chen et al. 2016 [182] | Observational: Grade 3 retrospective observational study | Mixed Alfa: 0.2 mg/kg EOW Beta: 1.0 mg/kg EOW | 12 | 25 | 88 | Plasma lyso-GL-3, LVM, LVWT (IVS) |
|  | Engelen et al. 2012 [183] | Observational: Grade 3 retrospective observational study | Mixed Alfa: 0.2 mg/kg EOW Beta: 1.0 mg/kg EOW | 20─71 | 40 | 53 | LVM, EF, ECG |
|  | Kovacevic-Preradovic et al. 2008 [184] | Observational: Grade 2 prospective observational study | Mixed Alfa: 0.2 mg/kg EOW Beta: 1.0 mg/kg EOW | 20─55 | 29 | 69 | LVWT, exercise testing, pain |
|  | Krämer et al. 2014 [185] | Observational: Grade 2 prospective observational study | ERT NS | 57.6 ± 28.8 | 57 | 52 | LVM, LVWT, EF |
|  | Mignani et al. 2008 [186] | Observational: Grade 2 prospective observational study | Mixed Alfa: 0.2 mg/kg EOW Beta: 1.0 mg/kg EOW | Dialysis: 45.1 (19.8) Transplant: 48.4 (13.2) | 34 | 94 | Plasma GL-3, urinary GL-3, proteinuria, LVM, LVWT, TIA/stroke |
|  | Niemann et al. 2010 [187] | Observational: Grade 2 prospective observational study | ERT NS | 41 ± 23 | 57 | 75 | LVWT |
|  | Pisani et al. 2014 [188] | Observational: Grade 2 prospective observational study | Mixed Alfa: 0.2 mg/kg EOW Beta: 1.0 mg/kg EOW | ≥12 | 6 | 53 | eGFR, proteinuria |

^a^ Duration as reported in the publication. ^b^ N = number of patients in the ERT-treated mixed-gender population.
CNS, central nervous system; ECG, electrocardiography; EF, ejection fraction; eGFR, estimated glomerular filtration rate; EOW, every other week; ERT, enzyme replacement therapy; GI, gastrointestinal; GL-3, globotriaosylceramide; IVS, intraventricular septum; LVM, left ventricular mass; LVWT, left ventricular wall thickness; lyso-GL-3, globotriaosylsphingosine; NR, not reported; NS, not specified; NYHA, New York Heart Association; PNS, peripheral nervous system; QoL, quality of life;
TIA, transient ischaemic attack; WMH, white matter lesions.

# Supplementary Table 2 Plasma GL-3 outcomes with approved doses of agalsidase alfa and agalsidase beta in adult male patients

|  | **Study, year [reference]**  **(number of patients^a^)**  ***Evidence grade*^b^** | **Male,**  **n (%)^c^** | **Duration (months)** | **Units** | **Baseline**  **(number of patients^d^)** | **End-point**  **(number of patients^e^)** | **Overall result**  **(p value/95% CI)** |
| --- | --- | --- | --- | --- | --- | --- | --- |
| **Alfa** | Clarke et al. 2007 [25] (N = 18)  *Grade 1a* | 18 (100) | 2.5 | nmol/mL (mean (range) or mean [SD]) | 9.72 (4.8–4.4)  (n = 4) | Significant reduction of about 50% | **↓ (p < 0.001)** |
|  | Goker-Alpan et al. 2015  [26] (N = 132)  *Grade 1c*  *No detailed information on prior dosing available* | 81 (61) | 24 | nmol/mL  (mean ± SE)  (normal: 4.55 ± 3.9 (1.96–7.7) in healthy volunteers) | ERT-naïve group:  19.02 ± 3.11  (n = 10)  Switch group:  13.36 ± 0.86  (n = 37) | Trend towards reduction from baseline  (n = 3)  NC  (n = 18) | ↓ (NR)  NC |
|  | Goláň et al. 2015  [27] (N = 44)  *Grade 1a* | 26 (59) | 12 | nmol/mL  (mean) | 7.2  (n = 14) | 5.5  (n = 11) | ↓ (95% CI −3.36, 0.13) |
|  | Hughes et al. 2008  [29] (N = 7)  *Grade 1a/c* | 7 (100) | 30 | nmol/mL  (mean ± SE) | NR  (n = 7) | Decrease  (n = 7) | ↓ (NR) |
|  | Pastores et al. 2007  [35] (N = 22)  *Grade 1c* | 20 (91) | Range:  2.5–15 | nmol/mL  (mean ± SE) | 4.76 ± 0.47  (n = 20) | 12 months: 3.40 ± 0.53  (n = 5) | **↓ (0.017)** |
|  | Schiffmann et al. 2001  [36] (N = 14)  *Grade 1a* | 14 (100) | 5.5 | nmol/mL  (mean ± SE) | 12.14 ± 0.907  (n = 14) | 5.58 ± 0.536  (n = 14) | ↓ (NR) |
|  | Schiffmann et al. 2006  [39] (N = 25)  *Grade 1c* | 25 (100) | 48–54 | nmol/mL  (mean ± SE) | 11.4 ± 0.8  (n = 25) | Month 48:  5.0 ± 0.6  (n = 25) | **↓ (p < 0.001)** |
|  | Schiffmann et al. 2007  [52] (N = 12)  *Grade 2* | 12 (100) | 24–48 | nmol/mL | 11.8 [3.6]  (n = 12) | 4.2 [1.6]  (n = 11) | **↓ (p < 0.05)** |
| **Beta** | Bénichou et al. 2009  [75] (N = 134)  *Grade 3* | 122 (91) | Range: 1.9–60.7 | µg/mL  (normal: >7.03 µg/mL) | Above normal  (>7.03) in 80% pts  (n = 122) | Levels remained normal (normalized by 6–12 months)  (n = 122) | ↓ (NR)  Normalized |
|  | Elliott et al. 2006  [76] (N = 5)  *Grade 2* | 5 (100) | 10.1 [2.3] | µg/mL | NR  (n = 5) | −5.3  (n = 5) | **↓ (p = 0.04;  95% CI 0.5, 10.1)** |
|  | Eng et al. 2001  [69] (N = 15)  *Grade 1c* | 15 (100) | 5 infusions | ng/µL  (normal: <1.2) | 17.1 [12.8]  (n = 15) | 2/3 clearance  1/3 reduction  (n = 3) | ↓ (NR) |
|  | Eto et al. 2005  [70] (N = 13)  *Grade 1c* | 13 (100) | 4.6 | ng/µL | 3.9 [2.7]  (n = 13) | 0.2 [0.8]  (n = 13) | ↓ **(p < 0.001)** |
|  | Goker-Alpan et al. 2016  [71] (N = 15)  *Grade 1c*  *Pre-treatment with agalsidase alfa (median treatment duration), years (range): 3.7 (1.6–14)* | 15 (100) | 6 | Absolute change, µg/mL  Change, % | NR  (n = 15)  NR  (n = 15) | −0.9  (n = 14)  −17.9  (n = 14) | **↓ (p < 0.05)**  **↓ (p < 0.05)** |
|  | Lubanda et al. 2009  [72] (N = 21)  *Grade 1c* | 21 (100) | 6^f^ | µg/mL  (normal: ≤7.0) | 12.2 [3.4]  (n = 21) | 5.9 [1.1]  (n = 21) | ↓ **(p < 0.001)**  **normalized** |
|  | Mignani et al. 2004  [80] (N = 3)  *Grade 2* | 3 (100) | 18 | ng/µL | 3.16–8.27 | 1.57–4.94 | ↓ (NR) |
| **Alfa** | van Breemen et al. 2011 [102] (N = 43)  *Grade 3* | 22 (51) | 12 | µM (normal: <3.18) | Alfa 0.2 Ab+:  7.03 (5.95–8.80)  (n = 7)  Beta 1.0 Ab+:  6.56 (3.97–8.74)  (n = 9) | Alfa 0.2 Ab+:  3.74 (3.07–6.42)  (n = 7)  Beta 1.0 Ab+:  2.77 (1.34–3.37)  (n = 9) | **↓ (p < 0.01)**  **normalized**  **↓ (p < 0.01)** |
| **Beta** |  |  |  |  |  |  |  |
| **Alfa** | Rombach et al. 2012  [101] (N = 59)  *Grade 2* | 29 (49)  *Ab+  (n = 14)*  *Ab−  (n = 12)* | ≥12 | µmol/L  (median, range)  (normal:  0.45–2.46) | Alfa 0.2 Ab+:  4.7 (4.0–7.1)  (n = 4)  Beta 1.0 Ab+:  5.3 (3.1–6.1)  (n = 4) | Alfa 0.2 Ab+:  3.6 (3.5–4.1)  (n = 4)  Beta 1.0 Ab+:  2.0 (1.9–2.1)  (n = 4) | ↓ (NR)  Normalized  ↓ (NR) |
| **Beta** |  |  |  |  |  |  |  |

Data are means [SD] or means ± SE or medians (range), unless otherwise indicated. Red font indicates statistically significant changes.

Case series, case reports, mixed-ERT publications, paediatric–adult mixed publications, and publications with other dose regimens are not included.

^a^ Total number of patients included in the study who were treated with ERT. ^b^ Study grades defined as follows: Grade 1a, randomized controlled trial; Grade 1c, single-arm clinical trial; Grade 1a/c, randomized controlled trial with single-arm open-label extension; Grade 2, prospective observational study; Grade 3, retrospective observational study. ^c^ Number of male patients who were treated with ERT. ^d^ Number of male, ERT-treated patients with data for the outcome at baseline. ^e^ Number of male, ERT-treated patients with data for the outcome at endpoint. ^f^ 6 months at normal dose (1.0 mg/kg EOW) followed by 18 months on reduced dose (0.3 mg/kg EOW). Details of dose under dose change section.

↓, decrease; ↑, increase; Ab+, antibody-positive; Ab−, antibody-negative; CI, confidence interval; EOW, every other week; ERT, enzyme replacement therapy; GL-3, globotriaosylceramide; NC, no change; NR, not reported; NS, not significant; pt, patient; SD, standard deviation; SE, standard error.

# Supplementary Table 3 Plasma lyso-GL-3 outcomes with approved doses of agalsidase alfa and agalsidase beta in adult male patients

|  | **Study, year [reference]**  **(number of patients^a^)**  ***Evidence grade*^b^** | **Male,**  **n (%)^c^** | **Duration (months)** | **Units** | **Baseline**  **(number of patients^d^)** | **End-point**  **(number of patients^e^)** | **Overall result**  **(p value)** |
| --- | --- | --- | --- | --- | --- | --- | --- |
| **Alfa** | Goker-Alpan et al. 2015  [26] (N = 132)  *No detailed information on prior dosing available*  *Grade 1c* | 81 (61) | 24 | nM | ERT-naïve:  102.67 ± 19.09  (n = 10)  Switch:  57.94 ± 5.11  (n = 37) | Significant reduction from baseline  (n = 3)  NC  (n = 18) | **↓ (NR)**  NC (NR) |
| **Beta** | Goker-Alpan et al. 2016  [71] (N = 15)  *Grade 1c*  *Pre-treatment with agalsidase alfa (median treatment duration), years (range): 3.7 (1.6–14)* | 15 (100) | 6 | Absolute change, µg/mL | NR  (n = 14) | −16.7  (n = 14) | **↓ (p < 0.001)** |
|  |  |  |  | Reduction, % | NR  (n = 14) | 39.5% [23.57]  (n = 14) | **↓ (p < 0.001)** |
| **Alfa** | Rombach et al. 2012  [101] (N = 59)  *Grade 2* | 29 (49) | ≥12 | nmol/L  (normal: 0.3−0.5 in healthy controls) | Alfa 0.2 Ab+:  91 (70–119)  (n = 4)  Beta 1.0 Ab+:  76 (53–124)  (n = 4) | Alfa 0.2 Ab+:  65 (38–80)  (n = 4)  Beta 1.0 Ab+:  17.5 (15–23)  (n = 4) | ↓ (NR)  ↓ (NR) |
| **Beta** |  |  |  |  |  |  |  |
| **Alfa** | van Breemen et al. 2011 [102] (N = 43)  *Grade 3* | 22 (51) | 12 | nM  (normal: <3) | Alfa 0.2 Ab+:  220 (148–250)  (n = 7)  Beta 1.0 Ab+:  189 (134–397)  (n = 9) | Alfa 0.2 Ab+:  118 (52–149)  (n = 7)  Beta 1.0 Ab+:  55 (23–113)  (n = 9) | ↓ (NR)  ↓ (NR) |
| **Beta** |  |  |  |  |  |  |  |

Data are means [SD] or means ± SE or medians (range), unless otherwise indicated. Red font indicates statistically significant changes.

Case series, case reports, mixed-ERT publications, paediatric–adult mixed publications, and publications with other dose regimens are not included.

^a^ Total number of patients included in the study who were treated with ERT. ^b^ Study grades defined as follows: Grade 1a, randomized controlled trial; Grade 1c, single-arm clinical trial; Grade 2, prospective observational study; Grade 3, retrospective observational study. ^c^ Number of male patients who were treated with ERT. ^d^ Number of male, ERT-treated patients with data for the outcome at baseline. ^e^ Number of male, ERT-treated patients with data for the outcome at endpoint.

↓, decrease in levels; ↑, increase in levels; Ab+, antibody-positive; ERT, enzyme replacement therapy; lyso-GL-3, globotriaosylsphingosine; NC, no change; NR, not reported; NS, not significant; SD, standard deviation; SE, standard error.

# **Supplementary Table 4** Urinary GL-3 outcomes with approved doses of agalsidase alfa and agalsidase beta in adult male patients

|  | **Study, year [reference]**  **(number of patients^a^)**  ***Evidence grade*^b^** | **Male, n (%)^c^** | **Duration (months)** | **Units** | **Baseline**  **(number of patients^d^)** | **End-point**  **(number of patients^e^)** | **Overall result (p value)** |
| --- | --- | --- | --- | --- | --- | --- | --- |
| **Alfa** | Goker-Alpan et al. 2015  [26] (N = 132)  *Grade 1c*  *No detailed information on prior dosing available* | 81 (61) | 24 | nmol/mg,  creatinine-normalized | ERT-naïve group:  3.78 ± 0.83  (n = 10) | Decrease  (n = 3) | ↓ (NS) |
|  |  |  |  |  | Switch group:  2.74 ± 0.51  (n = 36) | Increase  (n = 18) | **↑** (NS) |
|  | Hughes et al. 2008  [29] (N =7)  *Grade 1a* | 7 (100) | 6 | nmol/24 h | NR | Decrease | ↓ (NR) |
|  | Pastores et al. 2007  [35] (N = 22)  *Grade 1c* | 20 (91) | Range:  2.5–15 | nmol/g creatinine | ≤33 (normal levels)  (n = 11)  117  (n = 1) | NC  (n = 11)  46  (n = 1) | NC  ↓ (NR) |
|  | Schiffmann et al. 2001 [36] (N = 14)  *Grade 1a* | 14 (100) | 5.5 | nmol/g creatinine | 2,369 ± 308  (n = 14) | 1,683 ± 443  (n = 14) | ↓ (NR) |
|  | Schiffmann et al. 2006  [39] (N = 25)  *Grade 1c* | 25 (100) | 48–54 | nmol/g creatinine | 2,566 ± 299  (n = 25) | Month 36: –59.7%  (n = 21) | **↓ (p < 0.001)** |
|  | Schiffmann et al. 2007  [52] (N = 12)  *Grade 2* | 12 (100) | 24–48 | nmol/g creatinine | 2,839 [1,862]  (n = 12) | 1,318 [901]  (n = 12) | **↓ (p < 0.001)** |
|  | Whitfield et al. 2005  [55] (N = 8)  *Grade 2* | 6 (75) | 12 | GL-3/ creatinine ratio, μmol/mmol | Range:  0.01–14.9  (n = 6) | Decrease by 8.7–20.7% (vs baseline)  (n = 6) | ↓ (NR) |
| **Beta** | Eto et al. 2005  [70] (N = 13)  *Grade 1c* | 13 (100) | 4.6 | nmol/filter | 4,085 [2077]  (n = 13) | 2,687 [2,514]  (n = 13) | ↓ (p = 0.2440) |
|  | Goker-Alpan et al. 2016  [71] (N = 15)  *Grade 1c*  *Pre-treatment with agalsidase alfa (median (range): 3.7 (1.6-14 years)* | 15 (100) | 6 | µg/mmol  (median absolute change)  Median % change | NR  (n = 15) | −11.1  (n = 14) | ↓ (NS) |
|  |  |  |  |  | NR  (n = 15) | −33.8%  (n = 14) | ↓ (NS) |
|  | Lubanda et al. 2009  [72] (N = 21)  *Grade 1c* | 21 (100) | 5.5 | µg/mg creatinine | 221 [152]  (n = 21) | 109 [120]  (n = 21) | **↓ (p < 0.001)** |
|  | Mignani et al. 2004  [80] (N = 3)  *Grade 2* | 3 (100) | 18 | ng/µL | ND  (n = 3) | ND  (n = 3) | NC (NR) |
| **Alfa** | Rombach et al. 2012  [101] (N = 59)  *Grade 2* | 29 (49) | ≥12 | nmol/24 h  (normal: 18–90 in healthy controls) | Alfa 0.2 Ab+:  1.786 (1,362–2,778)  (n = 4)  Beta 1.0 Ab+:  1,790 (669–1,812)  (n = 4) | Alfa 0.2 Ab+:  2,442 (1,714–2,720)  (n = 4)  Beta 1.0 Ab+:  824 (212–1,656)  (n = 4) | ↓ (NR)  ↓ (NR) |
| **Beta** |  |  |  |  |  |  |  |

Data are means [SD] or means ± SE or medians (range), unless otherwise indicated. Red font indicates statistically significant changes.

Case series, case reports, mixed-ERT publications, paediatric–adult mixed publications, and publications with other dose regimens are not included.

^a^ Total number of patients included in the study who were treated with ERT. ^b^ Study grades defined as follows: Grade 1a, randomized controlled trial; Grade 1c, single-arm clinical trial; Grade 2, prospective observational study. ^c^ Number of male patients who were treated with ERT. ^d^ Number of male, ERT-treated patients with data for the outcome at baseline. ^e^ Number of male, ERT-treated patients with data for the outcome at endpoint.

↓, decrease; ↑, increase; Ab+, antibody-positive; ERT, enzyme replacement therapy; GL-3, globotriaosylsphingosine; h, hours; NC, no change; ND, not determined; NR, not reported; NS, not significant; SD, standard deviation; SE, standard error.

# Supplementary Table 5 Kidney GL-3 accumulation outcomes with approved doses of agalsidase alfa and agalsidase beta in adult male patients

|  | **Study, year [reference]**  **(number of patients^a^)**  ***Evidence grade*^b^** | **Male, n (%)^c^** | **Duration (months)** | **Units** | **Baseline**  **(number of patients^d^)** | **End-point**  **(number of patients^e^)** | **Overall result (p value)** |
| --- | --- | --- | --- | --- | --- | --- | --- |
| **Alfa** | Schiffmann et al. 2001 [36] (N = 14)  *Grade 1a* | 14 (100) | 5.5 | nmol/mg of tissue | 19.5 ± 1.68  (n = 11) | 15.6 ± 1.6  (n = 11) | ↓ (NR) |
| **Beta** | Lubanda et al. 2009  [72] (N = 21)  *Grade 1c* | 21 (100) | 5.5 | % patients with zero scores | Interstitial capillary endothelial cells:  14  (n = 21)  Glomerular endothelial cells:  26  (n = 19)  Mesangial cells:  16  (n = 19)  Non-capillary endothelial cells:  14  (n = 21)  Interstitial cells:  14  (n = 21)  Distal convoluted tubule and collecting duct cells:  10  (n = 21)  Non-capillary smooth muscle cells:  5  (n = 21)  Podocytes:  0  (n = 19) | Interstitial capillary endothelial cells:  100  (n = 21)  Glomerular endothelial cells:  100  (n = 19)  Mesangial cells:  100  (n = 19)  Non-capillary endothelial cells:  100  (n = 20)  Interstitial cells:  86  (n = 21)  Distal convoluted tubule and collecting duct cells:  52  (n = 21)  Non-capillary smooth muscle cells:  30  (n = 20)  Podocytes:  0  (n = 19) | **↑ (p < 0.001)**  **↑ (p < 0.001)**  **↑ (p < 0.001)**  **↑ (p < 0.001)**  **↑ (p < 0.001)**  **↑ (p = 0.004)**  ↑ (p = 0.06)  NC |
| **Beta** | Najafian et al. 2016  [73] (N = 6)  *Grade 1c* | 6 (100) | 11–12 | μm^3^  ISGFN score  % | Total volume of GL-3 inclusions per podocyte:  NR  (n = 6)  Podocyte volume:  NR  (n = 6)  Podocyte GL-3:  4.0  (n = 6)  Proportion of podocytes/biopsy without GL-3 inclusions:  0 (0–6.3)  (n = 6) | Total volume of GL-3 inclusions per podocyte:  Mean: −73%  (n = 6)  Podocyte volume:  Mean: −63%  (n = 6)  Podocyte GL-3:  n = 3: NC  n = 3, median (range):  0.1 (0.1–0.7)  Proportion of podocytes/biopsy without GL-3 inclusions:  12 (5–27)  (n = 6) | **↓ (p = 0.02)**  **↓ (p = 0.02)**  NC/↓  (p = 0.18)  **↑ (p = 0.008)** |

Data are means [SD] or means ± SE or medians (range), unless otherwise indicated. Red font indicates statistically significant changes.

Case series, case reports, mixed-ERT publications, paediatric–adult mixed publications, and publications with other dose regimens are not included.

^a^ Total number of patients included in the study who were treated with ERT. ^b^ Study grades defined as follows: Grade 1a, randomized controlled trial; Grade 1c, single-arm clinical trial. ^c^ Number of male patients who were treated with ERT. ^d^ Number of male, ERT-treated patients with data for the outcome at baseline;
^e^ Number of male, ERT-treated patients with data for the outcome at endpoint.

↓, decrease; ↑, increase; ERT, enzyme replacement therapy; GL-3, globotriaosylceramide; ISGFN, scoring system for podocyte GL-3 inclusions developed by the International Study Group for Fabry Nephropathy; NC, no change; NR, not reported; SD, standard deviation; SE, standard error.

# **Supplementary Table 6** Cardiac GL-3 accumulation outcomes with approved dose of agalsidase alfa in adult male patients

|  | **Study, year [reference]**  **(number of patients^a^)**  ***Evidence grade*^b^** | **Male, n (%)^c^** | **Duration (months)** | **Units** | **Baseline**  **(number of patients^d^)** | **End-point**  **(number of patients^e^)** | **Overall result (p value/ 95% CI)** |
| --- | --- | --- | --- | --- | --- | --- | --- |
| **Alfa** | Hughes et al. 2008  [29] (N = 7)  *Grade 1a* | 7 (100) | 6 | nmol/μg protein | 0.71 ± 0.18  (n = 7) | 0.58 ± 0.18  (n = 6) | ↓ (NS) |

Data are means [SD] or means ± SE or medians (range), unless otherwise indicated. GL-3 scores were based on microscopic evaluation. A 4-point scoring system was used, ranging from 0 (normal), to 3 (severe involvement). Case series, case reports, mixed-ERT publications, paediatric-–adult- mixed publications, and publications with other dose regimens are not included.

^a^ Total number of patients included in the study who were treated with ERT. ^b^ Study grade: Grade 1a, randomized controlled trial. ^c^ Number of male patients who were treated with ERT. ^d^ Number of male, ERT-treated patients with data for the outcome at baseline; ^e^ Number of male, ERT-treated patients with data for the outcome at endpoint.

↓, decrease in GL-3 score, i.e. improvement, less accumulation of GL-3; CI, confidence interval; ERT, enzyme replacement therapy; GL-3, globotriaosylceramide; NS, not significant; SD, standard deviation; SE, standard error.

# Supplementary Table 7 GL-3 accumulation outcomes in other organs with approved dose of agalsidase beta in adult male patients

|  | **Study, year [reference]**  **(number of patients^a^)**  ***Evidence grade*^b^** | **Male, n (%)^c^** | **Duration (months)** | **Units** | **Baseline**  **(number of patients^d^)** | **End-point**  **(number of patients^e^)** | **Overall result  (p value)** |
| --- | --- | --- | --- | --- | --- | --- | --- |
| **Beta** | Bénichou et al. 2009 [75] (N = 134)  *Grade 3* | 122 (91) | Range: 1.9–60.7 | Number of pts | Zero scores: 2 pts Mild: 2 pts  Moderate: 37 pts  Severe: 15 pts  (n = 56) | Zero scores: 44 pts (at 6 months or after)  Mild scores: 12 pts  When stratified by antibody titre, the largest proportion of non-zero scores was in the high-titre subgroup  (n = 56) | ↓ (NR) |
|  | Eng et al. 2001 [69] (N = 15)  *Grade 1c* | 15 (100) | 5 infusions | ng/mg tissue    GL-3 accumulation score | Skin:  350 [168]  (n = 14, all dosing regimens)  Liver:  176–2,410  (n = 3)  Liver sinusoid endothelial cells:  2.4 [0.74]  (n = 15, all dosing regimens) | Skin:  all reduced  (n = 3)  Liver:  45–185  (n = 3)  Liver sinusoid endothelial cells:  −2.00 [1.00]  (n = 3) | ↓ (NR)  ↓ (NR)  ↓ (NR) |
|  | Lubanda et al. 2009  [72] (N = 21)  *Grade 1c* | 21 (100) | 5.5 | % pts with zero score | Dermal capillary endothelium:  24%  (n = 21) | Dermal capillary endothelium:  95%  (n = 20) | **↑ (p < 0.001)** |

Data are means [SD] or means ± SE or medians (range), unless otherwise indicated. Red font indicates statistically significant changes. Case series, case reports, mixed-ERT publications, paediatric–adult mixed publications, and publications with other dose regimens are not included.

^a^ Total number of patients included in the study who were treated with ERT. ^b^ Study grades defined as follows: Grade 1c single-arm clinical trial; Grade 3, retrospective observational study. ^c^ Number of male patients who were treated with ERT. ^d^ Number of male, ERT-treated patients with data for the outcome at baseline; ^e^ Number of male, ERT-treated patients with data for the outcome at endpoint.

↓, decrease; ERT, enzyme replacement therapy; GL-3, globotriaosylceramide; NR, not reported; pt, patient; SD, standard deviation; SE, standard error.

# Supplementary Table 8 Proteinuria outcomes with approved doses of agalsidase alfa and agalsidase beta in adult male patients

|  | **Study, year [reference]**  **(number of patients^a^)**  ***Evidence grade*^b^** | **Male, n (%)^c^** | **Duration (months)** | **Units** | **Baseline**  **(number of patient^d^)** | **End-point**  **(number of patients^e^)** | **Overall result (p value)** |
| --- | --- | --- | --- | --- | --- | --- | --- |
| **Alfa** | Feriozzi et al. 2009  [43] (N = 165)  *Grade 3* | 115 (70) | 36 | mg/24 h | 415.4 [417.1]  (n = 115) | 481.0 [394.1]  (n = 115) | NC (p = 0.39) |
|  | Feriozzi et al. 2012  [44] (N = 208)  *Grade 3* | 134 (64) | 60–134 | mg/24 h | Total: 590.6 [832.2]  (n = 84) | Total: 772.6 [1,190.1]  (n = 84) | ↑ (p = 0.06) |
|  | Hughes et al. 2011  [48] (N = 250)  *Grade 3* | 172 (69) | ≥48 | mg/24 h  Median  (10–90th percentile) | 333.0  (99.2–2,200.0)  (n = 77) | 370.0  (100.0–1,700.0)  (n = 77) | NC (p = 0.465) |
|  | Kampmann et al. 2015  [49] (N = 45)  *Grade 3* | 21 (47) | Median 130  (115–150) | mg/24 h | NR  (n = 21)  Pts with proteinuria:  659.0 [889.0]  (n = 10) | 112.5 [35.8]  (n = 8)  Pts with proteinuria:  297.0 [376.7]  (n = 10) | NC (NS)  **↑ (p = 0.0342)** |
|  | Schiffmann et al. 2001 [36] (N = 14)  *Grade 1a* | 14 (100) | 5.5 | g/24 h | 5 pts with urinary protein excretion >1  (n = 14) | NR  (n = 14) | NC (NR) |
|  | Schiffmann et al. 2006  [39] (N = 25)  *Grade 1c* | 25 (100) | 48–54 | mg/24 h | 353 (100–7,500)  (n = 24) | Median: 543 (n = 20) | ↑ (NR) |
|  | Schiffmann et al. 2007  [52] (N = 12)  *Grade 2* | 12 (100) | 24–48 | mg/24 h | 1,217 [1,246]  (n = 12) | 1,485 [1,295]  (n = 12) | ↑ (NR) |
| **Beta** | Kim et al. 2016 [79] (N = 19)  *Grade 2* | 15 (79)  *11 adults,*  *4 paediatric pts* | 60–126 | mg/day | <0.1 g/day:  87 [11.9]  (n = 3)  >0.1 g/day:  1,352 [861.1]  (n = 6)  During shortage:  1,290 [1,480] | <0.1 g/day:  65.7 [40.1]  (n = 3)  >0.1 g/day:  1,319.7 [1,405.6]  (n = 6)  During shortage:  910 [1,100] | NC (NS)  NC (NS)  NC (p>0.05) |
|  | Lubanda et al. 2009  [72] (N = 21)  *Grade 1c* | 21 (100) | 5.5 | g protein/ g creatinine | Median: 0.3  (n = 20) | Median: 0.4  (n = 20) | **↑** (NS) |
|  | Mignani et al. 2004  [80] (N = 3)  *Grade 2* | 3 (100) | 18 | mg/day | ND in 2 pts, 550 in 1 pt  (n = 3) | ND  (n = 3) | ↓ (NR) |
|  | Najafian et al. 2016  [73] (N = 6)  *Grade 1c* | 6 (100) | 11–12 | g/g creatinine | Range: 0.10–1.62  (n = 6) | Range: <0.18–0.45  (n = 2) | ↓ (NR) |

Data are means [SD] or means ± SE or medians (range), unless otherwise indicated. Red font indicates statistically significant changes.

Case series, case reports, mixed-ERT publications, paediatric–adult mixed publications, and publications with other dose regimens are not included.

^a^ Total number of patients included in the study who were treated with ERT. ^b^ Study grades defined as follows: Grade 1a, randomized controlled trial; Grade 1c, single-arm clinical trial; Grade 2, prospective observational study; Grade 3, retrospective observational study. ^c^ Number of male patients who were treated with ERT. ^d^ Number of male, ERT-treated patients with data for the outcome at baseline. ^e^ Number of male, ERT-treated patients with data for the outcome at endpoint.

↓, decrease; ↑, increase; ERT, enzyme replacement therapy; h, hours; NC, no change; ND, not determined; NR, not reported; NS, not significant; pt, patient; SD, standard deviation; SE, standard error.

# Supplementary Table 9 Left ventricular hypertrophy outcomes with approved doses of agalsidase alfa and agalsidase beta in adult male patients

|  | **Study, year [reference]**  **(number of patients^a^)**  ***Evidence grade*^b^** | **Male, n (%)^c^** | **Duration (months)** | **Units** | **Baseline**  **(number of patients^d^)** | **End-point**  **(number of patients^e^)** | **Overall result (p value/ 95% CI)** |
| --- | --- | --- | --- | --- | --- | --- | --- |
| **Alfa** | Beck et al. 2015  [41] (N = 677)  *Grade 3* | 360 (53) | 60 | g/m^2.7^/year  (mean annualized LVMi slope ± SEM) | NA | Total:  0.33 ± 0.10  (n = 71)  LVH at BL:  0.19 ± 0.16  (n = 29)  No LVH at BL:  0.47 ± 0.13  (n = 42) | **↑ (95% CI 0.13, 0.53)**  NC (95% CI −0.13, 0.50)  **↑ (95% CI 0.22, 0.72)** |
|  | Goláň L. et al. 2015  [27] (N = 44)  *Grade 1a* | 26 (59) | 12 | LVMi, g/m^2^ (echo) | 80.1  (n = 14) | 87.4  (n = 10) | ↑ (95% CI  −1.81, 16.21) |
|  | Hughes et al. 2008  [29] (N = 7)  *Grade 1a* | 7 (100) | 6 | LVM, g (echo)  LVMi, g/m^2^ (echo)  [normal: 134]  LVM, g (MRI)  [mean] | NR  176 ± 10  (n = 7)  NR | −20.4 ± 27.2  (n = 6)  −6.4  (n = 6)  −11.5 g  (n = 6) | ↓ (NR)  ↓ (NR)  ↓ (NR) |
|  | Hughes et al. 2011  [48] (N = 250)  *Grade 3* | 172 (69) | ≥48 | LVMi, g/m^2.7^ (echo) | 54.7 [24.0]  (n = 45)  LVH at BL:  NR  (n = 22)  Without LVH at BL:  NR  (n = 23) | 52.2 [19.2]  (n = 45)  LVH at BL:  Mean difference:  −9.11  (n = 22)  Without LVH at BL:  Mean difference: 3.82  (n = 23) | **↓** (p = 0.247)  **↓ (p = 0.0115)**  ↑ (p = 0.0741) |
| **Alfa** | Kampmann et al. 2015  [49] (N = 45)  *Grade 3* | 21 (47) | Median 130  (115–150) | LVMi, g/m^2.7^ (echo) | LVMi <50 (% pts):  71  LVMi ≥50 (% pts): 29  (n = 21) | LVMi <50:  NC  (n = 15)  LVMi ≥50:  LS mean: −13.55  (n = 6) | NC (NR)  **↓ (p = 0.0061; 95% CI −23.05, −4.06)** |
|  | Tsuboi et al. 2012  [189] (N = 11)  *Pre-treatment with agalsidase beta for a minimum of 24 months*  *Grade 2* | 4 (36) | 12 | LVMi, g/m^2.7^ (echo) | 53.93 [10.32]  (n = 4) | 49.75 [11.54]  (n = 4) | ↓ (p = 0.3750) |
| **Beta** | Elliott et al. 2006  [76] (N = 5)  *Grade 2* | 5 (100) | 10.1 [2.3] | LVMi, g/m^2^ (echo) | 226. 3 [54.11]  (n = 5) | 226.7 [39.0]  (n = 5) | NC (NS) |
|  | Germain et al. 2013  [7] (N = 163)  *Grade 3* | 163 (100) | ERT group:  59 [22]  (n = 115) | LVM slope, g/year (echo) | ERT (18–29 years):  9.5 ± 2.36  (n = 15)  ERT (30–39 years):  8.4 ± 3.55  (n = 17)  ERT (40–49 years):  13.4 ± 6.63  (n = 7)  ERT (≥50 years):  0.4 ± 9.41  (n = 9) | ERT (18–29 years):  −3.6 ± 1.62  (n = 31)  ERT (30–39 years):  2.8 ± 2.2  (n = 44)  ERT (40–49 years):  3.4 ± 2.87  (n = 23)  ERT (≥ 50 years):  7.7 ± 4.48  (n = 17) | **↓ (p < 0.0001)**  ↓ (p = 0.1760)  ↓ (p = 0.1691)  ↑ (p = 0.4843) |
|  | Kim et al. 2016 [79] (N = 19)  *Grade 2* | 15 (79)  *11 adults,*  *4 paediatric pts* | 60–126 | LVMi, g/m^2.7^ (echo) | 59.7 [26.2]  (n = 11) | 57 [22.4]  (n = 11)  Pts with LVH  (mean change/year): −2.0 [1.6]  (n = 4)  Pts without LVH  (mean change/year):  0.5 [1.4]  (n = 5) | NC (NR) |
|  | Mignani et al. 2004  [80] (N = 3)  *Grade 2* | 3 (100) | 18 | LVM, g (echo) | 403.8–934.6 | 402.4–832.1 | ↓ (NR) |
|  | Motwani et al. 2012  [81] (N = 66)  *Grade 3* | 44 (67) | 36 | LVMi,  g/m^2^ (echo) | 123 [2]  (n = 44) | 120 [26]  (n = 44) | **↓ (p < 0.001)** |

Data are means [SD] or means ± SE or medians (range), unless otherwise indicated. Red font indicates statistically significant changes.

Case series, case reports, mixed-ERT publications, paediatric–adult mixed publications, and publications with other dose regimens are not included.

^a^ Total number of patients included in the study who were treated with ERT. ^b^ Study grades defined as follows: Grade 1a, randomized controlled trial; Grade 2, prospective observational study; Grade 3 retrospective observational study. ^c^ Number of male patients who were treated with ERT. ^d^ Number of male, ERT-treated patients with data for the outcome at baseline. ^e^ Number of male, ERT-treated patients with data for the outcome at endpoint.

↓, decrease; ↑, increase; BL, baseline; CI, confidence interval; echo, echocardiography; ERT, enzyme replacement therapy; LS, least squares; LVH, left ventricular hypertrophy; LVM, left ventricular mass; LVMi, left ventricular mass index; NA. not available; NC, no change; NR, not reported; NS, not significant; pt, patient; SD, standard deviation; SE, standard error; SEM, standard error of the mean.

# Supplementary Table 10 Wall thickness outcomes with approved doses of agalsidase alfa and agalsidase beta in adult male patients

|  | **Study, year [reference]**  **(number of patients^a^)**  ***Evidence grade*^b^** | **Male, n (%)^c^** | **Duration (months)** | **Units** | **Baseline (number of patients^d^)** | **End-point (number of patients^e^)** | **Overall result (p value/ 95% CI)** |
| --- | --- | --- | --- | --- | --- | --- | --- |
| **Alfa** | Kampmann et al. 2015  [49] (N = 45)  *Grade 3* | 21 (47) | Median 130 (115–150) | MWT, mm (echo) | 12.3 [2.9]  (n = 21) | LS mean change:  −1.89  (n = 21) | **↓ (p < 0.0001, 95% CI −2.58, −1.19)** |
|  | Tsuboi et al. 2012  [189] (N = 11)  *Pre-treatment with agalsidase beta for a minimum of 24 months*  *Grade 2* | 4 (36) | 12 | LVWT, mm (echo) | 13.2  (n = 4) | 13.0  (n = 4) | ↓ (NR) |
| **Beta** | Elliott et al. 2006  [76] (N = 5)  *Grade 2* | 5 (100) | 10.1 [2.3] | Maximum LVWT, mm | NR  (n = 5) | 0.02 [0.3]  (n = 5) | NC (NS) |
|  | Mignani et al. 2004  [80] (N = 3)  *Grade 2* | 3 (100) | 18 | PWT, cm (echo) | 1.19–2.30 | 1.20–2.20 | ↑↓ (NR) |
|  | Motwani et al. 2012  [81] (N = 66)  *Grade 3* | 44 (67) | 36 | MWT, mm (echo) | 15 [7]  (n = 44) | 14 [5]  (n = 44) | **↓ (p < 0.001)** |

Data are means [SD] or means ± SE or medians (range), unless otherwise indicated. Red font indicates statistically significant changes.

Case series, case reports, mixed-ERT publications, paediatric–adult mixed publications, and publications with other dose regimens are not included.

^a^ Total number of patients included in the study who were treated with ERT. ^b^ Study grades defined as follows: Grade 2 prospective observational study; Grade 3 retrospective observational study. ^c^ Number of male patients who were treated with ERT. ^d^ Number of male, ERT-treated patients with data for the outcome at baseline. ^e^ Number of male, ERT-treated patients with data for the outcome at endpoint.

↓, decrease; ↑, increase; CI, confidence interval; echo, echocardiography; ERT, enzyme replacement therapy; LS, least squares; LVWT, left ventricular wall thickness; MWT, maximal wall thickness; NC, no change; NR, not reported; NS, not significant; PWT, posterior wall thickness; SD, standard deviation; SE, standard error.

# Supplementary Table 11 Ejection fraction outcomes with approved doses of agalsidase alfa and agalsidase beta in adult male patients

|  | **Study, year [reference]**  **(number of patients^a^)**  ***Evidence grade*^b^** | **Male, n (%)^c^** | **Duration**  **(months)** | **Units** | **Baseline (number of patients^d^)** | **End-point**  **(number of patients^e^)** | **Overall result (p value)** |
| --- | --- | --- | --- | --- | --- | --- | --- |
| **Alfa** | Hughes et al. 2008  [29] (N = 7)  *Grade 1a* | 7 (100) | 6 | EF, % (echo) | 78.7 ± 1.71  (n = 7) | 81 ± 2.74  (n = 7) | NC (NR) |
|  | Kampmann et al. 2015  [49] (N = 45)  *Grade 3* | 21 (47) | Median 130 (115–150) | LVEF, % | 69.9 [7.3]  (n = 21) | 69.8 [7.0]  (n = 21) | NC  (p = 0.4546) |
| **Beta** | Mignani et al. 2004  [80] (N = 3)  *Grade 2* | 3 (100) | 18 | LVEF, % (echo) | 40.0–65.2 | 54.0–64.0 | ↑ (NR) |
|  | Motwani et al. 2012  [81] (N = 66)  *Grade 3* | 44 (67) | 36 | EF, %  (echo) | 62 [4]  (n = 44) | 64 [3]  (n = 44) | **↑ (p < 0.001)** |

Data are means [SD] or means ± SE or medians (range), unless otherwise indicated. Red font indicates statistically significant changes.

Case series, case reports, mixed-ERT publications, paediatric–adult mixed publications, and publications with other dose regimens are not included.

^a^ Total number of patients included in the study who were treated with ERT. ^b^ Study grades defined as follows: Grade 1a, randomized controlled trial; Grade 2, prospective observational study; Grade 3, retrospective observational study. ^c^ Number of male patients who were treated with ERT. ^d^ Number of male, ERT-treated patients with data for the outcome at baseline. ^e^ Number of male, ERT-treated patients with data for the outcome at endpoint.

↓, decrease; ↑, increase; echo, echocardiography; EF, ejection fraction; ERT, enzyme replacement therapy; LVEF, left ventricular ejection fraction; NC, no change; NR, not reported; SD, standard deviation; SE, standard error.

# Supplementary Table 12 Electrocardiography outcomes with approved doses of agalsidase alfa and agalsidase beta in adult male patients

|  | **Study, year [reference]**  **(number of patients^a^)**  ***Evidence grade*^b^** | **Male, n (%)^c^** | **Duration (months)** | **Units** | **Baseline**  **(number of patients^d^)** | **End-point**  **(number of patients^e^)** | **Overall result (p value)** |
| --- | --- | --- | --- | --- | --- | --- | --- |
| **Alfa** | Hughes et al. 2008  [29] (N = 7)  *Grade 1a* | 7 (100) | 6 | ms  (change in QRS duration) | NR  (n = 7) | ↓ by 12.9 [11.8]  (n = 7) | ↓ (NR) |
|  | Kampmann et al. 2015  [49] (N = 45)  *Grade 3* | 21 (47) | Median 130 (115–150) | Conduction abnormalities | NR | One male pt developed conduction abnormalities | ↑ (NR) |
|  | Schiffmann et al. 2001 [36] (N = 14)  *Grade 1a* | 14 (100) | 5.5 | ms  (change in QRS duration, echo) | 94.1 ± 4.85  (n = 14) | 91.7 ± 2.14  (n = 14) | ↓ (NR) |
| **Beta** | Eng et al. 2001 [69] (N = 15)  *Grade 1c* | 15 (100) | 5 infusions | NR | NR | NC | NC |
|  | Mignani et al. 2004  [80] (N = 3)  *Grade 2* | 3 (100) | 18 | PR interval, ms | 134–170 | 143–168 | NC (NR) |
|  | Motwani et al. 2012  [81] (N = 66)  *Grade 3* | 44 (67) | 36 | ms | PQ interval:  129 [10]  (n = 44)  P-wave duration:  76 [4]  (n = 44)  QRS width:  95 [14]  (n = 40)  QTc interval:  421 [19]  (n = 40) | PQ interval:  142 [12]  (n = 44)  P-wave duration:  90 [6]  (n = 44)  QRS width:  92 [16]  (n = 40)  QTc interval:  412 [16]  (n = 40) | **↑ (p < 0.001)**  **↑ (p < 0.001)**  ↓ (p = 0.06)  **↓ (p < 0.001)** |
|  |  |  |  | RE score^6^ | RE score:  5 (0-11)  (n = 44) | RE score:  5.5 (0–11)  (n = 44) | NC (p = 0.59) |

Data are means [SD] or means ± SE or medians (range), unless otherwise indicated. Red font indicates statistically significant changes.

Case series, case reports, mixed-ERT publications, paediatric–adult mixed publications, and publications with other dose regimens are not included.

^a^ Total number of patients included in the study who were treated with ERT. ^b^ Study grades defined as follows: Grade 1a, randomized controlled trial; Grade 1c, single-arm clinical trial; Grade 2, prospective observational study; Grade 3, retrospective observational study. ^c^ Number of male patients who were treated with ERT. ^d^ Number of male, ERT-treated patients with data for the outcome at baseline. ^e^ Number of male, ERT-treated patients with data for the outcome at endpoint.

↓, decrease; ↑, increase; ECG, electrocardiogram; echo, echocardiography; ERT, enzyme replacement therapy; LVH, left ventricular hypertrophy; NC, no change; NR, not reported; pt, patient; QTc, heart rate-corrected QT interval; RE, Romhilts–Estes (categorizes ECG as no-, probable-, or definite LVH); SD, standard deviation; SE, standard error.

# Supplementary Table 13 Sweat function outcomes with approved doses of agalsidase alfa and agalsidase beta in adult male patients

|  | **Study, year [reference]**  **(number of patients^a^)**  ***Evidence grade*^b^** | **Male, n (%)^c^** | **Duration (months)** | **Units** | **Baseline**  **(number of patients^d^)** | **End-point (number of patients^e^)** | **Overall result (p value)** |
| --- | --- | --- | --- | --- | --- | --- | --- |
| **Alfa** | Bongiorno et al. 2003 [42] (N = 4)  *Grade 2* | 4 (100) | 12 | NR | NR  (n = 4) | Improvement in hypohidrosis (n = 4) | ↑ (NR) |
|  | Gupta et al. 2008  [45] (N = 49)  *Grade 2* | 27 (55) | 36 | Skin moisture | NR | No improvement | NC (NR) |
|  | Hughes et al. 2011  [48] (N = 250)  *Grade 3* | 172 (69) | ≥48 | Pts with hypohidrosis, %  Pts with hyperhidrosis, % | 45.4  (n = 97)  1.1  (n = 89) | 59.8  (n = 97)  2.2  (n = 89) | **↑ (NR)**  **↑ (NR)** |
|  | Jardim et al. 2006  [31] (N = 7)  *Grade 1c* | 7 (100) | 24 | Sweating | Score: NR  (n = 7) | Improvement  (n = 5) | **↑ (NR)** |
|  | Schiffmann et al. 2003  [37] (N = 26)  *Grade 1c* | 26 (100) | 36 | µL/mm^2^ sweat excretion  (normal: 1.05 [0.81]) | 0.24 [0.33]  (n = 17) | 24–72 h post-infusion:  0.57 [0.71]  (n = 17) | **↑ (p = 0.04)** |
|  | Schiffmann et al. 2007  [52] (N = 12)  *Grade 2* | 12 (100) | 24–48 | µL/mm^2^ | NR  (n = 12) | 0.29 [0.22]  (n = 12) | NR |
| **Beta** | Eng et al. 2001  [69] (N = 15)  *Grade 1c* | 15 (100) | 5 infusions | NR | NR | Increased ability to sweat | ↑ (NR) |
|  | Hilz et al. 2004  [77] (N = 22)  *Grade 2* | 22 (100) | 18–23 | NR | NR  (n = 22) | All pts reported subjective improvement in sweating  (n = 22) | ↑ (NR) |

Data are means [SD] or means ± SE or medians (range), unless otherwise indicated. Red font indicates statistically significant changes. Case series, case reports, mixed-ERT publications, paediatric–adult mixed publications, and publications with other dose regimens are not included.

^a^ Total number of patients included in the study who were treated with ERT. ^b^ Study grades defined as follows: Grade 1c, single-arm clinical trial; Grade 2, prospective observational study; Grade 3, retrospective observational study. ^c^ Number of male patients who were treated with ERT. ^d^ Number of male, ERT-treated patients with data for the outcome at baseline. ^e^ Number of male, ERT-treated patients with data for the outcome at endpoint.

↓, decrease; ↑, increase; ERT, enzyme replacement therapy; h, hours; NC, no change; NR, not reported; pt, patient; SD, standard deviation; SE, standard error.

# Supplementary Table 14 Nerve sensitivity outcomes with approved doses of agalsidase alfa and agalsidase beta in adult male patients

|  | **Study, year [reference]**  **(number of patients^a^)**  ***Evidence grade*^b^** | **Male, n (%)^c^** | **Duration (months)** | **Units** | **Baseline**  **(number of patients^d^)** | **End-point**  **(number of patients^e^)** | **Overall result (p value/ 95% CI)** |
| --- | --- | --- | --- | --- | --- | --- | --- |
| **Alfa**  **Alfa** | Schiffmann et al. 2003  [37] (N = 26)  *Grade 1c* | 26 (100) | 36 | Nerve conduction tests  Sensory tests | 5 pts: focal nerve compression  Foot:  Warm: elevated  Cold: elevated  Vibration: normal  (n = 25)  Hand:  Warm: normal  Cold: elevated  Vibration: elevated  (n = 25) | High individual variability  3 pts : ↑ 40%  3 pts: ↓ 40%  Overall, no significant changes observed  (n = 25)  Foot:  Cold sensation: reduction  Warm sensation: reduction  Vibration: increase  (n = 22)  Hand:  Warm: NC  Cold: reduction  Vibration: NC  (n = 25) | NC (NR)  **↓ (p < 0.001)**  **↓ (p = 0.006)**  **↑ (p = 0.006)**  NC (NR)  ↓ (p = 0.08)  NC (NR) |
|  | Jardim et al. 2004  [30] (N = 8)  *Grade 1c* | 7 (88) | 12 | Neurological examination PNS score | Mean: 2.37  (n = 8) | Mean: 1.87  (n = 7) | NC (NR) |
|  | Jardim et al. 2006  [31] (N = 7)  *Grade 1c* | 7 (100) | 24 | Neurological examination PNS score | Score: NR  (n = 7) | NC  (n = 5) | NC (NR) |
|  | Schiffmann et al. 2006  [38] (N = 26)  *Grade 1c* | 26 (100) | 18 | fibres/mm  (IENFD, thigh)  Thermal threshold  (JND units) | 5.56 [4.12]  (n = 14)  Cold sensation:  18.3 [4.3]  (n = 14)  Warm sensation:  14.1 [2.4]  (n = 14) | 3.85 [2.84]  (n = 13)  Cold sensation:  18.5 [4.6]  (n = 13)  Warm sensation:  13.7 [3.7]  (n = 12) | **↓ (p < 0.05, 95% CI  −0.2497, −3.106)**  NC (NR)  NC (NR) |
| **Beta** | Hilz et al. 2004  [77] (N=22)^f^  *Grade 2* | 22 (100) | 18–23 | VDT, JND on the first toe  CDT, JND (dorsum of feet)  HP 0.5, JND (dorsum of feet)  HP 5.0, JND (dorsum of feet) | 15.5 [3.5]  (n = 22)  19.8 [11.1]  (n = 22)  22.3 [6.7]  (n = 22)  27.3 [5.6]  (n = 22) | 14.3 [4.1]  (n = 22)  19.9 [10.2]  (n = 22)  19.4 [1.3]  (n = 22)  22.5 [2.3]  (n = 22) | **↓ (p < 0.05)**  NC (p>0.05)  **↓ (p < 0.01)**  **↓ (p < 0.01)** |

Data are means [SD] or means ± SE or medians (range), unless otherwise indicated. Red font indicates statistically significant changes.

Case series, case reports, mixed-ERT publications, paediatric–adult mixed publications, and publications with other dose regimens are not included.

^a^ Total number of patients included in the study who were treated with ERT. ^b^ Study grades defined as follows: Grade 1c, single-arm clinical trial; Grade 2, prospective observational study. ^c^ Number of male patients who were treated with ERT. ^d^ Number of male, ERT-treated patients with data for the outcome at baseline. ^e^ Number of male, ERT-treated patients with data for the outcome at endpoint. ^f^ Normal values for VDT, JND: 12.75 [2.73] (upper limit: 19.58); CDT, JND: 9.64 [3.57] (upper limit: 18.75); HP 0.5, JND: 17.05 [2.36] (upper limit: 22.95); HP 5.0, JND: 20.52 [1.43] (upper limit: 24.9).

↓, decrease; ↑, increase; CDT, cold-detection threshold; CI, confidence interval; ERT, enzyme replacement therapy; HP, heat-pain; IENFD, intraepidermal nerve fibre density; JND, just noticeable difference; NC, no change; NR, not reported; pt, patient; VDT, vibration detection threshold; SD, standard deviation; SE, standard error.

# Supplementary Table 15 Vestibular/auditory and other central nervous system outcomes with approved doses of agalsidase alfa and agalsidase beta in adult male patients

|  | **Study, year [reference]**  **(number of patients^a^)**  ***Evidence grade*^b^** | **Male, n (%)^c^** | **Duration (months)** | **Units** | **Baseline**  **(number of patients^d^)** | **End-point**  **(number of patients^e^)** | **Overall result (p value)** |
| --- | --- | --- | --- | --- | --- | --- | --- |
| **Alfa** | Hajioff et al. 2003  [28] (N = 15)  *Grade 1a/c* | 15 (100) | 24–30 | dB (high-frequency SNHL) | Overall hearing loss, Median (IQR):  25 (15.0-49.9)  (n = 15) | Median: 4.9  (n = 20) | **↑ (p = 0.004 vs BL)** |
|  | Palla et al. 2003  [34] (N = 21)  *Grade 1c* | 13 (62) | 12 | Peripheral vestibular function  (average gain) | Reduced gain in 85% of pts  (n = 13) | Improvement  (n = 5) | ↑ (NS) |
|  | Moore et al. 2002  [33] (N = 26 pts)  *Grade 1a* | 26 (100) | 6 | mL/min/100 g tissue  (regional CBF) | Resting CBF:  25.7 [3.8]  (n = 26)  Visual activation CBF:  29.4 [3.4]  (n = 26) | Resting CBF:  −2.3 [3.7]  (n = 14)  Visual activation:  −1.75 [2.6]  (n = 14) | NR  ↓ NR |
|  | Moore et al. 2001  [32] (N = 26)  *Grade 1a* | 26 (100) | 6 | mL/min/100 g tissue (resting global CBF) | CBF Fabry group:  42 [4.8]  (n = 26) | ↓ by 3.48 [4.57]  (n = 14) | **↓ (NR)** |
|  | Jardim et al. 2004  [30] (N = 8)  *Grade 1c* | 7 (88) | 12 | Neurological examination CNS score | Mean: 2.07  (n = 8) | Mean: 1.8  (n = 7) | NC (NR) |
|  | Sergi et al. 2010  [53] (N = 20)  *Grade 2* | 11 (55) | 51.5  (25–73) | Hearing threshold (dB nHL)  HFHL | 30.7  37.5 (16) | 34.7  36.96 (15.6) | NC (p = 0.5)  NC (NR) |
| **Beta** | Hilz et al. 2004  [77] (N = 22)  *Grade 2* | 22 (100) | 18–23 | Neurological examination | Normal for cranial nerve function, muscle strength, coordination  (n = 20)  Reduced deep tendon reflexes  (n = 2) | No changes reported  (n = 22) | NC (NR) |

Data are means [SD] or means ± SE or medians (range), unless otherwise indicated. Red font indicates statistically significant changes.

Case series, case reports, mixed-ERT publications, paediatric–adult mixed publications, and publications with other dose regimens are not included.

^a^ Total number of patients included in the study who were treated with ERT. ^b^ Study grades defined as follows: Grade 1a randomized controlled trial; Grade 1c single-arm clinical trial; Grade 1a/c randomized controlled trial with single-arm open-label extension; Grade 2 prospective observational study. ^c^ Number of male patients who were treated with ERT. ^d^ Number of male, ERT-treated patients with data for the outcome at baseline. ^e^ Number of male, ERT-treated patients with data for the outcome at endpoint.

↓, decrease; ↑, increase; BL, baseline; CBF, cerebral blood flow; CNS, central nervous system; dB, decibel; ERT, enzyme replacement therapy; HFHL, high-frequency hearing loss; IQR, inter-quartile range; NC, no change; nHL, normalized hearing loss; NR, not reported; NS, not significant; pt, patient; SD, standard deviation; SE, standard error; SNHL, sensorineural hearing loss.

# Supplementary Table 16 Pain outcomes with approved doses of agalsidase alfa and agalsidase beta in adult male patients

|  | **Study, year [reference]**  **(number of patients^a^)**  ***Evidence grade*^b^** | **Male, n (%)^c^** | **Duration**  **(months)** | **Units** | **Baseline**  **(number of patients^d^)** | **End-point**  **(number of patients^e^)** | **Overall result (p value)** |
| --- | --- | --- | --- | --- | --- | --- | --- |
| **Alfa** | Bongiorno et al. 2003  [42] (N = 4)  *Grade 2* | 4 (100) | 12 | NR | NR | Improvement in acroparaesthesia | ↓ (NR) |
|  | Hoffmann et al. 2007  [47] (N = 714)  *Grade 3* | 345 (48) | ≤36 | Change in BPI score | NR | Pain at its worst:  −0.4 (0.4)  (n = 51)  Pain on average:  −0.6 (0.4)  (n = 51  Pain at its least:  −0.2 (0.3)  (n = 51)  Present pain:  –1.1 (0.5)  (n = 51) | NC (NS)  **↓ (p < 0.05)**  NC (NS)  **↓ (p < 0.05)** |
|  | Hughes et al. 2011  [48] (N = 250)  *Grade 3* | 172 (69) | >48 | BPI score | Pain at its worst:  3.7 [3.0]  (n = 46)  Pain on average:  2.7 [2.2]  (n = 46) | Pain at its worst:  3.5 [3.1]  (n = 46)  Pain on average:  2.6 [2.4]  (n = 46) | NC (p = 0.803)  NC (p = 0.762) |
|  | Jardim et al. 2006  [31] (N = 7)  *Grade 1c* | 7 (100) | 24 | Acroparaesth-esia | Score: NR  (n = 7) | General improvement  (n = 5) | ↓ (NR) |
|  | Schiffmann et al. 2001 [36] (N = 14)  *Grade 1a* | 14 (100) | 5.5 | BPI severity  Pain-related QoL | 3.8 ± 0.44  (n = 14)  3.2 ± 0.55  (n = 14) | 2.7 ± 0.54  (n = 14)  2.1 ± 0.56  (n = 14) | ↓ (NR)  ↓ (NR) |
| **Alfa** | Schiffmann et al. 2003  [37] (N = 26)  *Grade 1c* | 26 (100) | 36 | BPI score | 18-month time-point:  NR  (n = 25) | 18-month time-point:  ↓ 1.9 [0.53)  (n = 25) | **↓ (p = 0.003)** |
|  | Sergi et al. 2010  [53] (N = 20)  *Grade 2* | 11 (55) | 51.5  (25–73) | Pain severity  (no. of pts) | Mild: n = 8  Moderate: n = 1  Severe: n = 2 | Mild: n = 6  Moderate: n = 3  Severe: n = 2 | NR |
|  | Whitfield et al. 2005  [55] (N = 8)  *Grade 2* | 6 (75) | 12 | BPI score | Average pain:  0–4  (n = 5)  Worst pain last 24 h:  0–7  (n = 5) | Average pain:  0–4  (n = 5)  Worst pain last 24 h:  0–4  (n = 5) | NC (NR)  ↓ (NR) |
| **Beta** | Eng et al. 2001  [69] (N = 15)  *Grade 1c* | 15 (100) | 5 infusions | Short Form McGill Pain | NR | Overall pain: improved  (n = 3)  Present pain intensity: improved  (n = 3) | **↑ (p = 0.03)**  **↑ (p = 0.004)** |
|  | Eto et al. 2005  [70] (N = 13)  *Grade 1c* | 13 (100) | 4.6 | PPI score | NR  (n = 13) | Slight reduction in all median pain scores  (n = 13)  Reduction in PPI  (n = 13) | ↓ (NS)  ↓ (p = 0.0630) |
|  | Hilz et al. 2004  [77] (N=22)  *Grade 2* | 22 (100) | 18–23 | TSS | 1.74 [1.97]  (n = 22) | Improvement:  0.83 [1.53]  (n = 22) | **↓ (p = 0.038)** |
|  | Mignani et al. 2004  [80] (N = 3)  *Grade 2* | 3 (100) | 18 | Pain and paraesthesia | Present in 1 pt  (n = 3) | Absent in all pts  (n = 3) | ↓ (NR) |

Data are means [SD] or means ± SE or medians (range), unless otherwise indicated. Red font indicates statistically significant changes. Case series, case reports, mixed-ERT publications, paediatric–adult mixed publications, and publications with other dose regimens are not included.

^a^ Total number of patients included in the study who were treated with ERT. ^b^ Study grades defined as follows: Grade 1a, randomized controlled trial; Grade 1c, single-arm clinical trial; Grade 2, prospective observational study; Grade 3, retrospective observational study. ^c^ Number of male patients who were treated with ERT. ^d^ Number of male, ERT-treated patients with data for the outcome at baseline. ^e^ Number of male, ERT-treated patients with data for the outcome at endpoint.

↓, decrease; ↑, increase; BPI, Brief Pain Inventory; ERT, enzyme replacement therapy; h, hours; NC, no change; NR, not reported; NS, not significant; PPI, present pain intensity; pt, patient; QoL, quality of life; SD, standard deviation; SE, standard error; TSS, Total Symptom Score.

# Supplementary Table 17 Gastrointestinal outcomes with approved dose of agalsidase alfa in adult male patients

|  | **Study, year [reference]**  **(number of patients^a^)**  ***Evidence grade*^b^** | **Male, n (%)^c^** | **Duration**  **(months)** | **Units** | **Baseline (number of patients^d^)** | **End-point**  **(number of patients^e^)** | **Overall result (p value)** |
| --- | --- | --- | --- | --- | --- | --- | --- |
| **Alfa** | Hughes et al. 2011  [48] (N = 250)  *Grade 3* | 172 (69) | ≥48 | Pts, % | Abdominal pain:  61.8 (n = 76)  Constipation:  17.2 (n = 64)  Diarrhoea:  61.0 (n = 77)  Nausea:  34.3 (n = 70)  Vomiting:  22.4 (n = 67) | Abdominal pain:  59.2 (n = 76)  Constipation:  12.5 (n = 64)  Diarrhoea:  62.3 (n = 77)  Nausea:  32.9 (n = 70)  Vomiting:  20.9 (n = 67) | NR  NR  NR  NR  NR |
|  | Jardim et al. 2006  [31] (N = 7)  *Grade 1c* | 7 (100) | 24 | # of episodes, diarrhoea | Score: NR  (n = 7) | Resolved  (n = 5) | ↓ (NR) |

Data are means [SD] or means ± SE or medians (range), unless otherwise indicated. Red font indicates statistically significant changes.

Case series, case reports, mixed-ERT publications, paediatric–adult mixed publications, and publications with other dose regimens are not included.

^a^ Total number of patients included in the study who were treated with ERT. ^b^ Study grades defined as follows: Grade 1a, randomized controlled trial; Grade 3, retrospective observational study. ^c^ Number of male patients who were treated with ERT. ^d^ Number of male, ERT-treated patients with data for the outcome at baseline. ^e^ Number of male, ERT-treated patients with data for the outcome at endpoint.

↓, decrease in symptoms; ERT, enzyme replacement therapy; SD, standard deviation; SE, standard error; NR, not reported; pt, patient.

# Supplementary Table 18 Quality of life outcomes with approved doses of agalsidase alfa and agalsidase beta in adult male patients

|  | **Study, year [reference]**  **(number of patients^a^)**  ***Evidence grade*^b^** | **Male, n (%)^c^** | **Duration (months)** | **Units** | **Baseline**  **(number of patients^d^)** | **End-point**  **(number of patients^e^)** | **Overall result (p value)** |
| --- | --- | --- | --- | --- | --- | --- | --- |
| **Alfa** | Hughes et al. 2011  [48] (N = 250)  *Grade 3* | 172 (69) | ≥48 | EQ VAS health score  (100 mm) | 67.7 [21.7]  (n = 30) | 71.1 [17.6]  (n = 30) | **↑** (p = 0.346) |
|  |  |  |  | EQ-5D health score | 0.63 [0.3]  (n = 37) | 0.72 [0.2]  (n = 37) | **↑** (p = 0.079) |
|  | Ghali et al. 2012  [100] (N = 40)  *Grade 3*  *Pre-treatment with agalsidase beta (minimum of 12 months)* | 32 (80) | 12 | Self-reported energy levels  scored 1–10 using SF-36 | 8 (range 4–9)  (n = 9) | 7 (range 5–9)  (n = 9) | NC (p = 0.77) |
| **Beta** | Eng et al. 2001  [69] (N = 15)  *Grade 1c* | 15 (100) | 5 infusions | SF-36 QoL | NR | Improvements reported for: bodily pain, general health, vitality  (n = 3) | ↑ (NR) |
|  | Eto et al. 2005  [70] (N = 13)  *Grade 1c* | 13 (100) | 4.6 | SF-36 QoL | Role Emotional:  NR  (n = 13)  General Health:  NR  (n = 13)  Mental Health:  NR  (n = 13) | Role Emotional:  NR  (n = 13)  General Health:  NR  (n = 13)  Mental Health:  NR  (n = 13) | ↑ (p = 0.063)  **↑ (p = 0.023)**  **↑ (p = 0.048)** |
|  | Watt et al. 2010  [83] (N = 130)  *Grade 3* | 71 (55) | 81 [31] | SF-36 QoL | (n = 71 for all subscales)  Physical summary score:  38.8 ± 1.29  Mental summary  score:  46.0 ± 1.22 | (n = 71 for all subscales)  Physical summary score:  >24–36 months:  41.3 ± 1.29  Mental summary  score:  >24–36 months:  47.3 ± 1.22 | **↑** (NR)  **↑** (NR) |

Data are means (SD) or means ± SE or medians (range), unless otherwise indicated. Red font indicates statistically significant changes.

Case series, case reports, mixed-ERT publications, paediatric–adult mixed publications, and publications with other dose regimens are not included.

^a^ Total number of patients included in the study who were treated with ERT. ^b^ Study grades defined as follows: Grade 1c, single-arm clinical trial; Grade 3, retrospective observational study. ^c^ Number of male patients who were treated with ERT. ^d^ Number of male, ERT-treated patients with data for the outcome at baseline. ^e^ Number of male, ERT-treated patients with data for the outcome at endpoint.

↓, decrease in QoL; ↑, improvement in QoL; ERT, enzyme replacement therapy; EQ-5D, 5-dimension EuroQol questionnaire; EQ VAS, EuroQol visual analogue scale; NC, no change; NR, not reported; QoL, quality of life; SD, standard deviation; SE, standard error; SF-36, 36-Item Short-Form Health Survey.
